# Metabolic Responses of Normal Rat Kidneys to a High Salt Intake

**DOI:** 10.1093/function/zqad031

**Published:** 2023-06-22

**Authors:** Satoshi Shimada, Brian R Hoffmann, Chun Yang, Theresa Kurth, Andrew S Greene, Mingyu Liang, Ranjan K Dash, Allen W Cowley

**Affiliations:** Department of Physiology, Medical College of Wisconsin, Milwaukee, WI 53226, USA; Mass Spectrometry and Protein Chemistry, Protein Sciences, The Jackson Laboratory, Bar Harbor, ME 04609, USA; Department of Physiology, Medical College of Wisconsin, Milwaukee, WI 53226, USA; Department of Physiology, Medical College of Wisconsin, Milwaukee, WI 53226, USA; Mass Spectrometry and Protein Chemistry, Protein Sciences, The Jackson Laboratory, Bar Harbor, ME 04609, USA; Department of Physiology, Medical College of Wisconsin, Milwaukee, WI 53226, USA; Department of Physiology, Medical College of Wisconsin, Milwaukee, WI 53226, USA; Department of Biomedical Engineering, Medical College of Wisconsin and Marquette University, Milwaukee, WI 53226, USA; Department of Physiology, Medical College of Wisconsin, Milwaukee, WI 53226, USA

**Keywords:** kidney, metabolomics, transcriptomics, salt diet, Sprague–Dawley rat, aerobic glycolysis

## Abstract

In this study, novel methods were developed, which allowed continuous (24/7) measurement of arterial blood pressure and renal blood flow in freely moving rats and the intermittent collection of arterial and renal venous blood to estimate kidney metabolic fluxes of O_2_ and metabolites. Specifically, the study determined the effects of a high salt (HS; 4.0% NaCl) diet upon whole kidney O_2_ consumption and arterial and renal venous plasma metabolomic profiles of normal Sprague–Dawley rats. A separate group of rats was studied to determine changes in the cortex and outer medulla tissue metabolomic and mRNAseq profiles before and following the switch from a 0.4% to 4.0% NaCl diet. In addition, targeted mRNA expression analysis of cortical segments was performed. Significant changes in the metabolomic and transcriptomic profiles occurred with feeding of the HS diet. A progressive increase of kidney O_2_ consumption was found despite a reduction in expression of most of the mRNA encoding enzymes of TCA cycle. A novel finding was the increased expression of glycolysis-related genes in Cx and isolated proximal tubular segments in response to an HS diet, consistent with increased release of pyruvate and lactate from the kidney to the renal venous blood. Data suggests that aerobic glycolysis (eg, Warburg effect) may contribute to energy production under these circumstances. The study provides evidence that kidney metabolism responds to an HS diet enabling enhanced energy production while protecting from oxidative stress and injury.

Metabolomic and transcriptomic analysis of kidneys of Sprague-Dawley rats fed a high salt diet.

## Graphical Abstract

**Graphical Abstract:**
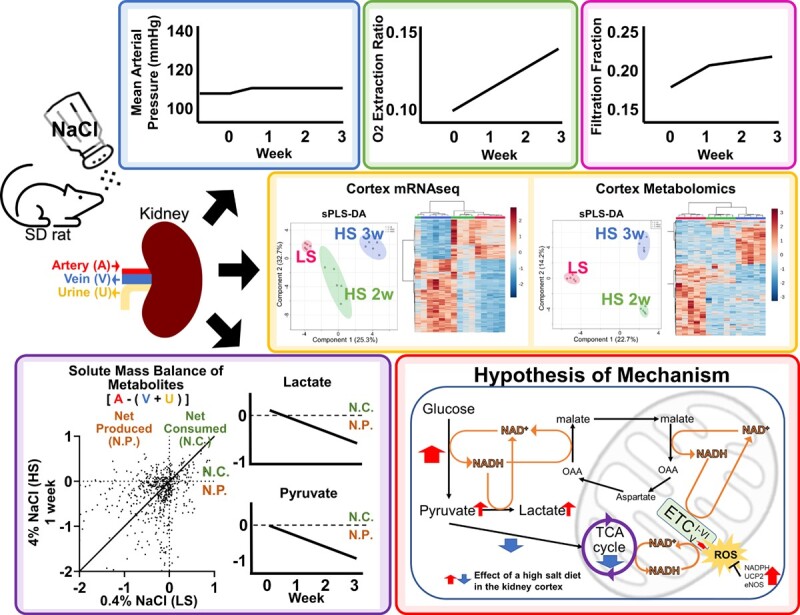

## Introduction

The conversion of daily food intake into energy and biomass is a complex process with some mechanisms operating relatively inefficiently as essential for short-term survival with others operating in more efficient and sustained ways required in face of sustained chronic stressors.^1–3^ Although malnourishment is one such chronic stressor, industrialized societies are largely faced with dietary excesses including condiments such as table salt (NaCl). Excess dietary salt is a well-recognized cardiovascular risk factor especially in those genetically susceptible to hypertension (“salt-sensitivity”). Half of hypertensive patients are blood pressure (BP) salt-sensitive^4–6^ who suffer nearly 3-times greater risk of chronic kidney disease (CKD).^7–9^

The underlying genetic and physiological determinants of BP salt-sensitivity remain incompletely understood although general progress has been made in understanding the contributions of kidney function, and of the neural and endocrine controllers of cardiovascular system determinants of sodium and water excretion.^10,11^ Recently, kidney metabolism has begun to emerge as a novel and important determinant of BP in salt-sensitive forms of hypertension.^12,13^ However, the dearth of studies examining kidney intermediary metabolism in hypertension is remarkable given the great importance of this organ in the regulation of salt and water in the long-term control of BP. A great deal of energy is required by the kidney to support renal tubular transport of sodium and other substances, which are all linked to the activity of Na^+^-K^+^-ATPase and H^+^ pumps on the basolateral membranes of renal tubules.^14–17^ Kidneys have one of the highest specific metabolic rates among all organs estimated in humans to be over 400 kcal/kg tissue/d, which is the same as the heart, twice as high as the liver and the brain, and much higher than other organs.^12,15^ The housekeeping role of intermediary metabolic processes required to convert nutritive substances to energy, cellular components, and waste products is well understood. However, it has been increasingly recognized that these metabolic pathways and intermediate products can influence gene expression, signal transduction, and other regulatory pathways.^18^ Intermediary metabolism and related mitochondrial and cellular functions are now recognized to play an essential role in the development of acute kidney injury and CKD.^19,20^

Relatively few studies have focused on these aspects of kidney function in response to excess dietary salt. Traditionally, studies of organ metabolism have been limited to examination of a relatively few metabolites.^21,22^ With the emergence of large-scale mass spectrometry and analysis tools (*aka* metabolomics), it is currently possible to identify and prioritize several thousands of detected features providing a comprehensive analysis in a tissue specimen of the changes in metabolism that occur across various organs of the body as recently demonstrated by Jang et al.^23^ who analyzed the arterial and venous blood of 11 organs in fasted pigs. Relevant to the current study, Rinschen et al.^13^ examined the effects of a high salt (HS) diet upon the glomerular and cortical tissue of Dahl salt-sensitive rats. However, with metabolomics yet in an infant stage, no one has even yet characterized kidney metabolism responses of a normal nonhypertensive rodent model to an HS diet. The present study utilized the commonly used outbred Sprague–Dawley (SD) rat model.

By definition, the metabolome represents the complete set of metabolites found in a biological sample built upon the genetic blueprint of an organism. As shown in the present study, even the most advanced mass spectrometry techniques as used in the present study can detect from 5000 to 6000 compounds and only about a sixth of those can be linked to Metaboanalyst database. This is in contrast to a transcriptome RNAseq analysis that provides 20–25 000 protein coding genes whose function is at least partially already known.

To overcome the current limitations of metabolomic analysis, a multi-omics approach was used in the present study in which RNA-seq transcriptional data were obtained in parallel to provide the metabolic pathway templates upon which to integrate changes of both gene expression and metabolites. Also, a novel system was developed, which allowed for the intermittent sampling of arterial and renal venous blood (and urine collection) together with continuous measurement (24 h/d) of renal blood flow (RBF) and arterial BP in freely moving rats before and for 3 wk following an increase of salt diet. Glomerular filtration rates (GFR) were similarly obtained from other group of unanesthetized SD rats. These data enabled the first determination of solute mass balances (metabolic fluxes) of O_2_ and metabolic substances in unanesthetized unrestrained SD rats. In parallel studies, renal cortical and medullary tissue samples were obtained from other groups of rats fed a 0.4% NaCl (LS) and at days 7, 14, and 21 after switching to a 4.0% NaCl (HS) diet. In addition, targeted mRNA expression analysis was performed in isolated cortical tubular segments and glomeruli. The results of the study show that even normal SD rats undergo enormous shifts in transcriptomic and metabolomic profiles in response to eating an HS diet. These adaptations appear necessary to sustain vital physiological functions of the kidney and to prevent injury in face a great increase of the metabolic workload placed upon the kidney when subjected to sustained HS diets.

## Methods

### Animals

Male SD rats were purchased from Envigo (Indianapolis, IN) and housed in environmentally controlled rooms with a 12-h light/dark cycle. Rats had free access to 0.4% NaCl AIN-76A diet (LS) (Dyets, Bethleham, PA) and water ad libitum. All protocols were approved by the Medical College of Wisconsin Institutional Animal Care and Use Committee (AUA00000851).

### Chronic RBF Measurement and Blood Sample Collection

Rats (*n* = 7, 10–11 wk of age) were performed RBF probe (Transonic, Ithaca, NY) implantation and femoral arterial catheterization as previously described.^24,25^ Briefly, rats were anesthetized with isoflurane and arterial catheter was inserted as previously described.^26–28^ Following an abdominal incision, RBF probe was implanted on left renal artery and the cable was exposed at nape of the neck via the subcutaneous route. In addition to the RBF probe implantation, renal venous catheter (MRE025, BRAINTREE, MA) was inserted through the femoral vein and placed in the left renal vein and secured to the luminal wall with 10–0 nylon (Figure S1). Three % heparinized saline was infused at a rate of 100 μL/h through arterial and renal venous catheter throughout the study. RBF and BP via arterial line were measured by conscious freely moving rats and recorded on average of every minute for 24 h/d. After 7–10 d of recovery period, 200 μL of arterial and renal venous blood were sampled and that blood was replaced from donor rats before and following 7, 14, and 21 d after the switch in diet from 0.4% (LS) to 4.0% (HS) salt diet. The 4.0% salt diet was chosen in this study because our previous studies have shown that it produces little change of blood pressure and kidney injury in SD rats or other “salt-resistant” strains of rats but does produce robust hypertension and kidney injury in Dahl SS rats in a few weeks.^29^ Blood gases (pO_2_ and pCO_2_; mmHg), electrolytes, total hemoglobin concentration (Hb; g/dL), and oxyhemoglobin saturation (SHbO_2_; %) were immediately measured by radiometer (ABL800 FLEX, Brea, CA). Overnight urine (18 h) from the day before the blood draw was collected on ice. The kidneys were collected either at 14 d of HS (HS14) or 21 d of HS (HS21). The kidneys of only LS fed SD rats were also collected for comparison. The collected kidneys (*n* = 5 for each group for metabolomics and mRNAseq analysis) were dissected to cortex and outer medulla and snap frozen with liquid nitrogen. Plasma, urine, and tissue were stored in −80°C until further analysis.

RBF in rats is often normalized by kidney weight, but it is impossible to repeatedly measure the kidney weight of the same rats and even measuring body weight repeatedly is difficult in this model. As salt did not alter the kidney weight of surgical sham control rats (*n* = 5 for LS group, *n* = 6 for HS group) (Table S1), data normalization to body weight was performed.

Since glomerular filtration rate (GFR) experiments and blood draws were performed during the daytime, the average RBF over a 12-h period during the daytime (6 am–6 pm) was used for the following calculations.

### Chronic GFR Measurement

GFR was measured by separate group of rats (*n* = 6) by transcutaneous measurement of FITC-sinistrin as previously described.^24,30,31^ Briefly, an indwelling inferior vena cava catheter was implanted 7–10 d before GFR measurement via femoral vein. An abdominal median incision was performed to be considered as a surgical sham of the other group. GFR was measured before and following 7, 14, and 21 d after the switch in diet from LS to HS. GFR (mL/min/100 g body wt) was defined as 21.33 mL/100 g body wt, the conversion factor calculated by Friedemann et al.,^32^ divided by FITC-sinistrin half-life (min). From the GFR and RBF from the other group, filtration fraction was calculated by the formula below:

*Filtration fraction = GFR/(2 × RBF × (1-Hct))*, where Hct is the hematocrit.

Tubular reabsorption of sodium was estimated as below^33^:

*GFR × whole blood Na^+^ (measured in RBF group rats by radiometer)—Urine flow × urinary Na^+^ (measured in RBF group rats by radiometer)*.

### Metabolomics Analysis

#### Plasma/Urine Metabolite Extraction

Metabolites were extracted from 20 μL of plasma and 20 μL of urine from each SD rat in the study according to standard operating procedures in the Mass Spectrometry and Protein Chemistry Service at The Jackson Laboratory.^34^ Metabolites were extracted using 500 μL of an ice cold 2:2:1 methanol:acetonitrile:water (MeOH:ACN:H_2_O) buffer; the sample was part of the water fraction. Caffeine, 1-napthylamine, and 9-anthracene carboxylic acid were all added at 0.5 ng/μL in the extraction buffer as internal standards. Each sample was then vortexed for 30 s on the highest setting, subject to 1 min of mixing with the Tissue Lyser II in prechilled cassettes, and then sonicated at 30 Hz for 5 min of 30 s on 30 s off in an ice water bath. Samples were then placed in the −20°C freezer overnight (16 h) for extraction. Following the extraction, samples were centrifuged at 21 000 × *g* at 4°C and supernatant from each metabolite extract was equally divided into five 2 mL microcentrifuge tubes. Each sample supernatant was divided into 5 equal volume aliquots, one for each of the 4 modes and the rest to create equal representation pools of all samples, one for each mode. Each aliquot was then dried down using a vacuum centrifuge for storage at −80°C until further use.

#### Tissue Metabolite Extraction

Metabolites were extracted from 20 mg of kidney cortex and medulla from each SD rat in the study according to standard operating procedures in the Mass Spectrometry and Protein Chemistry Service at The Jackson Laboratory^34^ as described for the plasma and urine samples with slight modification. Metabolites were extracted using 1000 μL of an ice cold 2:2:1 methanol:acetonitrile:water (MeOH:ACN:H_2_O) buffer containing internal standards as above per 20 mg of sample to ensure the extraction equivalents were normalized. Each sample had a 5-mm stainless steel bead added, then were pulverized in extraction buffer for 2 min using Tissue Lyser II. Samples were then placed in the −20°C freezer overnight (16 h) for extraction and the supernatant was collected as with the urine/plasma samples. Each sample supernatant was divided into 5 equal volume aliquots, one for each of the 4 modes and the rest to create equal representation pools of all samples, one for each mode. Each aliquot was then dried down using a vacuum centrifuge for storage at −80°C until further use.

#### Discovery Metabolomics Analysis

The Mass Spectrometry and Protein Chemistry Service at The Jackson Laboratory performed 4 mode tandem MS2 metabolomics analysis using a Thermo Q-Exactive Orbitrap mass spectrometer coupled to a dual-channel Vanquish Ultra-Performance Liquid Chromatography system as described previously.^34^ All samples were subject to the 4 modes of analysis consisting of a 25-min gradient over a hydrophobic C18 column (Agilent InfinityLab Poroshell 120 EC-C18, #699775–902T) and a hydrophilic HILIC column (Agilent InfinityLab Poroshell 120 HILIC-Z, #689775–924) column in positive and negative polarity. The C18 runs used a gradient from 99.8% H_2_O with 0.2% acetic acid (Solvent A1) to 99.8% ACN with 0.2% acetic acid (Solvent B1). The specific C18 gradient consisted of the following steps: 0–1 min at 98% A1/2% B1, 1–13 min from 98% A1/2% B1 to 10% A1/90% B1, 13–15 min at 10% A1/90% B1, 15–16 min from 10% A1/90% B1 to 98% A1/2% B1, and was re-equilibrated from 16–25 min at 98% A1/2% B1. All HILIC positive runs used a gradient from 10 m m ammonium formate in H_2_O with 0.1% formic acid (Solvent A2) to 90% ACN with 10 m m ammonium formate in H_2_O with 0.1% formic acid (Solvent B2). HILIC negative runs utilized a gradient of 10 m m ammonium acetate in H_2_O, pH 9.0 with 0.1% AffinityLab Deactivator Inhibitor (Agilent, #5191–3940; Solvent A3) to 85% ACN with 10 m m ammonium acetate in H_2_O with 0.1% AffinityLab Deactivator Inhibitor (Solvent B3). The 25-min gradient for the HILIC modes consisted of the following steps (A/B refer to A2/A3 B2/B3 for the respective HILIC mode): 0–1 min at 2% A/98% B, 1–11 min from 2% A/98% B to 30% A/70% B, 11–12 min from 30% A/70% B to 40% A/60% B, 12–16 min from 40% A/60% B to 95% A/5% B, was held at 95% A/5% B from 16–18 min, 18–20 min from 95% A/5% B to 2% A/98% B, and was re-equilibrated from 20–25 min at 2% A/98% B.

Each sample was reconstituted in 25 μL of 95% H_2_O/5% ACN for C18 modes and 95% ACN/5% H_2_O for HILIC modes. The sample run sequence was randomized (Random.org) and 2 technical replicates for each sample were injected at 10 μL (represents ∼2 μL of fluid samples or 2 mg of tissue sample starting volume weight, respectively, per run). Quality control pooled samples representing all samples within the specific fluid/tissue were run at the beginning and end of the run set at concentrations equivalent to the samples. These pooled samples were used for normalization through a quality control batch correction of the runs over time to account for technical variance. All instrument settings were set as described in a previous study.^34^

#### Metabolomics Data Analysis

The RAW data files (consisting of MS1 and MS2 spectra collected) were analyzed using Thermo Compound Discoverer (v3.2.0.421) according to supplementary methods from a previous study at The Jackson Laboratory.^34^ Spectra in the data were subject to a blank background subtraction to remove contaminant peaks (S/N threshold = 2). Additionally, all data were subject to a quality control correction selecting for peaks only consistently detected in the pool for normalization. The MS1 and MS2 data were searched against the Thermo mzCloud database, ChemSpider database, Metabolika Pathways, and mzLogic predicted composition in the Compound Discoverer workflow. In this workflow, the data were compared against standard databases containing MS2 spectra for high-confidence matching, where only those with MS2 matches in the database passed filtering. All data were then filtered for quality of MS2 spectral matching using an MS2 FISH coverage filter ≥ 10 in Compound Discoverer. This filtering allows for higher confidence identification and minimizes the false identification of metabolites. Additionally, metabolites that were focused on as key targets (eg, metabolites in the mass balance analysis) were checked for spectra matching beyond just FISH scoring as in Figure S2. Differential comparisons were performed comparing normalized abundances and *P*-values were calculated using the Tukey HSD test (posthoc) after an ANOVA test. From there the *P*-values were adjusted for stringency in the multiple testing using the Benajmini–Hochberg algorithm. The data also were run through The Jackson Laboratory in-house MetID Conversion tool created in R by the Computational Sciences Service to add additional identification numbers and metadata making it easier for other researchers to convert the nomenclature of metabolites used in this study in the future. These data are provided in the Supplemental File. Further analysis was performed using a combination of Compound Discoverer, custom R analysis, and MetaboAnalyst as needed.

### Lactate Assay

Tissue and plasma lactate concentration was validated by a commercially available kit according to the manufacturer’s instructions (PicoProbe Lactate Fluorometric Assay Kit, Bio Vision Cat# K638-100).

### mRNAseq Analysis

RNA was extracted from snap frozen tissues by Trizol reagent and cleaned up by RNAeasy MinElute Cleanup kit (Qiagen). RNAseq analysis and data analysis were performed at Novogene (Durham, NC). Detailed methods, quality control (Table S2), mapping results (Tables S3 and S4), and used software (Table S5) are shown in the supplement.

### Isolation of Nephron Segments and Quantification of RNA Expression Levels

The methods of the nephron segments isolation and qPCR were previously described^35,36^ and summarized in supplement. Primer information is shown in Table S6.

### Determination of Kidney O_2_ and Metabolites Extraction Ratio

Whole blood O_2_ extraction was calculated as previously described^37^:

*Oxygen content (mL/dL) = (1.31 × Hb (g/dL) × SHbO_2_) + (0.003 × pO_2_)*

*Oxygen delivery (DO_2_) (mL/min) per kidney = RBF (12 h daytime; mL/min) × arterial oxygen content (mL/mL)*

*Oxygen consumption (VO_2_) (mL/min) per kidney = RBF (12 h daytime) × arterial–renal venous content difference*

*Oxygen extraction ratio = VO_2_/DO_2_*

Metabolites solute mass balance^38^ was calculated similarly. Plasma flow [RBF × (1-Hct)] was doubled assuming equal in both kidneys.

*Metabolites* solute mass balance *= (2 × RBF × (1-Hct) × arterial–renal venous metabolites difference—urine flow × urinary metabolites)/(2 × RBF × (1-Hct) × arterial metabolites)*

#### Data Analysis of Metabolomics

Compound names were converted to the corresponding names available in the Metaboanalyst 5.0 database (https://www.metaboanalyst.ca) (analyzed September–December 2022) by “Compound ID Conversion” and those compounds are used as “reference metabolome,” which can be detected based on our analytical platform. Sparse partial least squares discriminant analysis (sPLS-DA) for all named compounds in each of the 4 modes (C18+/− and HILIC+/−) was performed. The number of components was fixed at 5 and variables per component at 20. Heat map with the hierarchical clustering analysis was performed for significantly changed compounds between LS, HS7, HS14, and HS21 (ANOVA Fisher’s LSD *P* < .05) with a Euclidean distance measure and by the Ward algorithm. Enrichment analysis for tissue metabolites was performed for significant differences by *t*-test (*P* < .05) in each of HS14 and HS21 compared to LS. Enrichment analysis for plasma metabolites was performed for significant different metabolites by linear models with covariate adjustments (*P* < .05) in arterial and venous differences. Those data analyses of metabolomics were performed using Metaboanalyst.^39^

#### Statistical Analysis

Continuous values are presented as the means ± SEM. Statistical comparisons were made using a *t*-test for 2-group comparisons, and analysis of variance (ANOVA) followed by Holm Sidak’s posthoc test for multiple between-group comparisons. A *P* < .05 was considered significant. ROUT test was performed for outlier test (*Q* = 5%). The error in the values obtained by combining values in tubular reabsorption of Na^+^ calculation with errors was indirectly estimated based on the error propagation formula shown below (*M*: Mean, *e*: error)^40^:


\begin{eqnarray*}
&&\left( {{M}_1 \pm {\mathrm{ }}{e}_1} \right){\mathrm{ }} \pm {\mathrm{ }}\left( {{M}_2 \pm {\mathrm{ }}{e}_2} \right) = \left( {{M}_1 \pm {\mathrm{ }}{M}_2} \right){\mathrm{ }} \pm {\mathrm{ }}{\left( {{e}_1^2 + {\mathrm{ }}{e}_2^2} \right)}^{1/2},\\
&&\left( {{M}_1 \pm {\mathrm{ }}{e}_1} \right){\mathrm{ }}\times{\mathrm{ }}\left( {{M}_2 \pm {\mathrm{ }}{e}_2} \right){\mathrm{ }} = {\mathrm{ }}\left( {{M}_1\times{\mathrm{ }}{M}_2} \right){\mathrm{ }} \pm {\mathrm{ }}{({\left( {{M}_2\times{\mathrm{ }}{e}_1} \right)}^2 + {\mathrm{ }}{\left( {{M}_1\times{\mathrm{ }}{e}_2} \right)}^2)}^{1/2},\\
&&\left( {{M}_1 \pm {\mathrm{ }}{e}_1} \right){\mathrm{ }}/{\mathrm{ }}\left( {{M}_2 \pm {\mathrm{ }}{e}_2} \right){\mathrm{ }} = {\mathrm{ }}{M}_1/{\mathrm{ }}{M}_2 \pm {\mathrm{ }}{({\left( {{e}_1/{M}_2} \right)}^2 + {\mathrm{ }}{\left( {{M}_1/{M}_2^2\times{\mathrm{ }}{e}_2} \right)}^2)}^{1/2}.
\end{eqnarray*}

## Results

### Effects of HS Diet on Arterial Pressure, RBF, and O_2_ Extraction.

**Figure 1.**
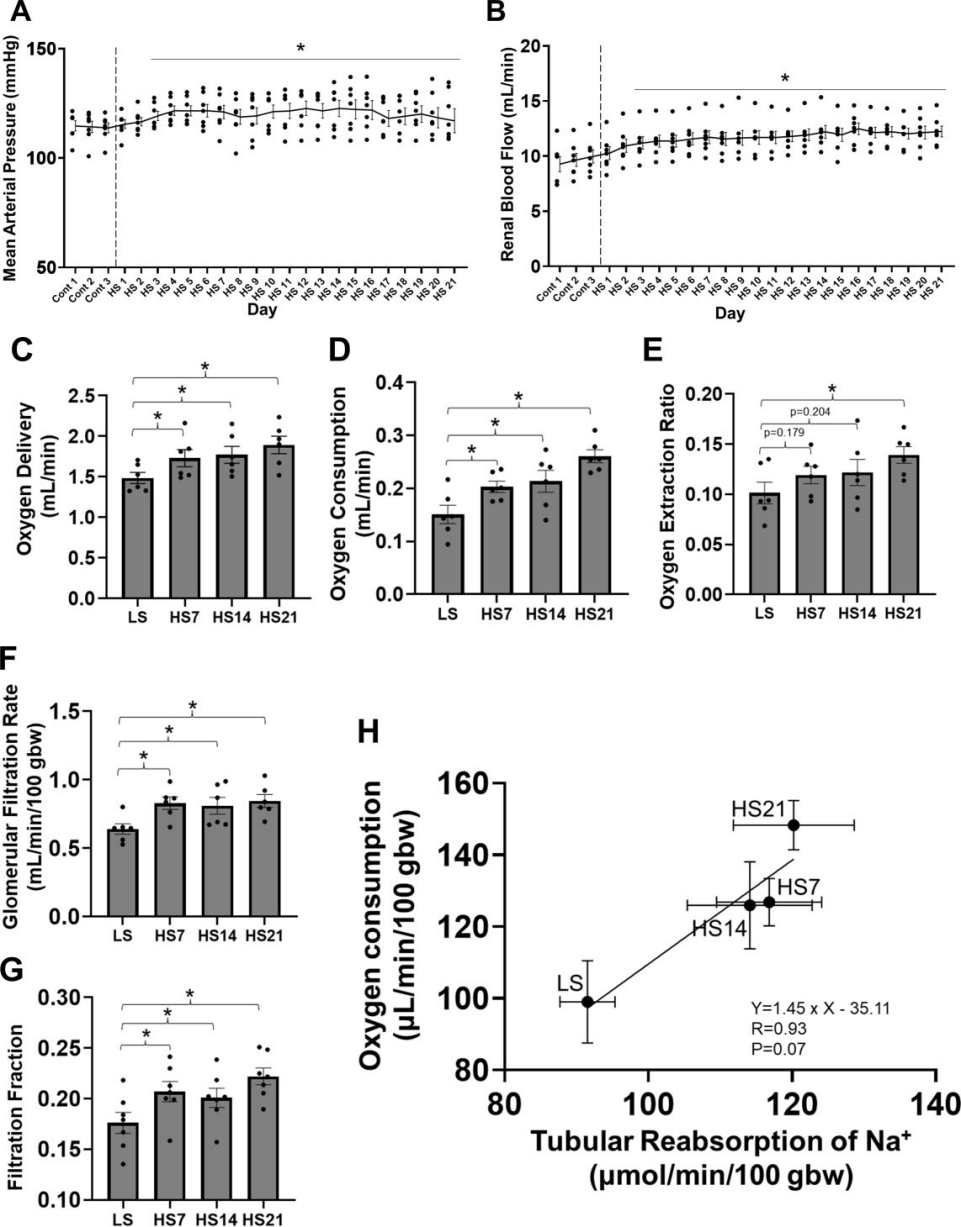
Physiological responses to the 4.0% NaCl diet. (A) Twenty-four hour/day average mean arterial pressure (MAP), (B) renal blood flow (RBF), (C) oxygen delivery, (D) oxygen consumption, and (E) oxygen extraction ratio obtained from same group of SD rats (*n* = 7). (F) Glomerular filtration rate (GFR) measured in a separate group of rats (*n* = 6), (G) Filtration fraction. (H) Correlation between O_2_ consumption and tubular reabsorption of Na^+^ determine using data from the latter two groups of rats. RBF and O_2_ data for single kidney were doubled for (G) and (H) to represent whole body values. One-way repeated measures (RM) ANOVA, Holm–Sidak for (A)–(G) and Pearson’s *r* correlation coefficient (H) were performed. Mean ± SEM and individual data. *P < .05 vs Cont 3 (A), (B) or LS (C)–(G). LS: low (0.4%) salt, HS: high (4%) salt.

Average 24 h mean arterial pressure (MAP) of SD rats slightly but significantly increased from 114 ± 2 to 119 ± 2 mmHg (*P* < .05) in 3 d after switching the diet from LS to HS and maintained at that level throughout the study (Figure 1A). Average 24 h RBF rose nearly 20% during the first 3 d from 10.0 ± 0.6 to 11.6 ± 0.6 mL/min (*P* < .05; Figure 1B) and was sustained at this elevated level throughout the 21 d of the HS diet. Normalized by the body weight determined in the surgical sham control rats (Table S7), the increase in RBF with the HS diet remained statistically significant at HS7, HS14, and HS21 (Table S8). Renal vascular resistance (RVR) did not change by HS (*P* = .75). An example of an SD rat in which arterial pressure and RBF were recorded continuously (24/7) before and 21 d following the switch to the HS diet is illustrated in Figure S3. A similar rise of RBF was observed in every rat studied and a similar increase in the magnitude of the diurnal rhythm of the RBF was observed in all rats. Shown in Figure S4, total urinary Na^+^ excretion increased 10-times (from 0.9 ± 0.1 to 10.3 ± 0.4 μmol/min) consistent with the 10-times increase in the % of NaCl in the diet when switch from 0.4% salt to 4.0% NaCl.

With the increase of RBF, the O_2_ delivery to the left kidney increased proportionally to RBF due to the unchanged O_2_ content in artery (Table S9) and remained elevated throughout the 21 d of the HS diet (Figure 1C). Both the calculated O_2_ consumption (Figure 1D) and extraction ratio (Figure 1E) rose progressively throughout the 21 d of the HS diet with the ratio increasing from 0.10 ± 0.01 to 0.14 ± 0.01 (*P* < .05) by HS21. This trend was maintained when normalized by body weight (O_2_ delivery: *P* = .186; O_2_ consumption: *P* < .05) (Table S8).

### Effects of HS Diet on GFR and Calculated Filtration Fraction.

In a separate group of rats, the total body GFR was determined in unanesthetized rats, which underwent the same surgery (sham) as those with implanted renal flow probes and subjected to the same dietary protocol. As summarized in Table S7 and Figure 1F, GFR increased from 0.64 ± 0.04 at LS to 0.83 ± 0.05 at HS7 (mL/min/100 g body weight) (*P* < .05) and was thereafter maintained at that level throughout the study. The filtration fraction (Figure 1G), calculated using the RBF determined from the continuously recorded rat group, was increased from 0.18 ± 0.01 at LS to 0.22 ± 0.02 at HS7 and remained elevated at this level throughout the study. As determined from these data, a strong positive correlation was found between total tubular reabsorption of Na^+^ and O_2_ consumption as presented in Figure 1H.

### Effects of HS Diet on Untargeted Metabolomic Profiles of Cortical and Outer Medullary Tissue

Figure S5A summarizes the total number of compounds detected in the renal cortical tissue (Cx) (5959) and in the outer medullary tissue (OM) (5785) combining the results detected in all modes. As illustrated, of the 5959 compounds in the Cx and 5785 in the OM, 2851 and 2620 are named compounds by Thermo Compound Discoverer (Figure S5B). Of all named compounds, 968 in Cx and 861 in OM were found in the Metaboanalyst 5.0 database (Figure S5B). Comparing LS values to those obtained after 14 d of the HS diet (HS14), it was found that 284 metabolites were significantly changed (raw *P* < .05) in the Cx and an equal number (284) significantly changed in the OM. Comparing LS values to those obtained after 21 d of the HS diet (HS21), 438 were significantly changed in the Cx and 349 in the OM (raw *P* < .05) (Figure S5B). *P*-value adjustments (Benjamini–Hochberg) reduced the chance of making Type-I errors and it was found that 138 named metabolites were significantly changed at HS14 compared to LS (adjusted *P* < .05) in Cx and 229 in the OM. At HS21, 218 were significantly changed in the Cx and 225 in the OM.

sPLS-DA was carried out for these named compounds to visualize and assess similarities and differences of metabolites in response to the HS diet. For each of the 4 modes (C18+/− and HILIC+/−), 5 components with 20 variables per rat were analyzed. As shown in Figure S6A, the analysis revealed that separate metabolic states were distinguishable within the Cx and OM tissues in response to the HS diet at both days 14 and 21. A clear distinction is observed between LS and HS14 and HS21 days of feeding.

The significantly altered metabolites over time (ANOVA Fisher’s LSD *P* < .05) determined in the C18+ mode were 321 of 1114 in Cx and 151/668 in OM. The significantly altered metabolites in the C18− mode were Cx 161/649 and OM 225/804; in the HILIC+ mode were Cx 241/565 and OM 129/805; and in the HILIC− mode were Cx 63/564 and OM 91/470. The heat maps of those metabolites are shown in Figure S6B in which the distinctive patterns observed by sPLS-DA were validated in the hierarchical clustering, which was performed with a Euclidean distance measure and by the Ward algorithm.

A metabolite enrichment analysis was performed for those metabolites that were significantly changed (*P* < .05) to prioritize and place them into known biological pathways as described by the small molecule pathway database (SMPDB). As shown in Figure S7, the “arachidonic acid metabolism” was significantly enriched in the Cx at HS14. In the OM, the “tyrosine metabolism,” the “lysine degradation,” and the “beta alanine metabolism” were significantly enriched at HS14. Notably, at HS21, no enrichment of any of the metabolomic pathways was found either in the Cx or in the OM.

### Effects of HS Upon Cortical and Outer Medullary mRNA Expression (mRNAseq Analysis)

mRNAseq analysis was performed on the same tissue analyzed for metabolites as described above to validate identification of enriched metabolomic pathways using a denser genomic scale dataset (eg, ∼3000 named metabolites vs ∼30 000 gene transcripts). There were 32 545 genes identified by the mRNAseq analysis, 22 293 were protein coding genes. Within that, adjusted *P* < .05 by Benjamini and Hochberg’s test, comparing LS to HS14, 497 significantly increased and 422 decreased in Cx. Comparing LS to HS21, 3044 increased and 2917 decreased in Cx. In the OM, comparing LS to HS14, 91 increased and 22 decreased and comparing LS to HS21, 555 increased and 165 decreased.

The genes that stand out among those that were significantly changed by the HS diet are those related to the inflammatory system [ie, C3 (log_2_ HS21/LS = 2.47), RT1-Db1(2.32), Itgal (2.32), etc]. In addition to these, in Cx the *Mthfr* (−2.27) gene was significantly changed whose polymorphisms are related to hypertension,^41^*Nrep* (−2.24) also known as *P311* stimulates translation of TGFβ and is related to tissue fibrosis,^42^ and *Slc16a1* (−2.16) also known as *Mct2* is a proton-linked monocarboxylate transporter.^43^ Immune system-related genes were also upregulated in OM.

**Figure 2.**
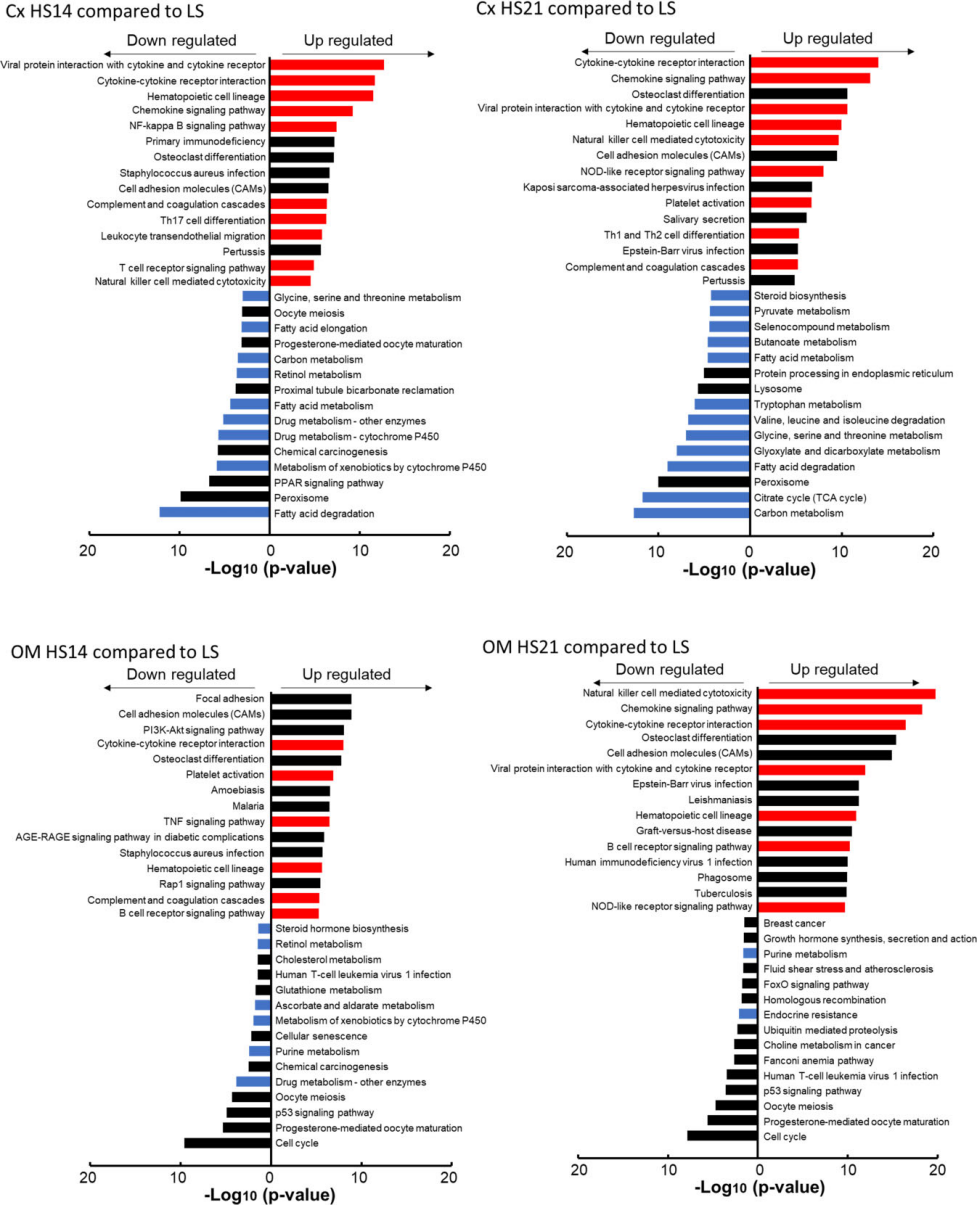
Results of the pathway analysis of the mRNAseq data showing the top 15 KEGG pathways (in order of −log_10_*P*-values) that were either significantly increased (“up regulated”) or significantly decreased (“down regulated”) in cortex (Cx) and outer medulla (OM) at either HS14 or HS21 compared to gene expression with the LS diet. Red bars denote “Signaling molecules and interaction pathway” and “Immune system pathway (KEGG map ID 04XXX).” Blue bars denote “Metabolism pathway (KEGG map ID 00XXX, 01XXX)” (August 2022).

The pathway analysis of mRNAseq data (KEGG) (Figure 2) found that genes most upregulated in the Cx tissue of SD rats fed HS diet were those related to the “Signaling molecules and interaction pathway,” the “Immune system pathway” (red bars), and related pathways including “Cytokines receptors,” “Chemokine signaling,” “NF-kappa B signaling,” “Th17 cell differentiation,” “T cell signaling,” etc. Those pathways most downregulated were those related to metabolism (blue bars) including “TCA cycle,” “Fatty acid degradation,” “Valine, Leucine, and Isoleucine degradation,” “Glycine, Serine, and Threonine metabolism,” “Carbon metabolism,” etc. In the OM tissue, the upregulated pathways were also largely related to the immune system, but interestingly fewer pathways of metabolism were found to be downregulated with the HS diet.

### mRNA Expression of Cortical Tubular Transporters

Of special mechanistic interest were the changes in gene expression of tubular transporters affected by the HS diet. Figure S8 shows the Cx tissue mRNA expression of genes encoding transporters for glucose and amino acids that tended to be downregulated at HS14 with most of these reaching statistical significance by HS21. This includes amino acid transporters (*Slc1, Slc3, Slc7*), sodium-glucose transporters (SGLT isoforms *Slc5a1, Slc5a2*), urate (*Slc22a12*), and lactate transporters (*Slc5a12, Slc5a8*). It was found that the glucose transporter 2 (GLUT isoforms *Slc2a2*) were downregulated at HS21 while expression of *Slc2a1* was increased. Although it is unclear which of these would predominate functionally, it is evident that the upregulation of the GLUT transporters would be consistent with a greater release of glucose and glycolysis products being released into the interstitial space. Also of note, monocarboxylate transporters (MCT) and Na^+^-K^+^-ATPases were upregulated by the HS diet, which is consistent with enhanced proximal tubule (PT) Na^+^ reabsorption necessitating greater utilization of ATP for active transport. Sodium transporters and channels including NHE3 (*Slc9a3*), NKCC2 (*Slc12a1*), NCC (*Slc12a3*), and ENaC (*Scnn1*) were upregulated. On the other hand, the effect of HS on the OM transporter genes was modest (Figure S9).

In addition, proteolysis-related genes are picked up in Figure S8 as well. There are myriads of proteolytic enzymes expressed in the kidney,^44,45^ and those are effected by HS diet in Cx tissue. Genes encoding protease-activated receptors (*F2r*)^46^ are upregulated by HS, whereas megalin (*Lrp2*) and clathrin (*Cltc*) were reduced. Interestingly, within cathepsin-encoding genes, *Ctsa* and *Ctsb*, which mainly expressed at PT S1 were downregulated and *Ctsc* and *Ctsd*, which mainly expressed at distal convoluted tubule (DCT) and connecting tubule (CNT) were upregulated.

### Omic Integration With Statistical Mapping and Validation of Metabolomic and mRNAseq Data

Data obtained from the metabolomics and mRNAseq analysis can be effectively utilized in several ways. First, to validate against each other the predicted pathways that appear to be most affecting metabolic functions when fed an HS diet. Second, to identify pathways that were not of obvious importance from the metabolomic analysis given the limited number of compounds that can currently be identified by a global mass spec analysis.

**Figure 3.**
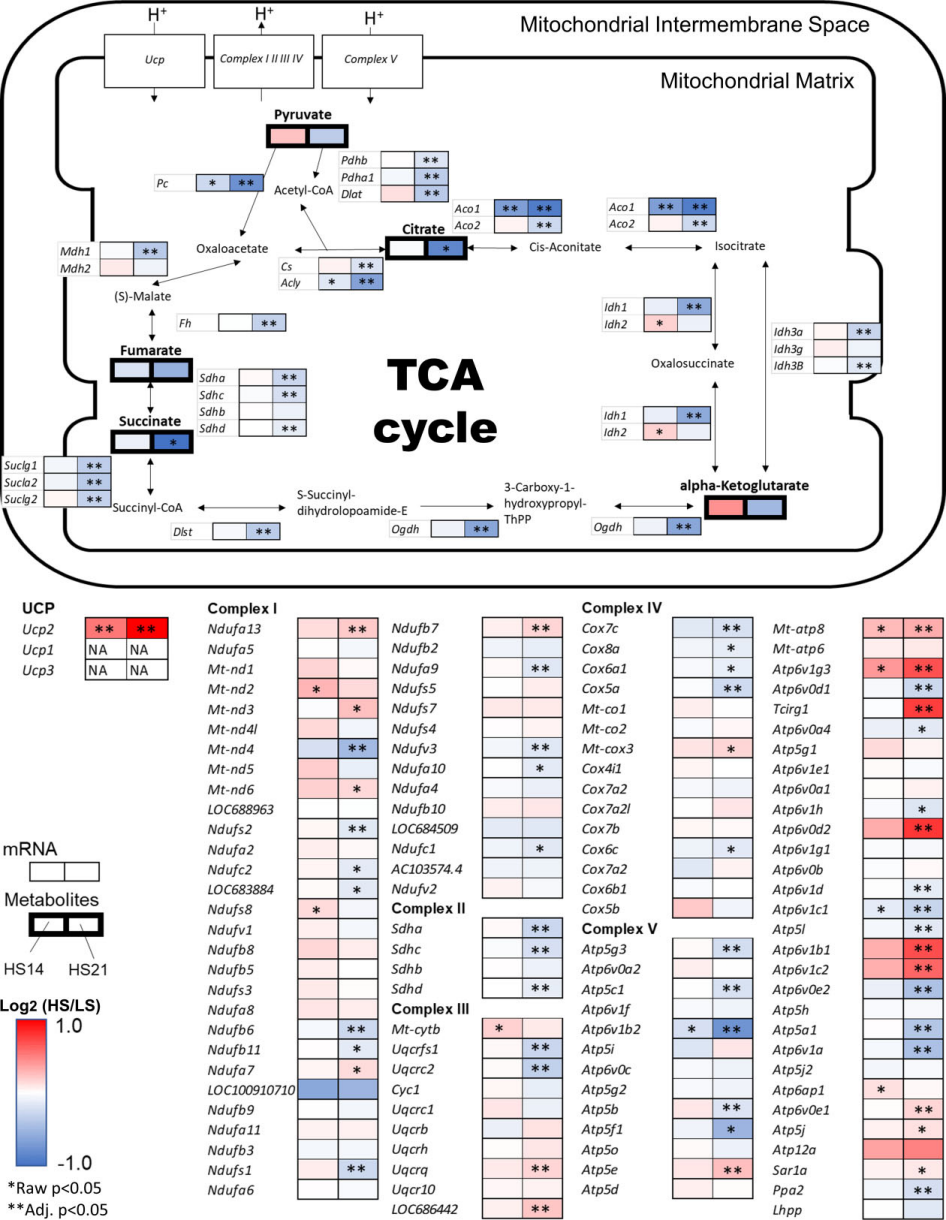
Integrated figure of TCA cycle in cortex the binomial logarithm of the ratio of high salt (HS) to low salt (LS) is represented in color. The left boxes are ratio of HS14 to LS and the right boxes are ratio of HS21 to LS. Thin boxes represent mRNA and thick boxes represent metabolites. Red denotes increase in expression and blue denotes decrease in expression. *Raw *P* < .05 in *t*-test for metabolomics and in DESeq2 for mRNAseq, **Adj. *P* < .05 in Benjamini and Hochberg. (KEGG map ID 00020, 00190 last update: 28 July 2022).

Figure 3 summarizes the integrated metabolomic and mRNA expression data related to mitochondrial energy production in the Cx sample. By HS21, both metabolites and genes encoding the major enzymes of the TCA cycle were found to be generally downregulated including reductions of citric acid, succinic acid, and fumarate. While mRNAs-encoding enzymes in the TCA cycle were downregulated overall, some of mRNAs-encoding proteins that make up the electron-transfer complex were not (Figure 3). Especially, many genes encoding for proteins of complex V including *Mt-atp8* were upregulated at HS21 while the expression of others was reduced. It was also found that the gene encoding uncoupling protein 2 (*Ucp2*) was upregulated by HS.

Several other important pathways were enriched in the metabolomic enrichment analysis (Figure S10). Specifically, the “Arachidonic acid metabolism” pathway in the Cx (Figure S10A) and the “Lysine degradation” pathway in the OM (Figure S10B) were altered by the HS diet. As shown in Figure S10A, the metabolomic analysis in the Cx found that arachidonic acid was significantly increased at HS14, together with downstream metabolites 8-HETE and thromboxane B2 (TXB2). The general upregulation of this pathway is reinforced by enhanced expression of most of the genes on arachidonic acid metabolism pathway, which were upregulated on HS14 and HS21 compared to expression observed with the LS diet. The notable exception to this were the *Cyp-4* genes important in the production of 20-HETE, which has both pro- and antihypertensive actions resulting from modulation of vascular and tubular functions of the kidney.^47^Figure S10B illustrates the “Lysine degradation” pathway in which many key elements were found in the metabolomic analysis to be downregulated in the OM as found at days HS14 and HS21. This included reduction of tissue lysine itself and reduced levels of α‐ketoglutarate, glutamate, and allysine. However, since lysine was significantly reduced, a reduction in the metabolites derived from lysine would be expected to be also reduced with no significant changes in the enzymes in this pathway were observed by the mRNAseq analysis.

### Effects of the HS Diet Upon Glycolysis in the Renal Cortex

**Figure 4.**
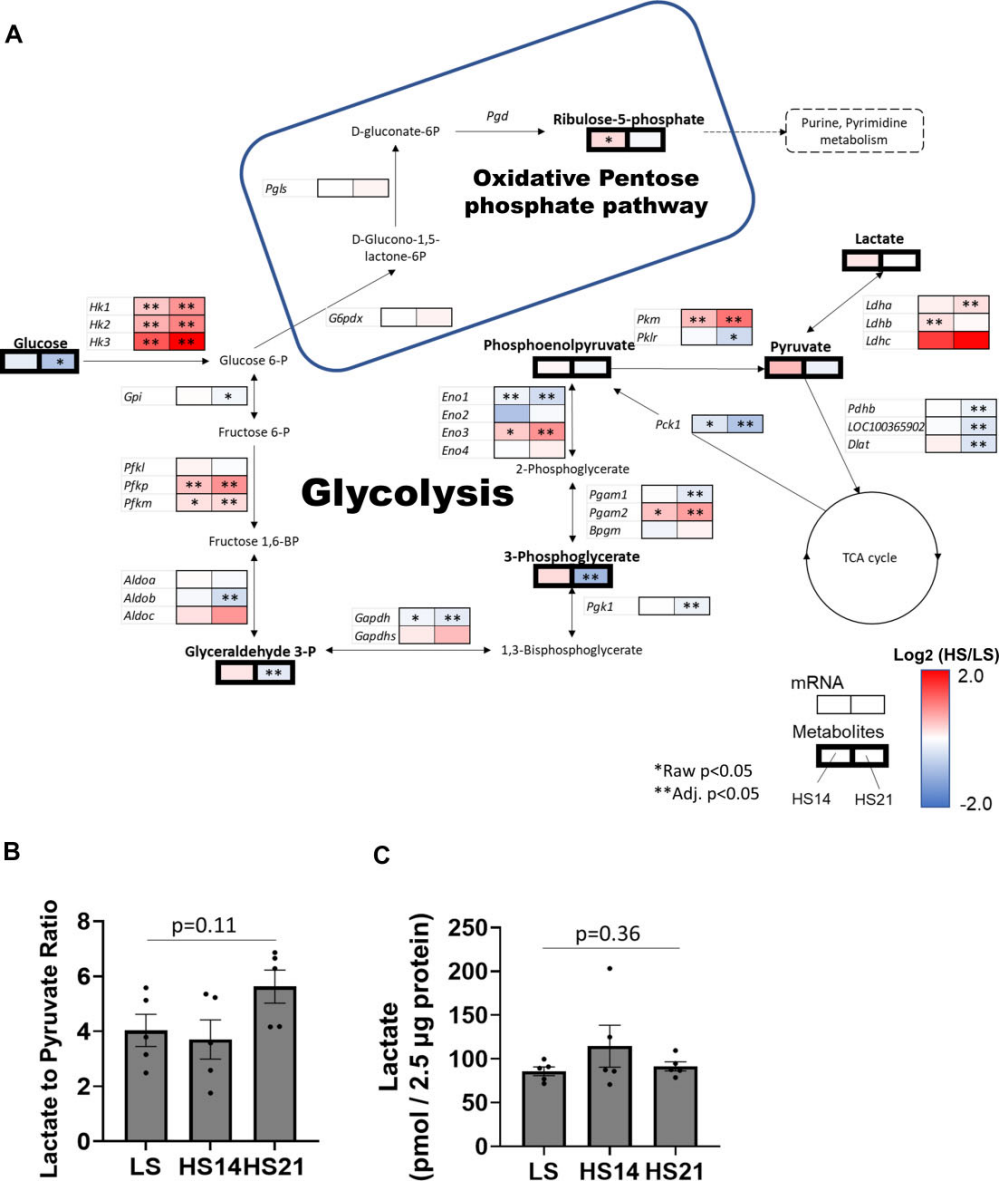
Integrated figure of glycolysis in cortex. (A) The binomial logarithm of the ratio of HS to LS are represented in color. The left boxes are ratio of HS14 to LS and the right boxes are ratio of HS21 to LS. Thin boxes represent mRNA and thick boxes represent metabolites. Red denotes increase in expression and blue denotes decrease in expression. The frame denotes oxidative pentose phosphate pathway. *Raw *P* < .05 in *t*-test for metabolomics and in DESeq2 for mRNAseq, **Adj. *P* < .05 in Benjamini and Hochberg. (KEGG map ID 00010 last update: 7 May, 2020, 00030 last update: 13 December, 2022). (B) Lactate to pyruvate ratio in cortical tissue in metabolomics. (C) Validation of lactate concentration in cortical tissue by fluorometric assay kit. Mean ± SEM and individual data. One-way ANOVA, No significant difference between groups.

As illustrated in Figure 4A, the Cx levels of glucose tended to be reduced by day 14 of the HS diet and were significantly reduced by day 21 of the HS diet. A seemingly compensatory response is reflected by the observation that many genes encoding glycolytic enzymes were upregulated at HS14 and even more so at HS21. Significant increases (*P* < .05) were found in hexokinase isoforms (*Hk*) required for conversion of glucose to glucose 6-P, and in phosphofructokinase isoforms (*Pfk*), required to convert glucose 6-P to fructose 1,6-BP. A reduction of glyceraldehyde 3-P and 3-phosphoglycerate was found at HS21 and several isoforms of aldo-keto reductase (*Aldo*) and glyeraldehyde-3-phosphate dehydrogenase (*Gapdh*) were found to be reduced at HS21. Increases were also found in pyruvate kinase (*Pkm*). The Cx pyruvate levels tended to be increased at HS14 but did not differ from levels observed with LS at HS21. Lactate dehydrogenases (*Ldh*) were also upregulated, but the tissue lactate did not differ from levels observed with LS at either HS14 or HS21.

The relationship of glycolysis with the oxidative pentose phosphate pathway (PPP) is also illustrated in Figure 4A. A moderate increase (*P* < .05) of ribose-5-phosphate was found at HS14 indicating a greater oxidation of glucose via this pathway which at this time would yield greater NADPH to scavenge ROS. However, at HS21 ribose-5-phosphate was no longer found to be elevated. The lactate to pyruvate ratio in cortical tissue tended to increase from 4.0 ± 0.6 at LS to 5.6 ± 0.6 at HS21 but did not reach statistical significance (*P* = .11) (Figure 4B). As activation of glycolysis was of particular interest, lactate concentration in the Cx tissue was validated by fluorescent lactate analysis, which showed the similar trend as metabolomics data (Figure 4C). Further arteriovenous solute mass balance analyses are discussed below.

In general, it appears that metabolites of the glycolytic pathway in the Cx may be initially elevated at HS14 but by HS21 appear to be reduced to levels similar to or less than that observed when fed the LS diet. As noted in Figure S8, gene expression of the PT apical membrane SGLT transporter *Scl5a1* isoform was increased at HS14 (*P* < .05) but not at HS21. A significant reduction of the *Scl5a2* isoform was found at HS21 suggesting there could be a reduced luminal uptake of glucose in the PTs, perhaps contributing to the reduced Cx glucose levels. Gluconeogenesis may be expected to be suppressed since phosphoenolpyruvate carboxykinase 1 (*Pck1*) that acts as the rate limiting enzyme in gluconeogenesis was reduced (Figure 4A).

Many genes involved in the glycolytic and TCA cycle were altered in Cx, while less significant changes were observed in OM (Figure S11).

Figure S12 summarizes the genes and metabolites related to the malate–aspartate shuttle. The mRNAseq analysis confirmed that this pathway exists in the Cx, but since gene expression was not assessed separately in the cytoplasm and mitochondria one cannot determine whether the expression of enzymes that directly supply protons, such as *Mdh*, were altered. Malate levels were reduced at HS21 (*P* < .05) as was *Slc25a11* gene expression, which codes for the mitochondrial carrier that transports malate across the IMM. It is interesting that *Slc25a12*, which codes for the protein that transports aspartate across the IMM to the intermembrane space was significantly increased at HS21, but the relevance of this is unclear.

Figure S13 summarizes the genes and metabolites related to urea cycle and nitric oxide production in the renal Cx. It was found in the Cx that citrulline and arginine were significantly reduced together with a reduction in *Nos1* mRNA expression at HS14. Aspartate was found to be elevated perhaps representing a compensatory response. By HS21, arginine had returned to levels observed with LS and was associated with elevations of *Nos3*, which appears to represent a compensatory response. *Arg2* mRNA expression was also elevated at HS21, which could also drive an increase of urea production although urea was not measured by the mass spec analysis.

### Targeted mRNA Expression Analysis of Isolated Cortical Tubular Segments and Glomeruli

**Figure 5.**
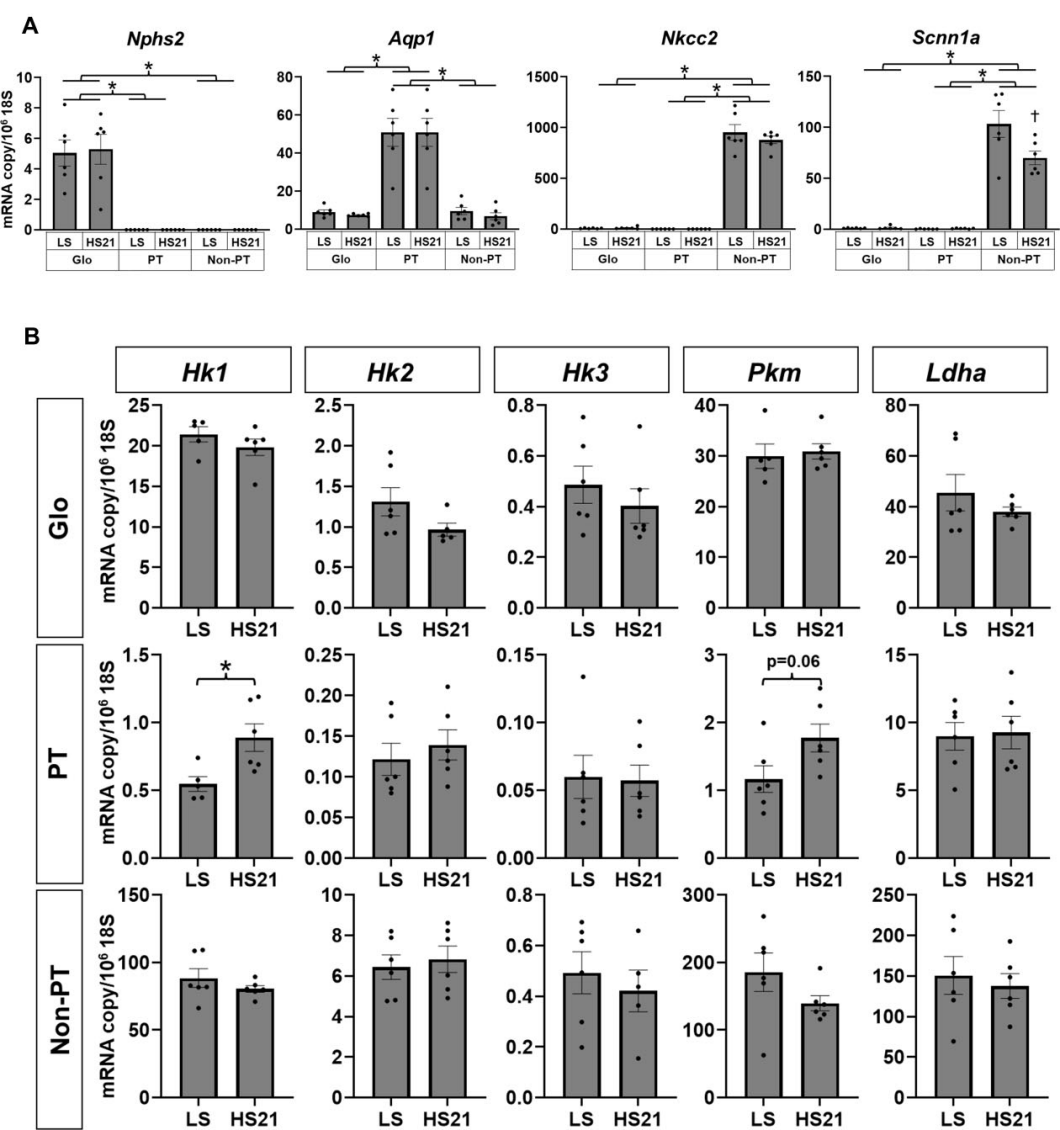
mRNA expression of isolated PTs. (A) mRNA expression of *Nphs2, Aqp1, Nkcc2*, and *Scnn1a* in isolated glomeruli (Glo), proximal tubules (PT), and nonproximal cortical tubules (non-PT) of low salt group (LS) and 21 d of high salt group (HS21) are shown. Mean ± SEM and individual data. *P < .05 in two-way RM ANOVA, Holm–Sidak. (B) mRNA expressions of *Hk1, Hk2, Hk3, Pkm*, and *Ldha* in Glo, PT, and non-PT of LS and HS21 are shown. *P < .05 in *t*-test.

The purity of isolated tubules was determined by comparing expression differences of *Nphs2* (podicin in glomeruli), *Apq1* (aquaporin I in PT), *Nkcc2* (*Slc12a1*) (Na^+^/K^+^/2Cl^−^ co-transporter in cTAL), and *Scnn1a* (alpha subunit of ENaC in cortical collecting duct). As shown in Figure 5A, *Nphs2* could be detected only in the isolated glomeruli, whereas it was scarcely detected in isolated PT, which overwhelmingly expressed *Aqp1. Nkcc2* and *Scnn1a* were clearly expressed both in the cTAL and cortical collecting duct non-PT segments but were absent in PT and glomeruli. There was no difference between LS and HS21 in any tissue, except that *Scnn1a* was decreased in non-PT HS21. Since we were unable to cleanly distinguish cTAL segments from cortical collecting duct segments, these were analyzed together and designated as non-PT segments. Consistent with the mRNAseq analysis of cortical tissue significant increases in expression of hexokinase 1 (*Hk1*) and *Pkm* (pyruvate kinase) were found in PT of HS fed rats consistent with an increases of activity of the glycolytic pathway (Figure 5B). Although *Hk1* expression levels are relatively low in the PT, the HS diet resulted in a significant increase while *Hk2* and *Hk3* expressed at much lower levels remained unchanged as was the case for *Ldha* (lactate dehydrogenase-A). No mRNA expression differences to the HS diet of *Hk1, Hk2, Hk3, Pkm*, or *Ldha* were observed in either the glomeruli or the nonproximal tubular segments (Figure 5B). Standard curves of *Hk1* and *Pkm* primers are shown in Figure S14A. All samples were within detection limits. Figure S14B shows an example of an amplification plot with the *Hk1* primer in glomerular samples and PT samples.

### Arterial and Venous Metabolites and Solute Mass Balance Determinations

As shown in Figure S15, a total of 3137 of compounds in plasma were detected when the detections from all 4 modes were combined. Of these,1531 are named compounds.

We first analyzed the metabolomic profiles in the artery (Art) and renal vein (RV) separately. It was found that the Art (Figure S16A) and RV metabolomes (Figure S16B) as analyzed by sPLS-DA exhibited a clear shift in the metabolic states at HS7 and HS14. It is also evident that by HS21, the metabolic state was more similar to that found when rats were fed the LS diet representing a return to “normal” although clear differences are seen in component 2 metabolites in both Art and RV samples. This return of the metabolomic profiles toward that of the LS state after 21 d of the HS diet is also clearly seen in the associated heat maps representing those metabolites that were significantly changed by the HS diet for each of the designated modes (ANOVA Fisher’s LSD *P* < .05). Specifically, the number of significantly altered metabolites from the total metabolites, which are detected in different modes are as follows: C18+ Art 96/808, C18+ RV 65/808, C18− Art 35/454, C18− RV 38/454, HILIC+ Art 31/387, HILIC+ RV 40/387, HILIC− Art 19/122, and HILIC− RV 15/122.

Then, the difference of metabolites between Art and RV was examined. The distribution of the ratio of RV to Art metabolites is shown in Figure S17. Focusing the analysis on the differences between the Art and RV of those named compounds in response to the HS diet it is seen in Figure S18A that by sPLS-DA analysis that separations were found between Art and Rv metabolites in the C18+ and HILIC+ modes comparing LS and HS7, HS14 and HS21. However, there was no clear separation found between the days of the HS diet. As illustrated in Figure S15B, within the named 1531 compounds, 546 compounds were contained in the Metaboanalyst 5.0 database. Of these, 131 compounds (Table S10) were significantly (raw *P* < .05) changed over time by HS as determined using linear models with covariate adjustments^48^ on Metaboanalyst 5.0. Enrichment analysis of those metabolites revealed the significant enrichment of “oxidation of branched chain fatty acids” and “carnitine synthesis” (Figure S18B) including the metabolites such as carnitine, acetylcarnitine, lysine, and α-ketoglutarate.

The urine metabolomic features are summarized in Figure S19 showing that a total of 10 241 compounds were identified in the urine sample from the four modes (C18+/− and HILIC+/−). Of these, 4980 were named compounds and after removing duplications and those compounds not found in plasma, there remained 749 compounds. Of these, only 367 were found to match the Metaboanalyst 5.0 database, which were used for the final solute mass balance analysis. Those metabolites in the urine for which the urine excretion exceeded the total kidney filtration fraction are shown in Table S11. With LS feeding, 19 metabolites were found to be excreted in excess of the filtration fraction. Interestingly, the number of metabolites excreted more than filtration fraction kept increasing over time (29 at HS7, 37 at HS14, and to 39 at HS21) and began to include uremic toxins such as creatinine, indoxyl sulfate, and hippuric acid. The clearances of those 367 metabolites were calculated and expressed in scatter plot in Figure S20. Carbohydrate and amino acids were specifically analyzed to determine changes over time, which were graphed as shown in Figure S21. It was found that most of the clearances of metabolites were increased by the HS.

**Figure 6.**
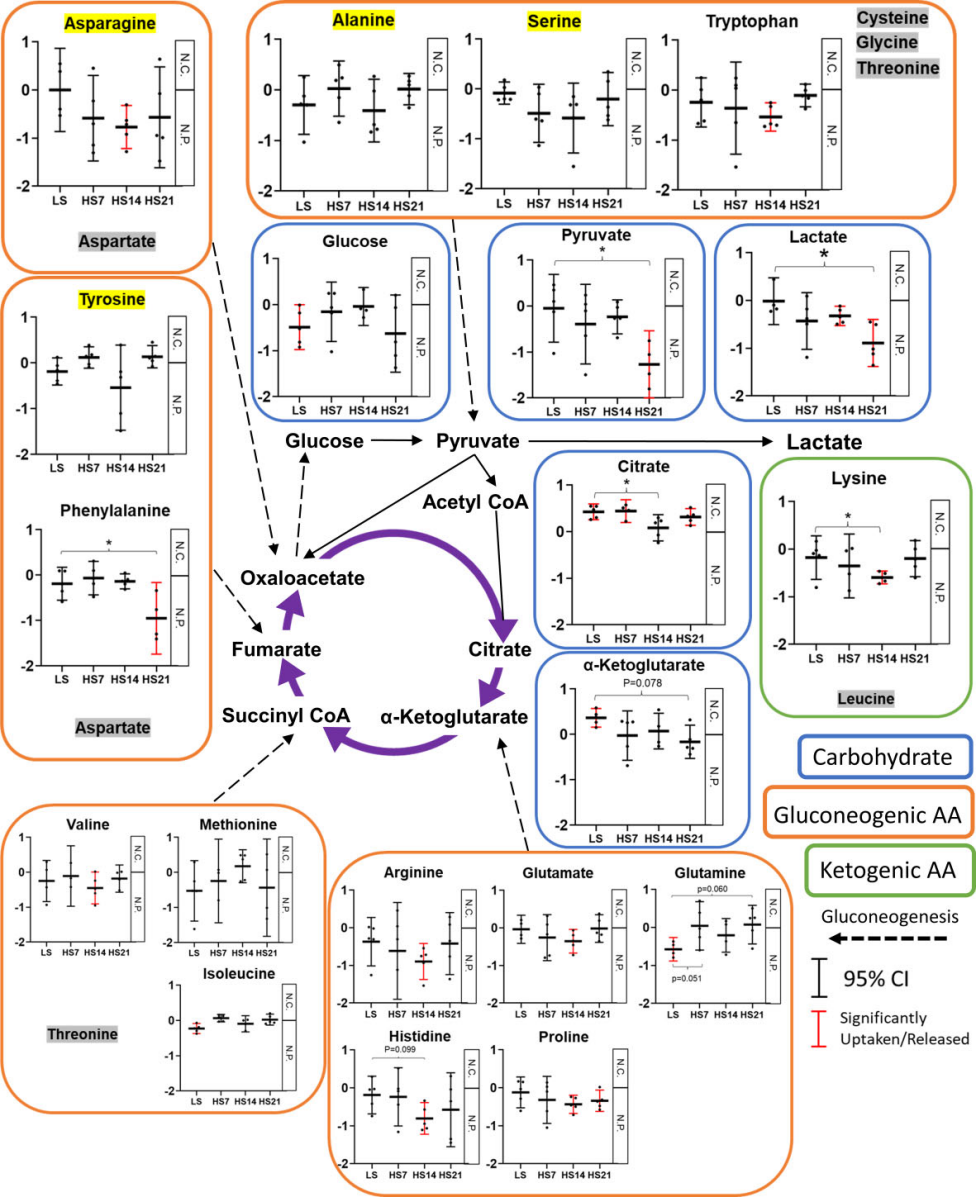
Calculated metabolomic solute mass balance mean ± 95% CI of mean and individual data are shown in each graph. Horizontal line is date, vertical line is solute mass balance (calculation written in methods. unitless). Red graph denotes the significantly net produced or net consumed in each time point. *P < .05 vs LS, one-way RM ANOVA, Holm–Sidak. Blue boxes: carbohydrates and their derivative, orange boxes: gluconeogenic amino acid (AA), green boxes: ketogenic AA. Purple arrows denote TCA cycle and dash arrows denote gluconeogenesis. Highlighted by yellow are metabolites, which are not detected in urine (ie, only arterial and venous plasma are used for solute mass balance calculation). Highlighted by gray are not detected in plasma. [Adapted the figure of Stryer Biochemistry. 7th edition (2012)^116^].

Calculated metabolite solute mass balances comparing those determined in rats fed LS to those at HS7, HS14, and HS21 are shown in Figure S22 and Figure 6. In these individual graphs in Figure 6, metabolite solute mass balances are represented from two perspectives. First, whether the metabolites were net consumed (N.C.) or net produced (N.P.). For this purpose, the 95% CI of the mean was calculated and if “0” was not contained within this confidence interval it indicates a compound is either net consumed or net produced by kidney (*P* < .05, highlighted in red in Figure 6). Second, the graphs reflect whether the solute mass balances changes over time, for which a one-way repeated measures ANOVA post hoc Holm–Sidak was performed comparing all times to the LS fed state (*P* < .05 indicated by *). The analysis indicates that the solute mass balance of some of the important carbohydrates and their derivative utilized for both glycolysis and the TCA cycle were modified by HS intake (see graphs in blue boxes). Specifically, it is seen that in the LS state glucose was being net produced in the kidney (*P* < .05), which was not observed during the days of HS feeding. Lactate net production became significant (*P* < .05) at days 14 and 21 of the HS diet with a similar trend in pyruvate. Plasma lactate concentration was validated by fluorescent assay kit and a similar tendency was observed (Table S12). There was a greater net consumption of the important TCA cycle intermediate α-ketoglutarate (*P* < .05) in the LS fed rats, which was no longer apparent with HS feeding. A net consumption of citrate was found with the LS diet and this positive net consumption was sustained throughout most of the periods of HS feeding (*P* < .05) except at HS14. Gluconeogenic amino acids (see graphs in orange boxes) including asparagine, tryptophan, phenylalanine, valine, arginine, glutamate, histidine, and proline were net produced in significantly greater amounts (*P* < .05) during various days of the HS diet. The ketogenic amino acid lysine was also net produced from the kidney at HS14 (see graph in green box).

## Discussion

The kidneys play a crucial role in eliminating excess salt in the diet and maintaining homeostasis in the body. In salt-sensitive individuals with reduced sodium excretory function BP increases when fed an HS diet, which in turn leads to vascular, cardiac and kidney dysfunction, and injury. Although excess salt intake is less likely to produce hypertension in individuals with LS sensitivity,^49–51^ the present study finds that an HS diet does have a significant effect on kidney metabolism even in normal SD rats in which minimal hypertension is observed when fed an HS diet.

### Effect of Excess Salt on Renal Hemodynamics and O_2_ Utilization in SD Rats

As determined by continuous 24 h/d monitoring, the SD rats as expected showed only a slight increase in the average daily MAP (∼5 mmHg) in response to the HS diet. By comparison, a relatively much larger increase in RBF was observed. An even greater increase of GFR was observed consistent with the previous observations^52^ although the mechanism for this are unclear. Nevertheless, this resulted in the significant increase in the calculated filtration fraction (FF). Although high GFR salt-sensitivity is thought to be associated with greater susceptibility to progression of renal dysfunction,^53–55^ it is clear that SD rats possess compensatory mechanisms that enable them to compensate and prevent the injurious effects of an HS diet. This is in stark contrast to Dahl SS rats, which were generated by selective breeding of SD rats and whose GFR is reduced by the second week of HS feeding.^30^ Increased FFs with HS intake have also been observed in salt-sensitive humans (increase in MAP by 8 mmHg, increase in FF by 0.04) and women using oral contraceptives (increase in MAP by 1–2 mmHg, increase in FF by 0.02).^56,57^

As O_2_ content in arterial blood did not change over time with the HS diet, O_2_ delivery increased in proportion to RBF. On the other hand, O_2_ consumption increased proportionally greater than the increase in the delivery. Although an HS diet was reported to decrease the tubular O_2_ consumption in isolated microdissected renal tubules from mice,^58^ this does not appear to reflect in vivo responses where tubular O_2_ consumption is altered by many factors including GFR and RBF that must be taken into account.^59^ Although the correlation between Na^+^ reabsorption and O_2_ consumption in the kidney is well recognized as determined under a variety of conditions,^33,60^ to the best of our knowledge, this is the first report that has evaluated this relationship in the unanesthetized freely moving animal repeatedly. In previous in vivo experiments with dogs, renal Na^+^ reabsorption was reported to be 20 mol per mol of O_2._^14,60^ Our data at LS are 20.7 Na/O_2_ (assuming 22.4 L/mol of O_2_), which is close to the previously reported value. It is noteworthy that the value of Na/O_2_ did not significantly change even under the HS (18.2 at HS21). Given that 99% of the filtered Na^+^ is reabsorbed, the tubular load is primarily dictated by increase in GFR so an increase of GFR would be expected to increase the tubular workload and O_2_ consumption.

Salt administration altered the expression of many gene-encoding cortical tubular Na^+^ transporters, which was especially evident at HS21. The enhanced expression of Na^+^ transporters in the cortex may have affected metabolic changes. It was found that Na^+^ transporters and channels were generally upregulated whereas sugar and amino acids transporters were found to be downregulated. As illustrated in Figure S8, in cortical tissue (Cx), increased gene expression was found of *Nkcc2* (*Slc12a1*), which is expressed in cTAL, NCC (*Slc12a3*), which is expressed in the aldosterone sensitive distal collecting tubules (DCT), ENaCa (*Scnn1a*), which is expressed in the DCT and cortical collecting ducts (CCD), and NHE3 (*Slc9a3*), which is expressed in PT.^61^ Together, the increased mRNA expressions of the transporters would be expected to increase both proximal and distal tubular Na^+^ reabsorption.^62,63^ These observations are consistent with the conclusions reached by Udwan et al.^58^ that the fractional reabsorption of Na^+^ is distributed differently along the tubule as determined by dietary Na^+^ intake.

### Change in Metabolites and Gene Expression by the HS Diet

Despite the current progress in mass spectrometry and the ability to detect more than 5000 metabolites within biological samples, the number of annotated compounds reduces that number to several thousand and of those the assignment to known biochemical pathways is a limiting factor when compared to those obtained from mRNAseq analysis in which more than 20 000 known protein-coding genes can be mapped to less than 1000 biochemical metabolites represented in the KEGG pathway maps. In the present study, we have utilized the combined strength of large-scale transcriptome sequencing (mRNAseq) with global profiling of metabolites in which the integrated analysis has identified many pathways of metabolism in which statistically significant differences to salt diet were obtained. Even those which did not reach statistically significant differences for the metabolomic analysis were of great utility when changes were consistent with pathways found of importance in the mRNAseq analysis.

### Compensatory Mechanisms to Protect From Hypertension and Kidney Injury in SD Rats

One of the important underlying questions is what are the underlying compensatory mechanisms that protect the SD rat from hypertension and kidney injury when fed an HS diet. Although the specific answer to this question is not provided by the present analysis, enormous changes are occurring in the kidney metabolism and in many of the molecular and biochemical pathways that affect major functions of the kidney and inflammatory pathways of tissue injury. Inflammatory pathways are stimulated by ROS which is generated during the process of oxidative phosphorylation. Several pathways work as ROS scavengers are found in this study. First, oxidative PPP might be upregulated, which is evident from increase in metabolites, Ribulose-5-phosphate. PPP generate NADPH, which is required for antioxidant system.^64^ Second, *Ucp2* mRNA expression elevated by the HS diet, which is also an ROS scavenger.^65^ Activation of *Nos3* mRNA expression was also found. NOS3 generate nitric oxide and work as an ROS scavenger.^66^ These might interact with each other to scavenge ROS and protect kidneys from damage.

Although the relationship between inflammation in the kidney and hypertension has been studied intensely over the last decade,^67–69^ the effect of salt on kidney without hypertension has been barely studied. It is interesting to note that the present study suggests that salt intake may activate the inflammatory system in the kidney, even if the increase in blood pressure is slight (∼5 mmHg). In the present study, the HS diet increased renal oxygen consumption, and the associated ROS generation may have contributed to the activation of NFkB and other inflammatory systems.^70^ Additionally, the observed increase in GFR in the current study suggests increases of tubular flow, as confirmed in microperfusion studies,^71,72^ which increase shear stress or hoop stress on tubules^73,74^ activates mTORC1^36^ thereby altering cellular metabolism. The mechanism of the inflammatory response independent of changes in blood pressure is an intriguing finding, and further studies are needed to elucidate the mechanism.

The results of this study show that the profile of metabolites both in the kidneys and in systemic (ie, arterial plasma) changed over time in response to the HS diet as it was found that considerable changes occurred in both arterial and renal venous blood metabolic profiles. It is very interesting, however, that although significant changes were observed in the plasma metabolic profiles at HS days 7 and 14, the general metabolic profile returned in each of these rats to one similar to that observed with the LS diet by HS day 21. This is in contrast to the profiles obtained from the Cx and OM tissue analysis, which were markedly changed over the 21 d of the HS diet and did not return to LS levels. This raises the interesting question of whether extrarenal changes in metabolic function might play an important role in normally protecting the kidneys from the injurious effects of an HS diet and from organs these signals might arise. There are reports that an HS diet alters the gut microbiome^75,76^ and liver metabolism,^77^ which need to be explored in greater depth.

### Effects of the HS Diet on Arachidonic Acid Metabolism Pathway

Important effects of an HS diet on the arachidonic acid (AA) pathway were identified by both metabolomic and mRNAseq analysis, which found the upregulation of many elements of this pathway significantly altered in the Cx at HS14 with a tendency to return toward LS levels at HS21 (Figure S10). It is well recognized that arachidonic acid is a major component of cell membrane phospholipids in the kidney, which is metabolized by cyclooxygenase (COX), cytochrome P450 monooxygenase (CYP450), lipoxygenase (LOX), and leukotrienes (LTs) enzymes. COX production of prostaglandins (PG) and LTs leads to inflammatory injury in the kidney. CYP450 production of hydroxyeicosatetraenoic acids (19-HETE and 20-HETE) play important roles in tubular ion transport and in modulating tubuloglomerular feedback to regulate the load on the glomerulus.^78^ It is interesting that despite increased expression of AA in the Cx, reduction of *Cyp4a1* and *Cyp4a2* genes was observed. This may reduce expression of 20-HETE, which is known to reduce renal vasoconstriction and renal vascular responses to angiotensin II, endothelin, norepinephrine, nitric oxide, and carbonmonoxide^79^ and play a key role in kidney damage during the inflammatory process. The effects of HS on the AA pathway have been found to contribute importantly to tubular transport, BP salt-sensitivity, and kidney injury in the Dahl SS rat model of hypertension.^80–83^ We also observed a significant increase of TXB2 in the renal Cx at HS14, which appeared to be attenuated by HS21. TXB2 is an inactive metabolite of thromboxane A2 (TXA2), which is a potent vasoconstrictor and can lead to loss of renal structural integrity and inflammatory damage to the kidney.^84,85^

### HS Downregulates the TCA Cycle and Upregulates Glycolysis

There was a marked difference between the effects of the HS diet upon the metabolomic profiles of the Cx and OM. The Cx clearly showed major changes in the metabolic profiles while few changes were seen in the OM in response to the HS diet (Figure 2). One of the most interesting changes found in the Cx was related to the TCA cycle which at HS21 exhibited reductions in citrate, pyruvate, α-ketoglutarate, succinate, and in the mRNA expression of nearly all of the enzymes controlling the activity of the TCA cycle. Conversely, glycolysis appears to be upregulated indicated by related enzymes including hexokinases, pyruvate kinases, and lactate dehydrogenases.

PTs are thought to have limited capacity for glycolysis with energy needs being met by oxidative mitochondrial metabolism making them susceptible to damage with acute reductions of kidney perfusion.^86^ Although not absent, the expression of *HK* in PTs is considered to be small.^16,87,88^ In the present study, a novel finding was the increased expression of glycolysis-related genes in Cx in response to an HS diet. As determined from the qPCR analysis of the isolated proximal tubular segments of rats fed the HS diet, the increased expression of *Hk1* and *Pkm* are consistent with increased activity of the glycolytic pathway (Figures 4 and 5B). Interestingly, similar downregulation of the TCA cycle and upregulation of glycolysis have also been documented in Dahl SS rats following an HS diet.^89,90^ These findings suggest that upregulation of glycolysis in response to an HS diet may be a normal physiological response, which may be exaggerated in SS rats, potentially due to the absence of protective counterregulatory pathways that protect the kidneys against the detrimental effects of HS diets. Despite such metabolic changes, it is presumed that fatty acids continue to serve as the primary energy source in the PT. Further research will be required to comprehensively decipher the intricate dynamics of energy production and alterations in substrate preference in the PT under HS conditions.

It is relevant that the HS diet did not produce major changes in the metabolomic profiles of the OM of SD rats. This strain to maintain normal levels of renal medullary blood perfusion. SS rats have been found to exhibit a rapid 30% reduction of medullary blood perfusion during the first week of an HS diet^30^ and we have found in a proteomic study of isolated mitochondria of these rats a downregulation, which is not observed in salt-insensitive consomic SS.13^BN^ rats.^91^ SS rats also exhibit a significant increase in total RVR when fed an HS diet while salt-insensitive rats (consomic SS.1^BN^ rats) in contrast to a rather reduction in RVR in salt-insensitive SS.1^BN^ and SD rats in the present study.^92^ Protection from renal ischemia, especially in the renal OM of SD rats may preserve metabolic functions of the mTAL as suggested by an absence of a down regulation of the metabolism pathways when fed an HS diet (Figure 2). The downregulation of the TCA cycle proteins has been observed in mitochondria of isolated mTAL of SS rats.^91^ Several studies from our laboratory have shown that reduction of medullary blood flow in the SD rat with chronic medullary infusion of H_2_O_2_ or an SOD inhibitor (DETC) result in a salt-sensitive form of hypertension.^93,94^ So too, reduction of renal medullary oxidative stress in SS rats by intrarenal infusion of L-arginine reduces salt induced hypertension in SS rats.^95^ The lack of significant changes in metabolism-related genes in OM may reflect salt insensitivity in SD.

### Paradoxical Relationship Between Kidney O_2_ Consumption and TCA Cycle Activity

One of the most interesting observations of the present study was the seemingly paradoxical phenomenon of an increase in kidney O_2_ consumption and energy usage in face of a reduction in the TCA cycle activity. What is the source of this additional energy production and O_2_ usage? The data indicate that with the downregulation of the TCA cycle with the HS diet, glycolysis became a dominant source of energy production despite increased RBF increased O_2_ extraction. The increase of renal venous lactate (Figure 6) is consistent with increased activity of the glycolytic pathway. Aerobic glycolysis which was originally described in cancer cells by Warburg in 1921^96^ is also indicated to occur in the kidney^14,97^ and has more recently been suggested to be involved in the metabolic events observed in diabetic kidney disease and ageing.^98,99^ Although the biochemistry of the Warburg effect is not fully understood, this phenomenon is consistent with our current observations.

NADH produced by the activation of glycolysis is not only used for lactate generation, but is also oxidized by the NADH shuttle (eg, the malate–aspartate shuttle). The increased release of pyruvate into the renal vein after HS suggests that not all of the excess NADH in the cytoplasm produced by activation of the glycolytic system is used for lactate production. Enzymes of malate–aspartate shuttle have been identified in the kidney^100^ and gene expression of key enzymes in this pathway was observed in our study. The malate–aspartate shuttle has largely studied in cancer cells, and some suggest that the glucose fermentation (ie, Warburg effect) is a secondary consequence of saturation of the shuttle.^101^ The malate–aspartate shuttle can be stimulated by an increase in glutamine uptake,^102,103^ which we observed (Figure 6). The key enzymes, oxaloacetate transaminase (GOT) and malate dehydrogenase (MDH) activate the shuttle by forming a complex with acetylation^104^ and we observed an increase in *Got* mRNA expression. Given the recognized limitations of predicting the activity of the shuttle from gene expression levels, the data are consistent with the idea that the malate–aspartate shuttle is regenerating NADH inside of the mitochondrial matrix and sustaining oxidative phosphorylation.

### The HS Diet Alters Amino Acid Metabolism

The kidneys play a major role in the homeostasis of the body amino acid pools through the synthesis, degradation, filtration, reabsorption, and urinary excretion of these compounds. Studies carried out in fasted swine found glutamine and proline from the arterial blood are largely disposed of by the kidneys and other amino acids such as serine, tyrosine, and arginine generated and released from the kidneys for export to other tissues.^23^ The current study carried out in unanesthetized nonfasted rats found glutamine was not taken up at LS state but a strong tendency for an increased uptake of glutamine (*P* = .08) was observed during HS feeding. The kidneys also play an important role in protein metabolism, which is filtered by the glomerulus^105^ is taken up into the lysosomes of the tubules and degraded to amino acids.^106,107^ Even the OM of the kidney appears to participate in amino acid metabolic function in the SD rat. Specifically, a clear reduction was observed in the metabolites related to the degradation of lysine in the OM at HS14 (Figure S10B), which then tended to return toward LS levels at HS21. This included reductions of α-ketoglutarate, glutamate, and allysine in the Lysine degradation pathway. Lysine has recently attracted attention for its ability to suppress salt-sensitive hypertension.^108^ Although lysine is one of the essential amino acids, we found it was released from the SD rat kidney after HS (Figure 6), consistent with observations by Jang et al.^23^ in pig study. This is likely explained by the degradation of protein either by glomerular epithelial or tubular lysosomes during renal passage.^109–111^ Moreover, we observed almost all the amino acids were released from kidney into renal vein in SD rats fed LS and further release was observed in most of detected amino acids when fed HS. In general, the data indicate that the kidney produces amino acids from protein degradation faster than their utilization by the kidney and that the HS diet enhanced proteolysis. There were, however, several notable exceptions to this such as glutamine, which was released from the kidney at LS and tended to be taken up after HS. This uptake of glutamine may be involved in the activation of the malate–aspartate shuttle pathway as discussed earlier.

It was also found that the megalin (*Lrp2*) and clathrin (*Cltc*) mRNA expression levels were reduced with the HS diet (Figure S8) whereas several proteases and plasmid partitioning (PAR genes) were upregulated in Cx. Megalin and clathrin are key players in apical endocytosis in PT and reduction of these proteins is related to a reduction in albumin endocytosis.^111,112^ Megalin is downregulated with HS diets even in salt insensitive Wistar or SD rats and in the absence of increased urinary albumin excretion.^113,114^ However, as discussed earlier, the fact that the expression of transporter genes in the renal cortical PTs appear to be decreased while the expression of genes distal to the TAL is increased suggests that proteolysis may be shifted to the distal tubules. For example, *Ctsa* and *Ctsb*, which are predominantly expressed in the PTs, are downregulated, while *Ctsc* and *Ctsd*, which are highly expressed in the DCT and other areas,^61^ are upregulated after salt loading. *Lrp2* and *Cltc* expressed in PT but not in DCT. It is known that DCT performs endocytosis of proteins, but it is not known which proteins play a key role, and further segment-specific studies are needed.

#### Limitations and Ultimate Goals

The present study provides a unique data set obtained in unanesthetized normal SD rats in which the metabolomic and genomic transcriptional responses to an HS diet with each rat serving as their own control, before and following the switch of diet. While avoiding the stress of surgery and anesthesia, by its nature this approach was limited to obtaining only global “solute mass balance” data reflecting metabolism of the whole kidney and was unable to distinguish between the cortex and medulla of the kidney. For this reason, parallel groups of rats were studied to obtain cortical and OM tissue for analysis at comparable days of HS loading. As such, it is recognized that the limitation of these data are biased to some extent by the unavoidable effects of anesthesia-surgery and procedures required to obtain these tissues. The isolation of tubules for such analyses is yet another step removed from the “normal” physiological state but the results of the targeted mRNA analysis appear to validate conclusions drawn from the mRNAseq analysis of cortical tissue. Regarding the purity concern for microdissection, it is shown in Figure 5A that there is a 1000 times difference in *Nkcc2* between isolated PT and non-PT. This indicates that even a difference of 1/1000 would be detectable if contamination were to occur in one group. Although 100% purity of PT is unlikely, contamination from non-PT segments or glomeruli found in the present study could not account for the doubling of *Hk1* expression in the PT segments of SD rats fed an HS diet. It should also be noted that we have not obtained absolute copy number per cell from our analysis of the isolated PTs and only relative changes are represented.

Other limitations of the present study must also be considered. RBF was normalized by total body weight although conventionally reported in terms of volume flow per kidney weight. This was necessary since with RBF is continuously measured over nearly 4-wk so normalization to kidney weight could not be done for the intermittent time points of the study since kidneys were weighted only at the end of the 21-d period of the HS diet. Although in other studies of SD rats that we have carried out, we have found no difference in kidney size comparing SD rats fed LS or HS for 21 d, RBF in the present study was nonetheless normalized by body weight. Another limitation of the present study is that although all samples were collected at the same time of day to avoid diurnal effects (both from the unanesthetized and anesthetized rats) the rats were not fasted so ad lib eating/drinking could increase the variance of the data.

Finally, the limitations imposed by the evolving field of untargeted global metabolomics are a consideration for all such studies in this field. Despite the solid progress that has been made in metabolite identification and the sensitivity of the mass spectrometry techniques, the capability of detection and identification of compounds is far from comprehensive. Verification is important especially for compounds with low FISH scores, and it is evident that continuing efforts must be made in the development of the databases and informatics to link substrates, enzymes, and metabolites to biochemical and physiological pathways. The present study provides only directional changes in metabolite concentrations and it is evident that greater numbers of pure standards will be needed to enable large scale quantitative analysis. Going forward, it will also be important to trace the fate of administered isotope-labeled metabolites to validate the hypotheses that are generated based on metabolic flux studies since we are currently only able to calculate net consumption or net production in the kidney (eg, solute mass balances). Finally, sex differences of kidney metabolism were not assessed as the chronic instrumentation of the smaller female rats when age matched proved to be overly daunting.

## Conclusion

The kidneys of even normal SD rats with low blood pressure salt-sensitivity exhibited significant changes in the metabolomic profiles in order to sustain the increased transport workloads and energy needs of the kidneys. The temporal patterns identified unique metabolic changes in the first 14 d of HS followed by what appear to be compensatory response required to sustain the energy requirements of the kidney. It seems that, at least at the mRNA level, the glycolysis was enhanced, and the production of pyruvate and lactate increased despite the increased oxygen consumption, while the TCA cycle was down regulated by the HS diet in SD rat’s kidney cortex. NADH produced during the process of increased glycolysis is used for lactate production in the cytoplasm and is involved in oxidative phosphorylation in mitochondria via malate–aspartate shuttle, which could potentially contribute to oxygen consumption. Besides the activation of glycolysis, the oxidative PPP, uncoupling protein 2 and nitric oxide synthetase 3 were upregulated each of which scavenge ROS and protect against kidney damage in SD rat’s kidney. The progressive increase of energy required for the HS diet in face of a reduction in TCA cycle activity may be sustained by a “Warburg-like” effect whereby glucose and other 6-carbon sugars are converted by glycolysis into cellular energy and the metabolite lactate, which we found elevated in the renal venous blood (eg, lactic acid fermentation). Although not previously identified in kidney cells, it is recognized that lactic acid fermentation can occur in muscle cells undergoing intense activity enabling ATP and NAD^+^ production to continue glycolysis.^115^ Finally, although kidney proteolysis appears to be enhanced by the HS diet, the metabolic consequences of this is unclear.

## Supplementary Material

zqad031_Supplemental_Files

## Data Availability

The data underlying this article will be shared on reasonable request to the corresponding author. mRNAseq data are available from GEO (GSE224984). Metabolomics data are available from Metabolomics Workbench (PR001719) DOI: http://dx.doi.org/10.21228/M8HF01.
