# Supplementary material for: Metabolic Responses of Normal Rat Kidneys to a High Salt Intake
Source: Function (Oxf). 2023 Jun 22;4(5):zqad031. doi: 10.1093/function/zqad031 (PMC10413938; doi:10.1093/function/zqad031)
Supplement: zqad031_Supplemental_Files [file zqad031_supplemental_files.zip › Supplementary material files/Supplemental Information.pdf]

SUPPLEMENTAL INFORMATION

Title: Metabolic responses of normal rat kidneys to a high salt intake

Authors and affiliations:

Satoshi Shimada[1], Brian R. Hoffmann[2], Chun Yang[1], Theresa Kurth[1], Andrew S. Greene[2], Mingyu Liang [1], Ranjan K. Dash[1,3], Allen W. Cowley Jr[1] *.

1. Department of Physiology, Medical College of Wisconsin, Milwaukee, Wisconsin, USA.
2. Mass Spectrometry and Protein Chemistry, Protein Sciences, The Jackson Laboratory, Bar Harbor, Maine, USA
3. Department of Biomedical Engineering, Medical College of Wisconsin and Marquette University, Milwaukee, Wisconsin, USA.

*Corresponding author

Allen W. Cowley, Jr., Ph.D.

Department of Physiology, Medical College of Wisconsin

8701 Watertown Plank Rd, Milwaukee, WI 53226, USA

Tel: +1-414-955-8277

Fax: +1-414-955-6546

cowley@mcw.edu

Methods

mRNAseq analysis at Novogene

Library preparation for Transcriptome sequencing

Messenger RNA was purified from total RNA using poly-T oligo-attached magnetic beads. Following fragmentation, first strand cDNA was synthesized using random hexamer primers followed by double-stranded non-directional cDNA synthesis. After end repair, A-tailing, adapter ligation, size selection, USER enzyme digestion, amplification, and purification the library was checked with Qubit and real-time PCR for quantification and bioanalyzer for size distribution detection. Quantified libraries were pooled and sequenced on Illumina platforms, according to effective library concentration and data amount.

Quality Control

Raw data (raw reads) of fastq format were first processed through in-house perl scripts in which clean reads were obtained by removing from the raw data those containing adapters, reads containing ploy-N and low quality reads. From the Q20, Q30 and GC content the clean data were calculated. All the downstream analyses were based on these clean high quality data with the results of these QC steps summarized in **Table S2**.

Reads mapping to the reference genome

Reference genome and gene model annotation files (ensembl_rattus_norvegicus_rnor_6_0_gca_000001895_4) were downloaded from the genome website directly. The index of the reference genome was built using Hisat2 v2.0.5 and paired-end clean reads were aligned to the reference genome using Hisat2 v2.0.5. The result of the Mapping is summarized in **Table S3**, and the results of the Mapping region is summarized in **Table S4**.

Novel transcripts prediction

The mapped reads of each sample were assembled by StringTie (v1.3.3b) (Mihaela Pertea.et al. 2015) which uses a network flow algorithm and an optional de novo assembly step to assemble and quantitate full length transcripts representing multiple splice variants for each gene locus.

Quantification of gene expression level

FPKM, the expected number of Fragments Per Kilobase of transcript sequence per millions base pairs sequenced, was used for estimating gene expression levels. FeatureCounts v1.5.0-p3 was used to count the read numbers mapped to each gene from which the FPKM of each gene was calculated based on the length of the gene and reads count mapped to this gene. Results of gene expressions are provided as a supplementary file of GSE224984.

Differential expression analysis

Two biological replicates per condition were determined using the DESeq2 R package (1.20.0) which provided statistical routines for determining differential expression of the digital gene expression data using a model based on the negative binomial distribution. Prior to differential gene expression analysis, for each sequenced library, the read counts were adjusted by edgeR program package (3.22.5) through one scaling normalized factor the P values of differential expression were adjusted using the Benjamini & Hochberg method to control for the false discovery rate. The corrected p-value of 0.05 were set as the threshold for significantly differential expression.

KEGG enrichment analysis of differentially expressed genes

To ascertain biological functions or associated pathways significantly associated with differentially expressed genes (adj $p<0.05$), enrichment analysis was performed using clusterProfiler software. The enrichment test for KEGG pathways was calculated based on hypergeometric distribution.

Analysis software list

Software and parameters for the RNAseq analysis are summarized in **Table S5**.

<u>Isolation of nephron segments</u>

The separate group of male SD rats (n=12, 12-14 weeks of age) were fed either 0.4% or 21 days of 4.0% salt diet. For nephron segments isolation, rats were anesthetized with thiobutabarbital (50 mg/kg) and the kidneys were flushed with saline and then a collagenase digestion solution [Hanks' balanced salt solution (HBSS) containing 20 mM HEPES (HBSS-H), pH 7.4, with collagenase type 2 (200 U/mL)] at 4 mL/min. After the flushing, kidneys were removed, and the cortex tissue was cut into small pieces and then incubated in a collagenase digestion solution

(collagenase type 2 400U/ml in HBSS-H) at 37°C for 30 min. The digestion suspensions were washed twice by 1% BSA-HBSS-H. Different nephron segments (about 50-100 pieces) were manually picked from the suspensions under a microscope (model SMZ 45, Nikon) including glomerulus, proximal tubule and non-proximal cortical tubules (including cTAL, DCT and CCD which were hard to distinguish visually and had less content in the suspensions). Collections were snap frozen and saved at -80°C.

Quantification of RNA expression levels

Total RNA was extracted from the frozen nephron segments using Trizol reagent (Life Technologies) following manufacture's recommendation. The quantity of isolated RNA was determined by spectrophotometry (Nanodrop). About 1 μg of total RNA were reverse transcribed by random hexamer primers into complementary DNA using Revert Aid First Strand cDNA synthesis kit (Thermo Scientific). Then The target cDNAs were amplified with specific primers (Table S6) in 10 ul system of 1x EvaGreen qPCR Master Mix (MidSci) with 10 ng cDNA using SYBR green as indicator dye on a QuantStudio 6 Flex System (Applied Biosystems). Each mRNA copy number was normalized to the expression of 18S ribosomal RNA.

Figure S1.

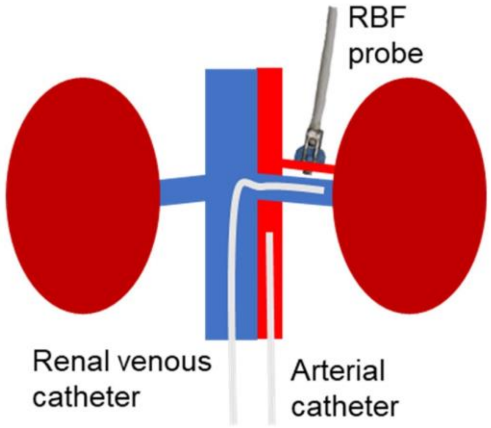

Figure S1. The schema of the chronically instrumented rat. Illustrated is the ultrasonic flow probe on the left renal artery to measure renal blood flow (RBF) and the chronically implanted aortic and renal venous catheters for intermittent sampling of blood.

Figure S2.

Aspartic Acid

(C18+, m/z=133.03791; FISH= 16.67)

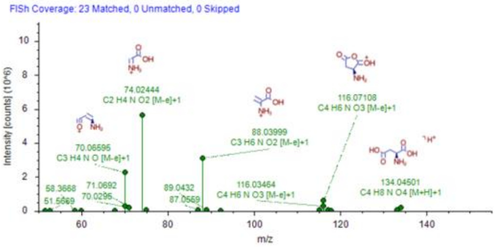

Glutamic Acid

(C18+; m/z=147.05336; FISH = 34.75)

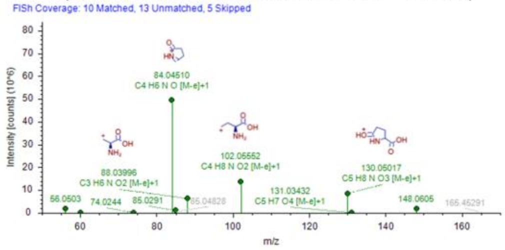

Tryptophan

(C18+, m/z=204.09001; FISH = 26.37)

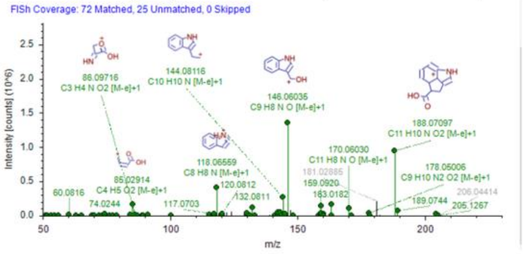

Phenylalanine

(C18+, m/z=165.07918; FISH=54.76)

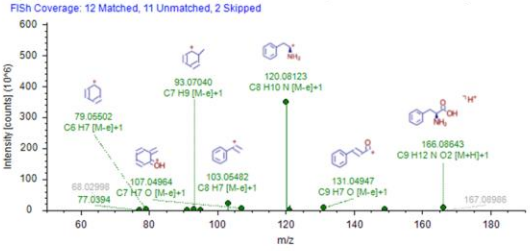

Glucose

(C18-; m/z=180.06277; FISH= 57.50)

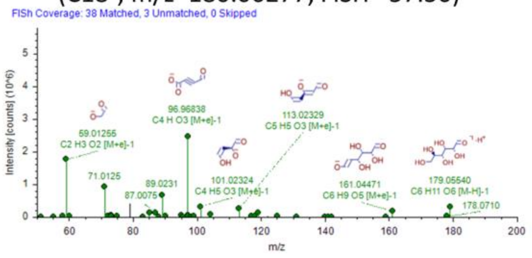

Pyruvate

(C18-; m/z=88.01470; FISH= 20.00)

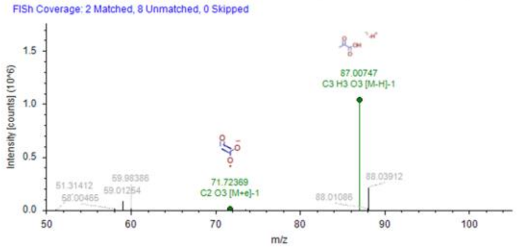

Lactate

(C18-; m/z=90.03032; FISH=45.45)

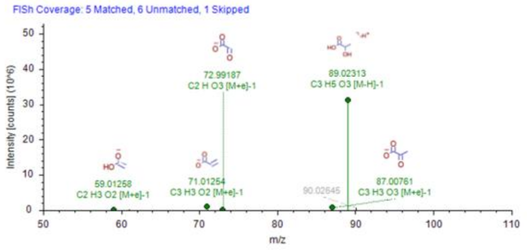

Citrate

(C18-; m/z=192.02620; FISH=54.55)

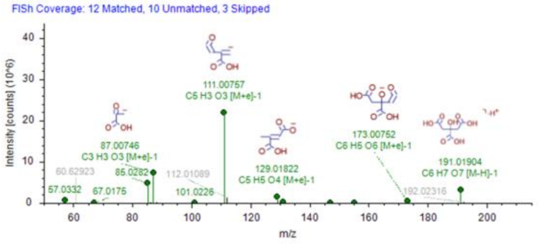

Lysine

(C18+; m/z=146.10583; FISH=62.79)

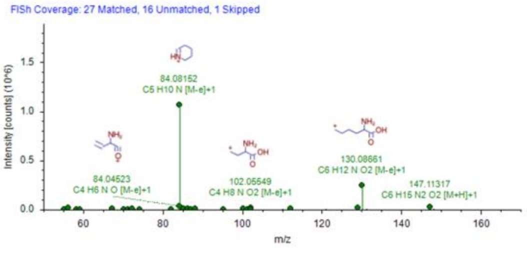

Alpha-Ketoglutarate

(C18+; m/z=146.02152; FISH=87.76)

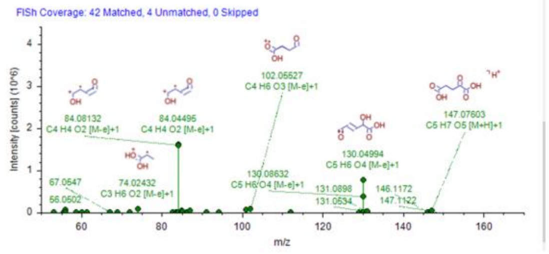

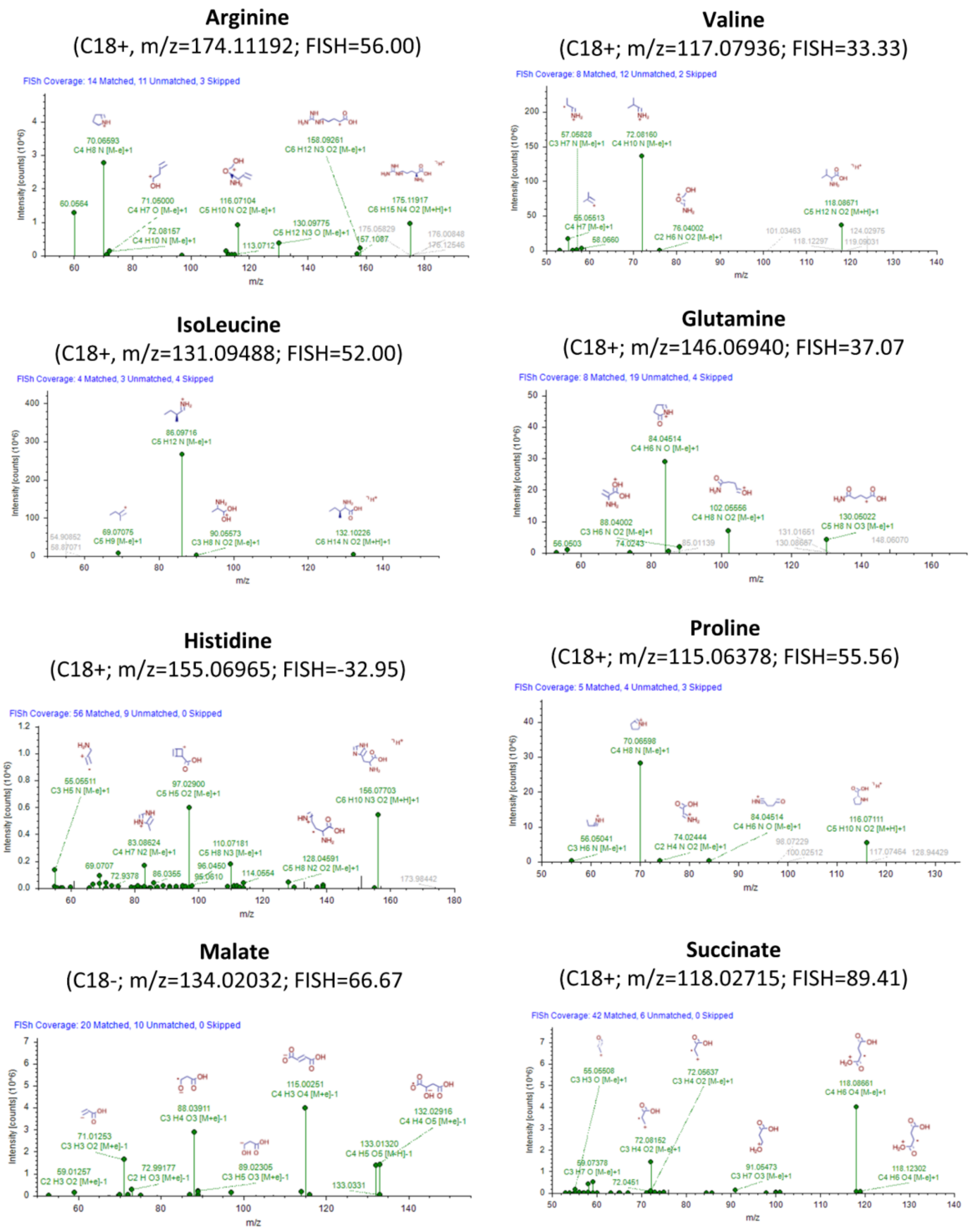

Figure S2. Supplied are examples of the MS2 fragmentation match that is completed in the analysis of our targets versus our standard database. All annotated metabolites in the data files go through initial FISH scoring (MS2 scoring) with a filter of equal to or greater than 10. Additional manual checking of the MS2 spectra matching is then completed for any targets specifically focused on as a main point of the study in the associated figures. Here are example MS2 spectra matches showing high-confidence matching for analytes.

Figure S3.

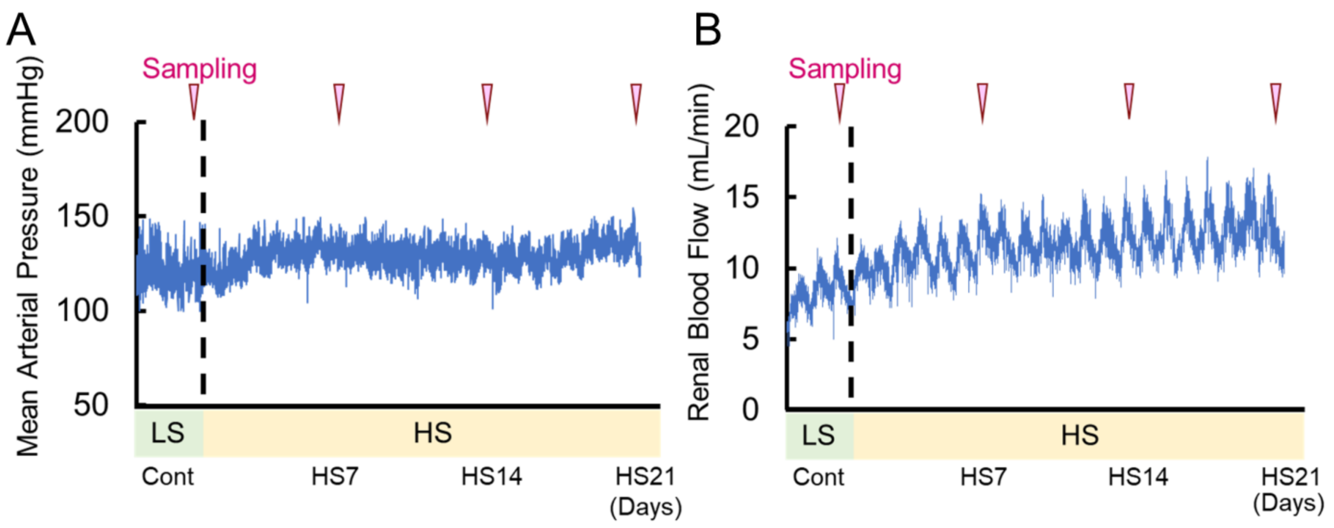

Figure S3. Representative (n=1) 1 min average of (A) mean arterial pressure and (B) renal blood flow, and periods of sampling for bloods and urine.

Figure S4.

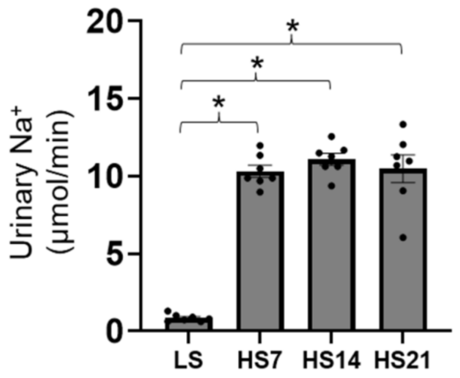

Figure S4. Urinary Na^+ excretion rates (n=7 rats) at each of the days of sampling (LS, HS7, HS14, and HS21. Mean ± SEM and individual data. *p<0.05 vs LS, One-way RM ANOVA, Holm-Sidak.

Figure S5.

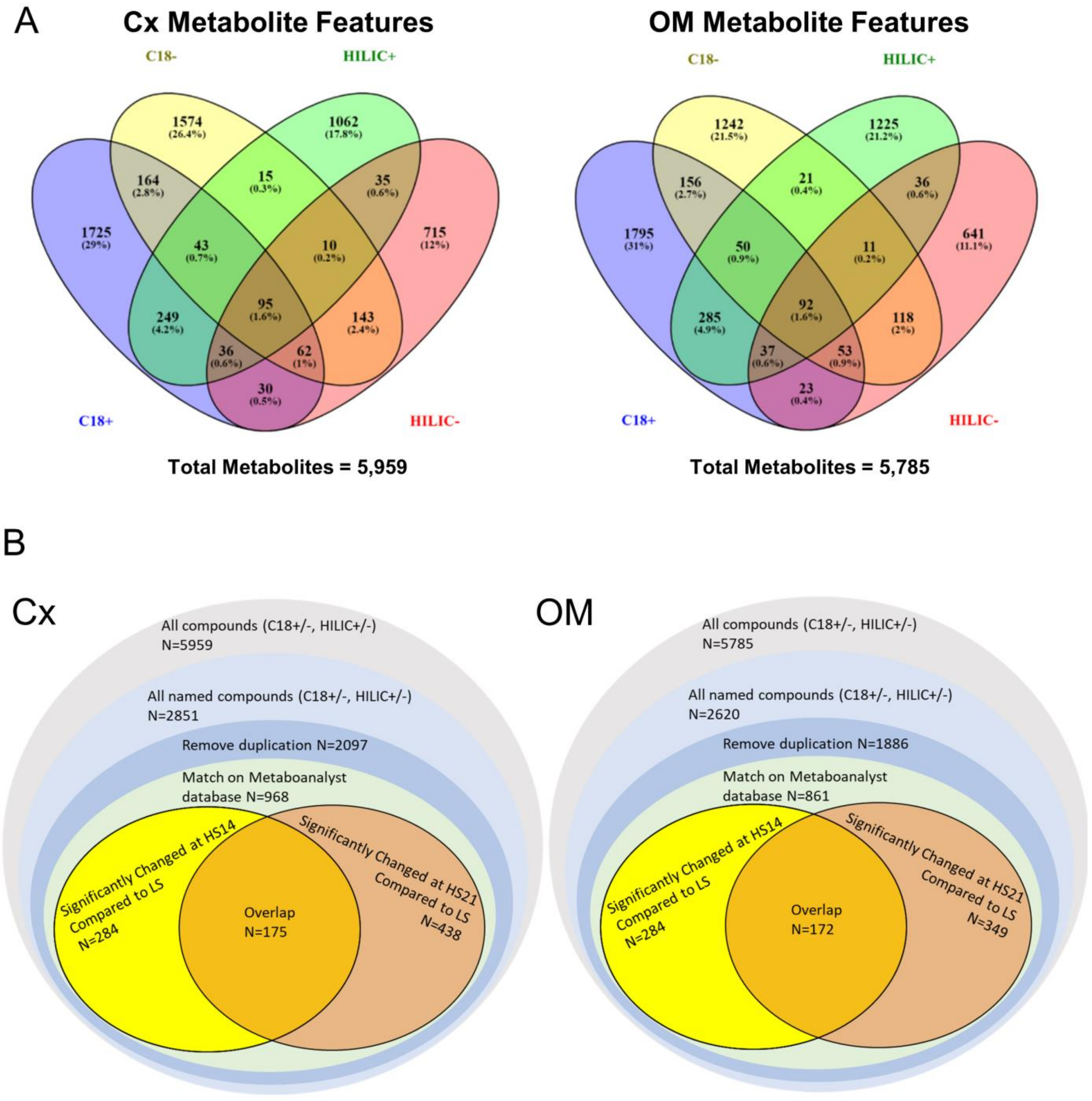

Figure S5. Venn diagrams of cortical (Cx) and outer medullary (OM) tissue metabolite features showing the number of metabolites detected in each of 4 modes (C18+/-, HILIC+/-). (A) Illustrates the number of metabolites detected, named and listed in the Metaboanalyst 5.0 database (October 2022). (B). Illustrates the number of those that significantly ($p<0.05$) differed from LS at HS by t-test.

Figure S6.

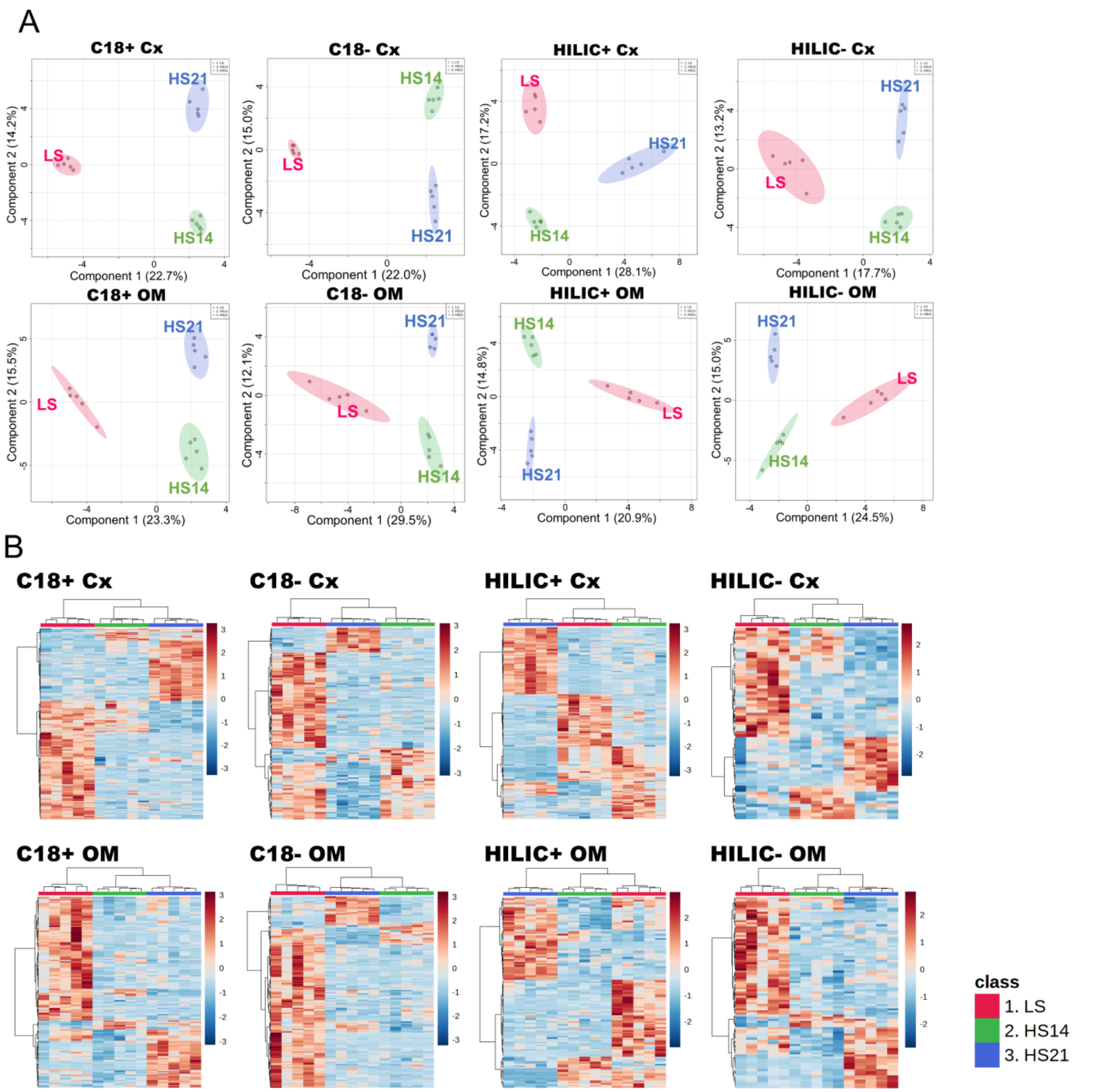

Figure S6. Sparse Partial Least-Squares Discriminant Analysis (sPLS-DA) of cortical (Cx) and outer medullary (OM) tissues in each of 4 modes. (A) Parameters for sPLS-DA are fixed to 5 variables per component and to 20 for validation yielding 5-fold difference in coefficient of variation. Ellipses represent 95% confidence region of a bivariate normal distribution. (B) For those compounds significantly affected by high-salt (HS) (ANOVA Fisher's LSD $p<0.05$) a hierarchical cluster analysis was performed with a Euclidean distance measure and by the Ward algorithm and represented by heatmaps. Red: Low salt (LS), Green: 14 days of HS, Blue: 21 days of HS.

Figure S7.

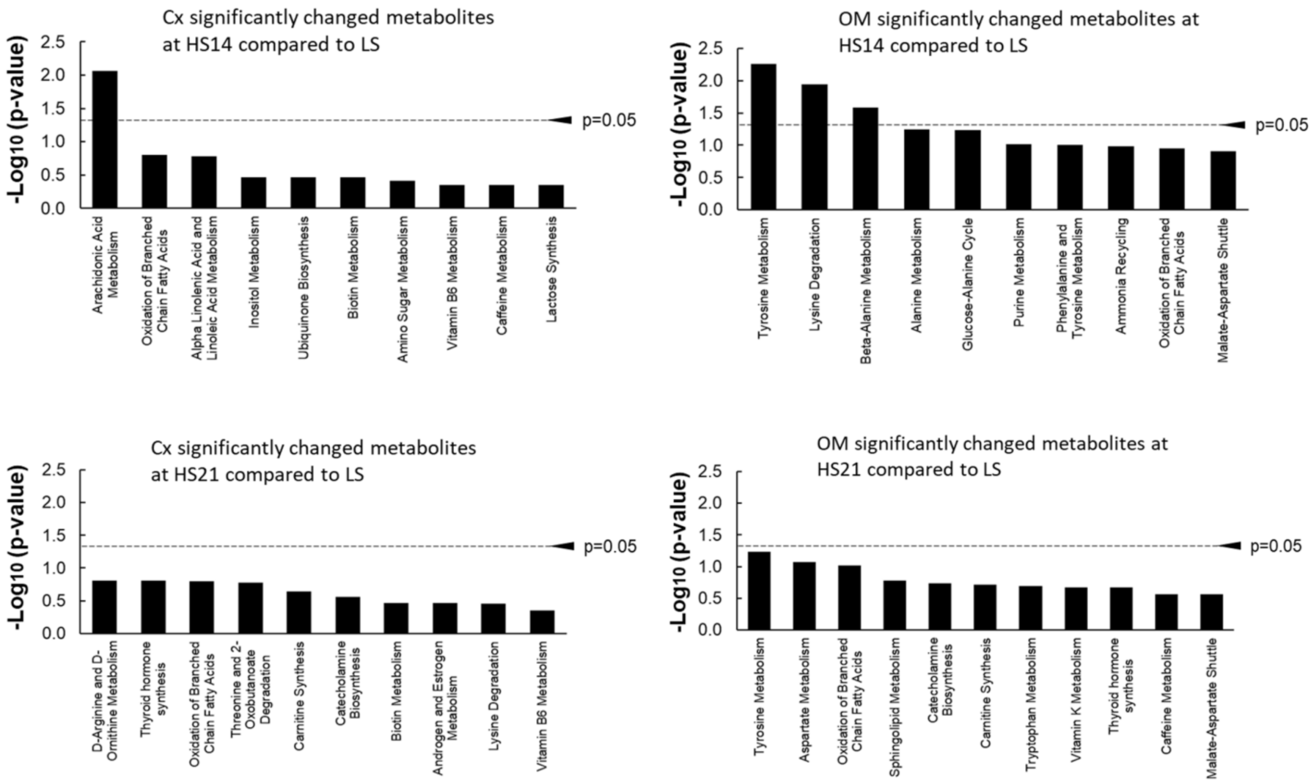

Figure S7. Results of the top 10 pathways identified in the cortex (Cx) and outer medulla (OM) from the metabolomic analysis determined by enrichment analysis on Metaboanalyst 5.0 (SMPDB, October 2022). Shown are pathways that were changed at HS days 14 and 21 compared to LS fed rats. The $-\log_{10}$ p-values are plotted with those above the dotted horizontal line representing $p<0.05$.

Figure S8.

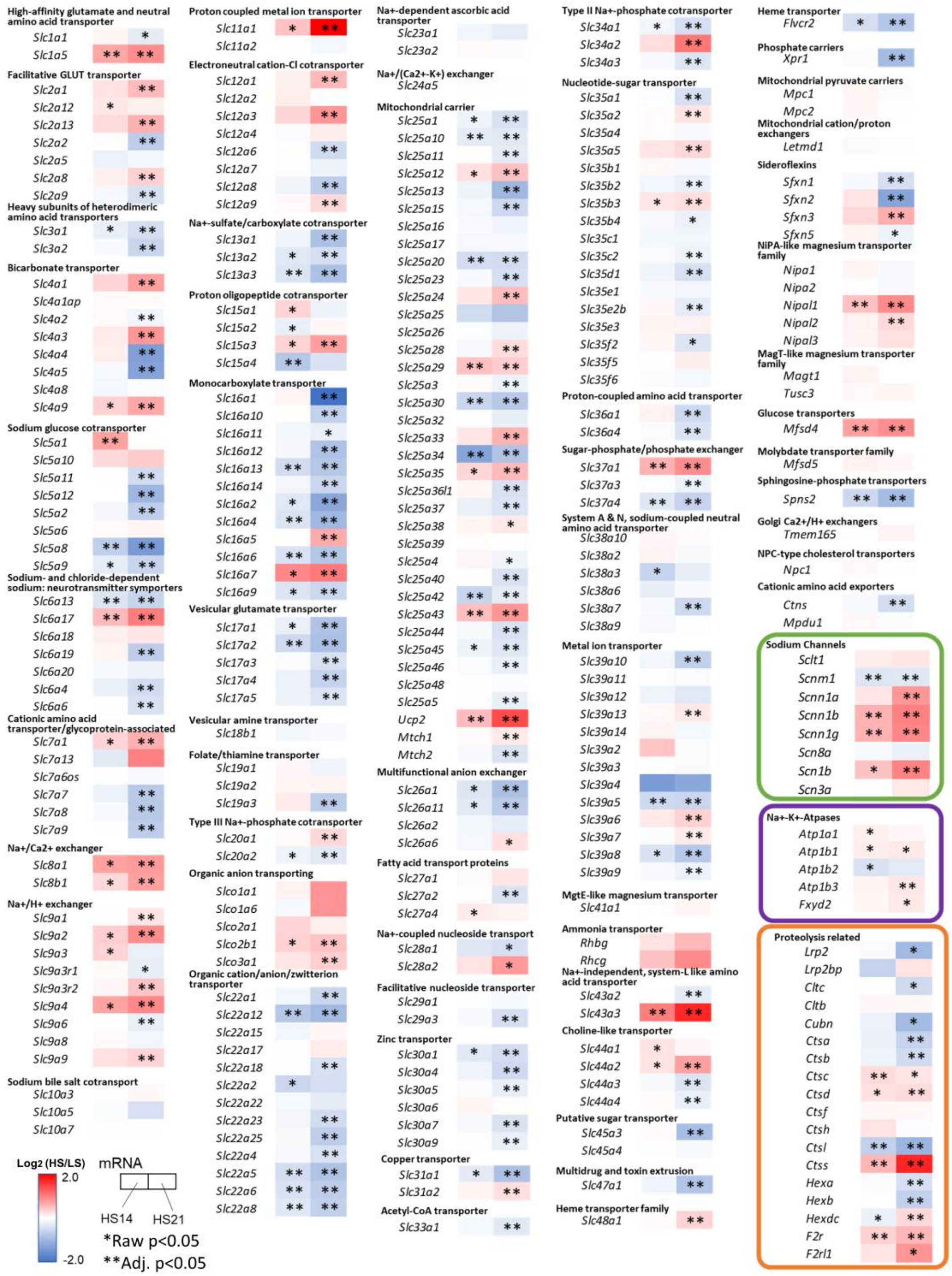

Figure S8. mRNA expression of transporters, channels, Na^{+}-K^{+}-ATP^{+}ases and proteolysis of renal cortex (Cx) comparing the binomial logarithm of the ratio of high salt (HS) days 14 and 21 to low salt (LS). Red denotes increase in expression and blue denotes decrease in expression. Not framed are solute carrier family genes, framed in green are sodium channels, framed in purple are Na^{+}-K^{+}-ATP^{+}ases and framed in orange are proteolysis related genes. *Raw p<0.05 in DESeq2, **Adj. p<0.05 in Benjamini and Hochberg.

Figure S9.

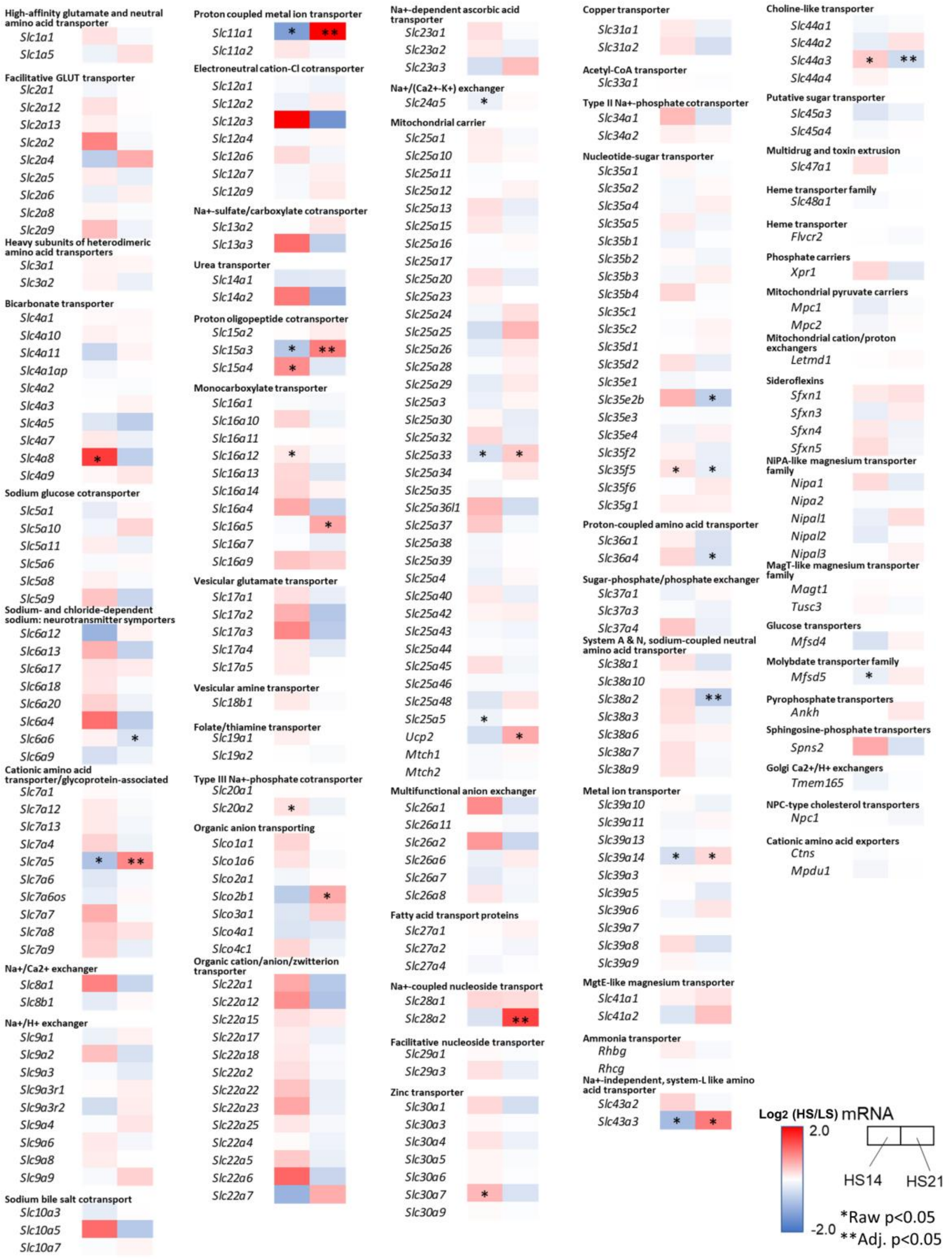

Figure S9. mRNA expression of transporter in outer medulla (OM).

The binomial logarithm of the ratio of high salt (HS) to low salt (LS) are represented in color. The left boxes are ratio of HS14 to LS and the right boxes are ratio of HS21 to LS. Red denotes increase in expression and blue denotes decrease in expression *Raw p<0.05 in DESeq2, **Adj. p<0.05 in Benjamini and Hochberg.

Figure S10.

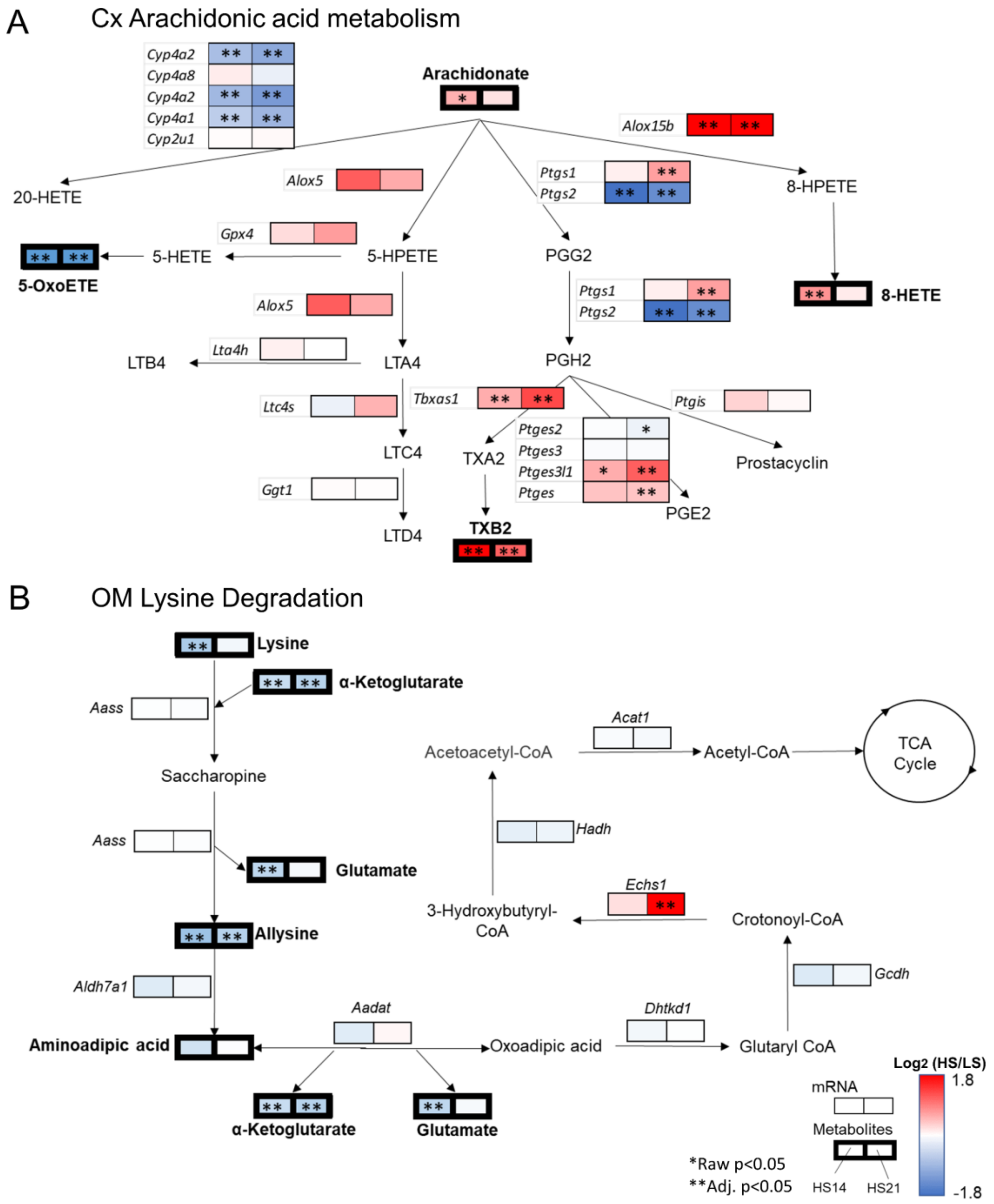

Figure S10. Integrated figure of arachidonic acid metabolism in cortex (Cx) (A) and lysine metabolism in outer medulla (OM) (B).

The binomial logarithm of the ratio of high salt (HS) to low salt (LS) are represented in color. The left boxes are ratio of HS14 to LS and the right boxes are ratio of HS21 to LS. Thin boxes represent mRNA and thick boxes represent metabolites. Red denotes increase in expression and blue denotes decrease in expression. *Raw p<0.05 in t-test for metabolomics and in DESeq2 for mRNAseq, **Adj. p<0.05 in Benjamini and Hochberg. (KEGG map ID 00590 last update: 28 September 2022, SMP0000037 last update: 24 November 2022)

Figure S11.

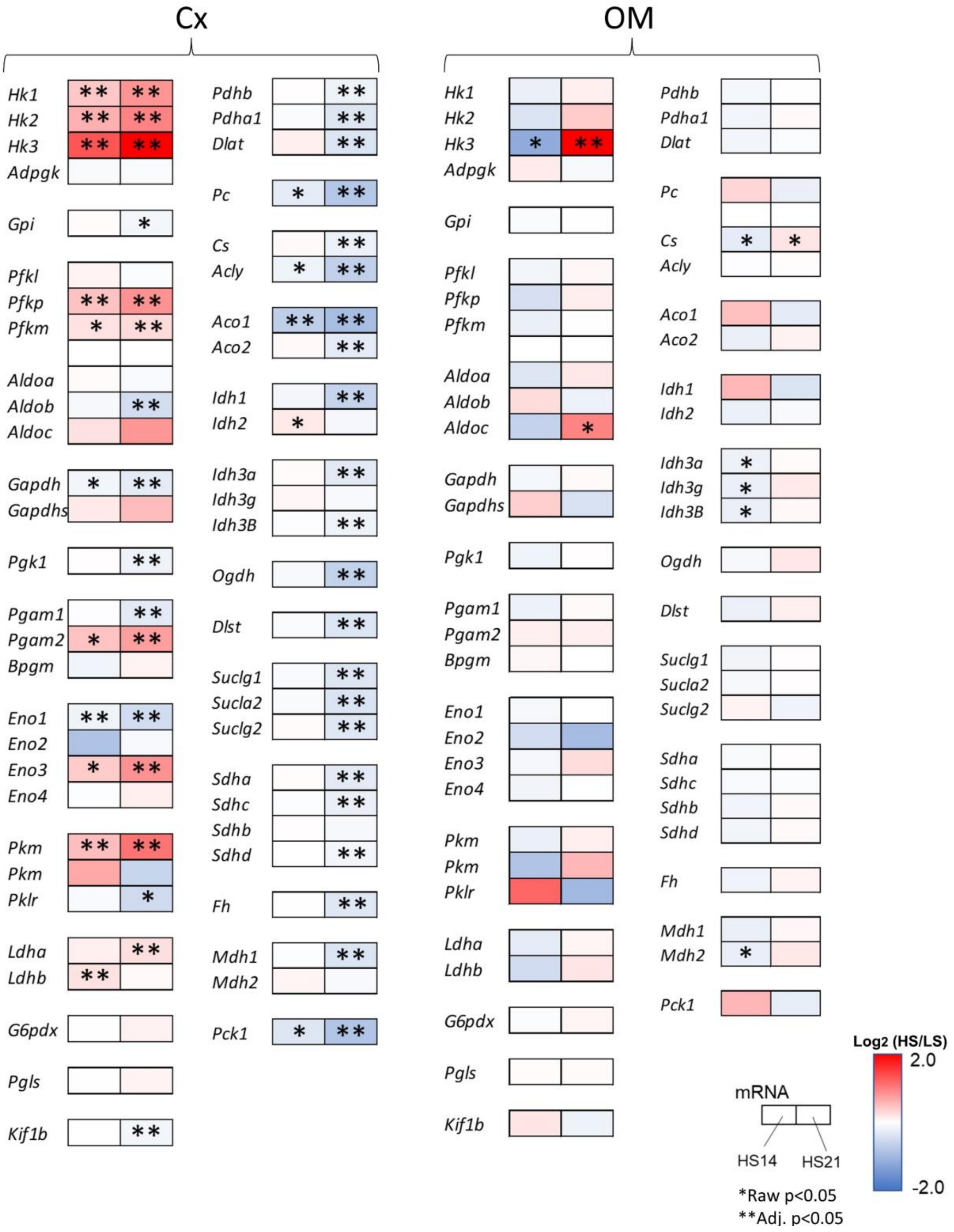

Figure S11. Gene expression of glycolysis and TCA cycle in cortex (Cx) and outer medulla (OM) The binomial logarithm of the ratio of high salt (HS) to low salt (LS) are represented in color. The left boxes are ratio of HS14 to LS and the right boxes are ratio of HS21 to LS. Red denotes increase in expression and blue denotes decrease in expression *Raw $p<0.05$ in DESeq2, **Adj. $p<0.05$ in Benjamini and Hochberg.

Figure S12.

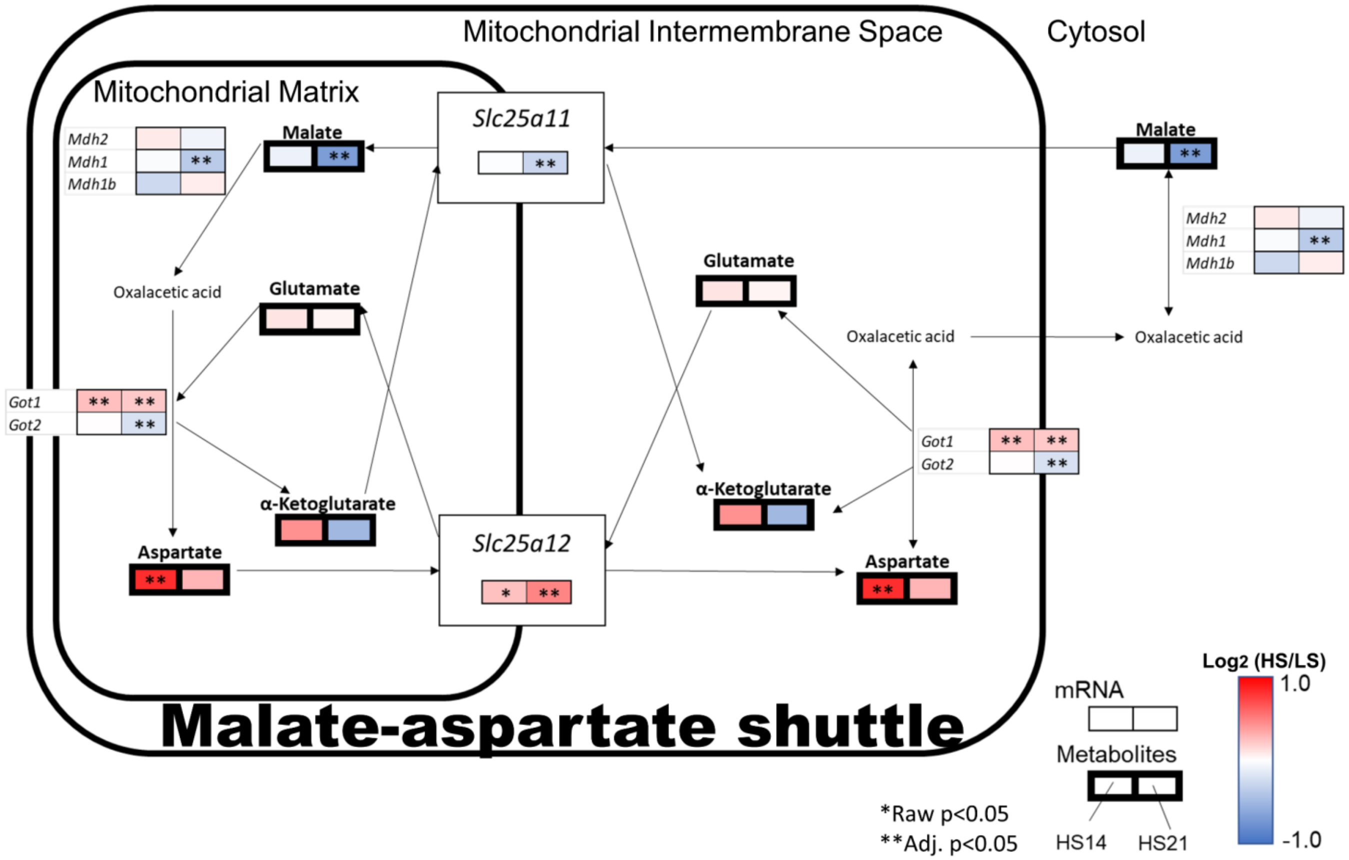

Figure S12. Integrated figure of malate-aspartate shuttle in cortex

The binomial logarithm of the ratio of high salt (HS) to low salt (LS) are represented in color. The left boxes are ratio of HS14 to LS and the right boxes are ratio of HS21 to LS. Thin boxes represent mRNA and thick boxes represent metabolites. Red denotes increase in expression and blue denotes decrease in expression. *Raw $p<0.05$ in t-test for metabolomics and in DESeq2 for mRNAseq, **Adj. $p<0.05$ in Benjamini and Hochberg. (SMP0000129 last update: 18 October 2022)

Figure S13.

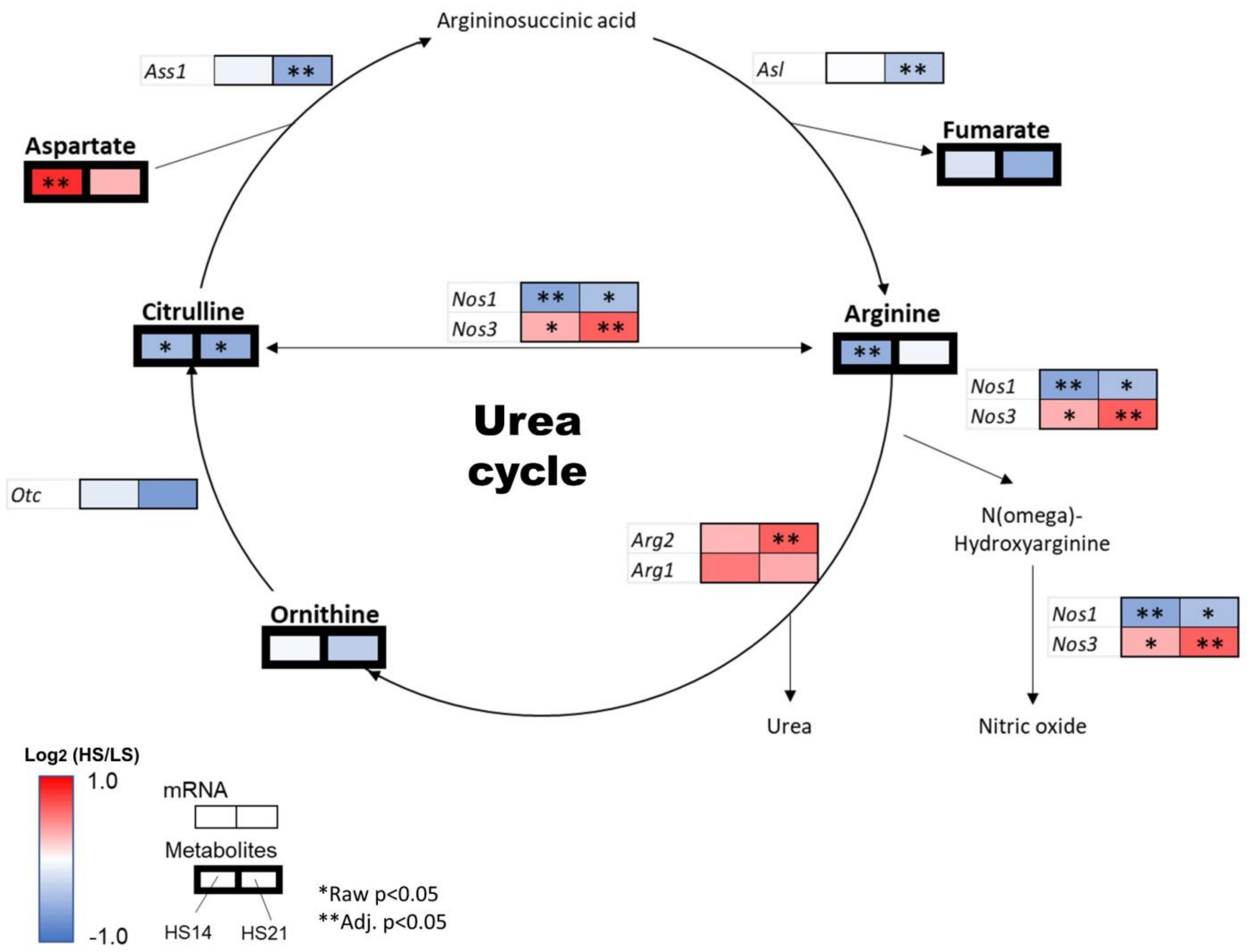

Figure S13. Integrated figure of urea cycle in cortex

The binomial logarithm of the ratio of high salt (HS) to low salt (LS) are represented in color. The left boxes are ratio of HS14 to LS and the right boxes are ratio of HS21 to LS. Thin boxes represent mRNA and thick boxes represent metabolites. Red denotes increase in expression and blue denotes decrease in expression. *Raw p<0.05 in t-test for metabolomics and in DESeq2 for mRNAseq, **Adj. p<0.05 in Benjamini and Hochberg. (KEGG map ID 00220 last update: 29 July 2022)

Figure S14.

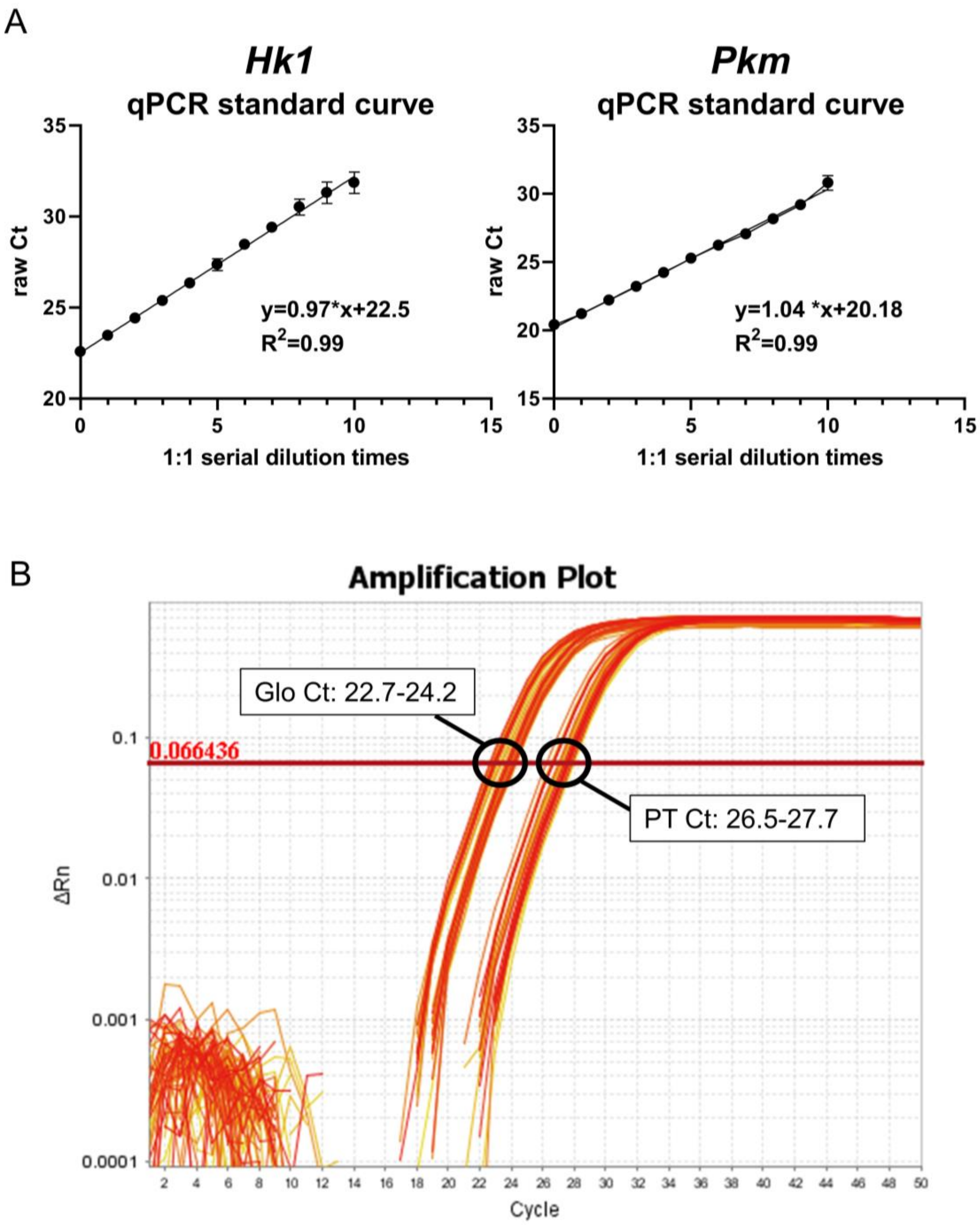

Figure S14. qPCR standard curve and amplification plot

(A) qPCR was performed for cDNA of rat kidney cortex tissue. Starting with 10 ng, Ct values at each dilution ratio were determined. The horizontal axis shows the times of dilution, and the vertical axis shows the Ct value. Tests results for *Hk1* and *Pkm* primers (**Table S6**) are shown. (B) Amplification plot of glomerular (Glo) and proximal tubule (PT) samples for *Hk1*. Range of Ct values are 26.5-27.7 for PT and 22.7-24.2 for Glo.

Figure S15.

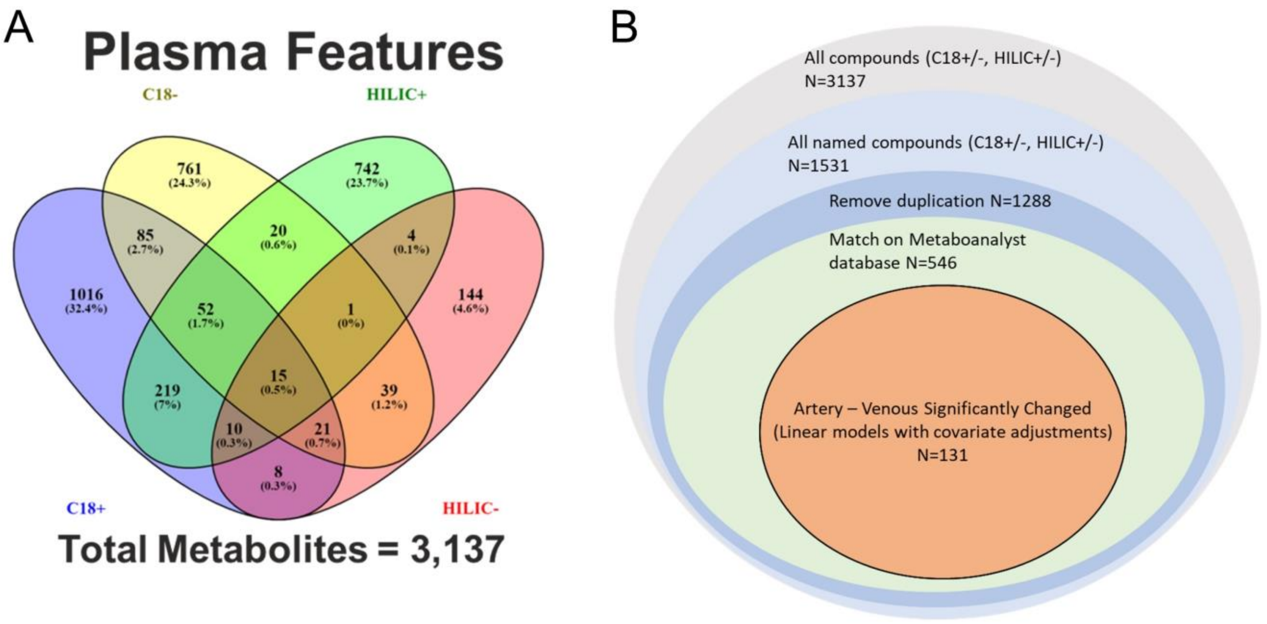

Figure S15. Metabolite features in plasma.

Venn diagram shows the number of metabolites detected in each of 4 modes (C18+/-, HILIC+/-) in plasma (A). The number of metabolites detected, named and listed in the Metaboanalyst 5.0 database (November 2022) (B). The number of those that artery and venous difference are significantly ($p<0.05$) differed from LS at HS by linear models with covariate adjustment is also shown.

Figure S16.

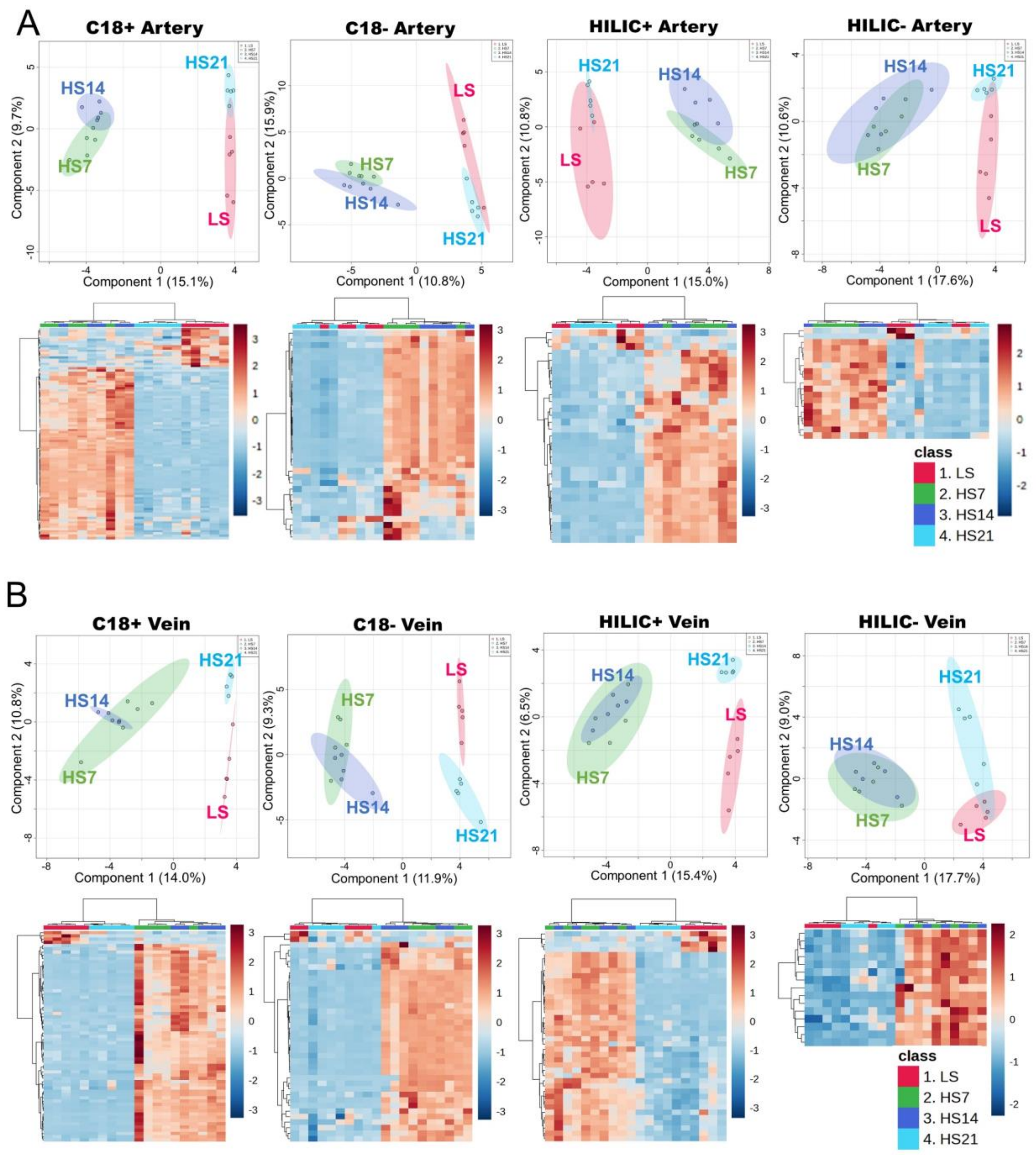

Figure S16. sPLS-DA and heatmap of plasma

Sparse Partial Least-Squares Discriminant Analysis (sPLS-DA) of arterial and venous plasma in each of 4 modes (A). Parameters for sPLS-DA are fixed to number of components: 5, variables per component: 20 and validation method: 5-fold CV. Ellipses represent 95% confidence region of a bivariate normal distribution. Compounds which are significantly affected by high-salt diet (HS) (ANOVA Fisher's LSD p<0.05) are performed hierarchical cluster analysis with a Euclidean distance measure and by the Ward algorithm and represented by heatmaps (B). Red: Low salt (LS), Green: 7 days of HS, Blue: 14 days of HS, Light blue: 21 days of HS.

Figure S17.

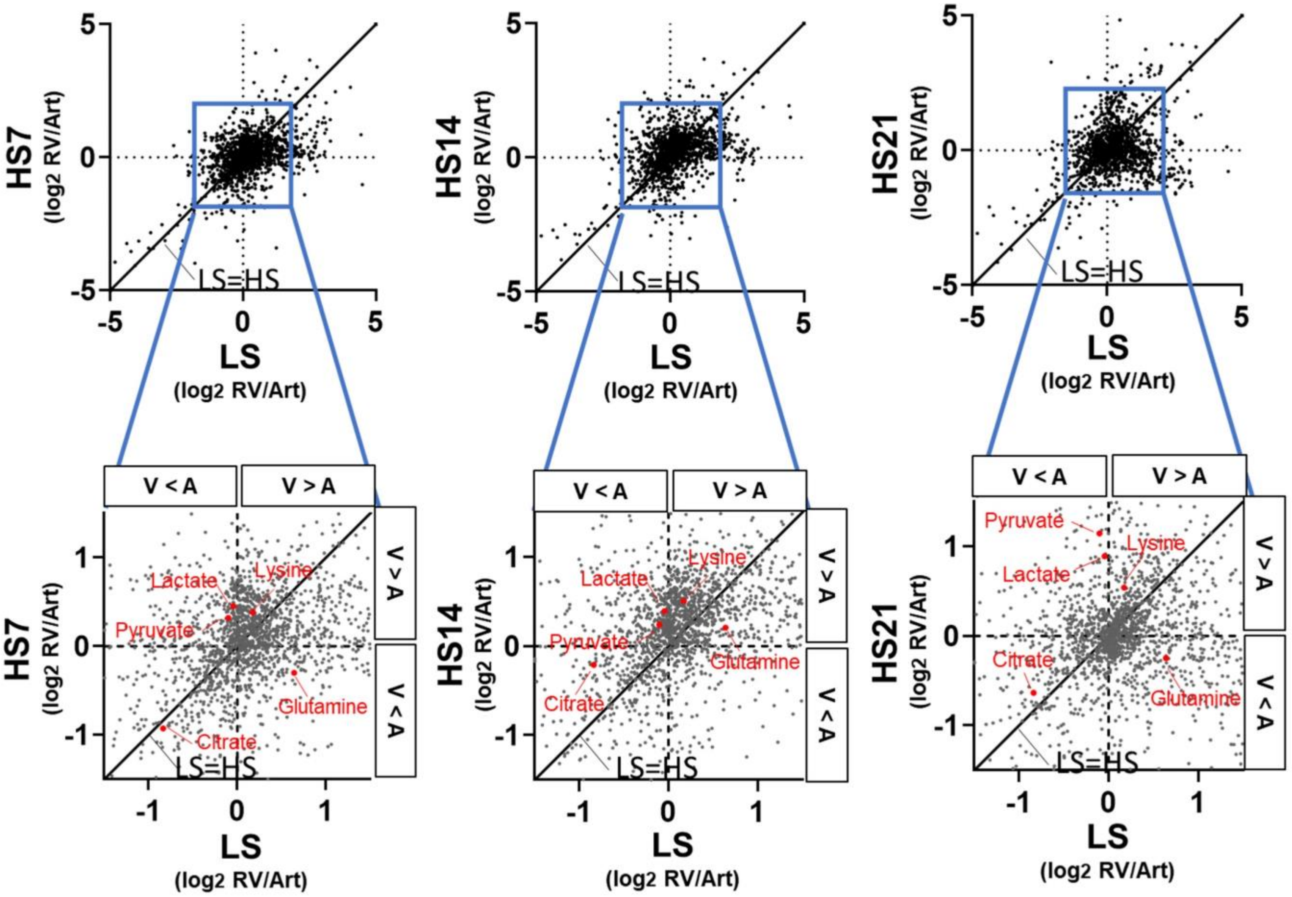

Figure S17. Scatter plot of RV-Art ratio in metabolomics

The binomial logarithm of the RV-Art ratio of metabolite concentrations is represented in scatter plot. Horizontal axis is LS and vertical axis is HS. Black or gray dots indicate each metabolite, and red dots indicate several of carbohydrates or amino acids. The dotted line is the line of 0 (Art=V). The diagonal solid line represents the line LS=HS.

Figure S18.

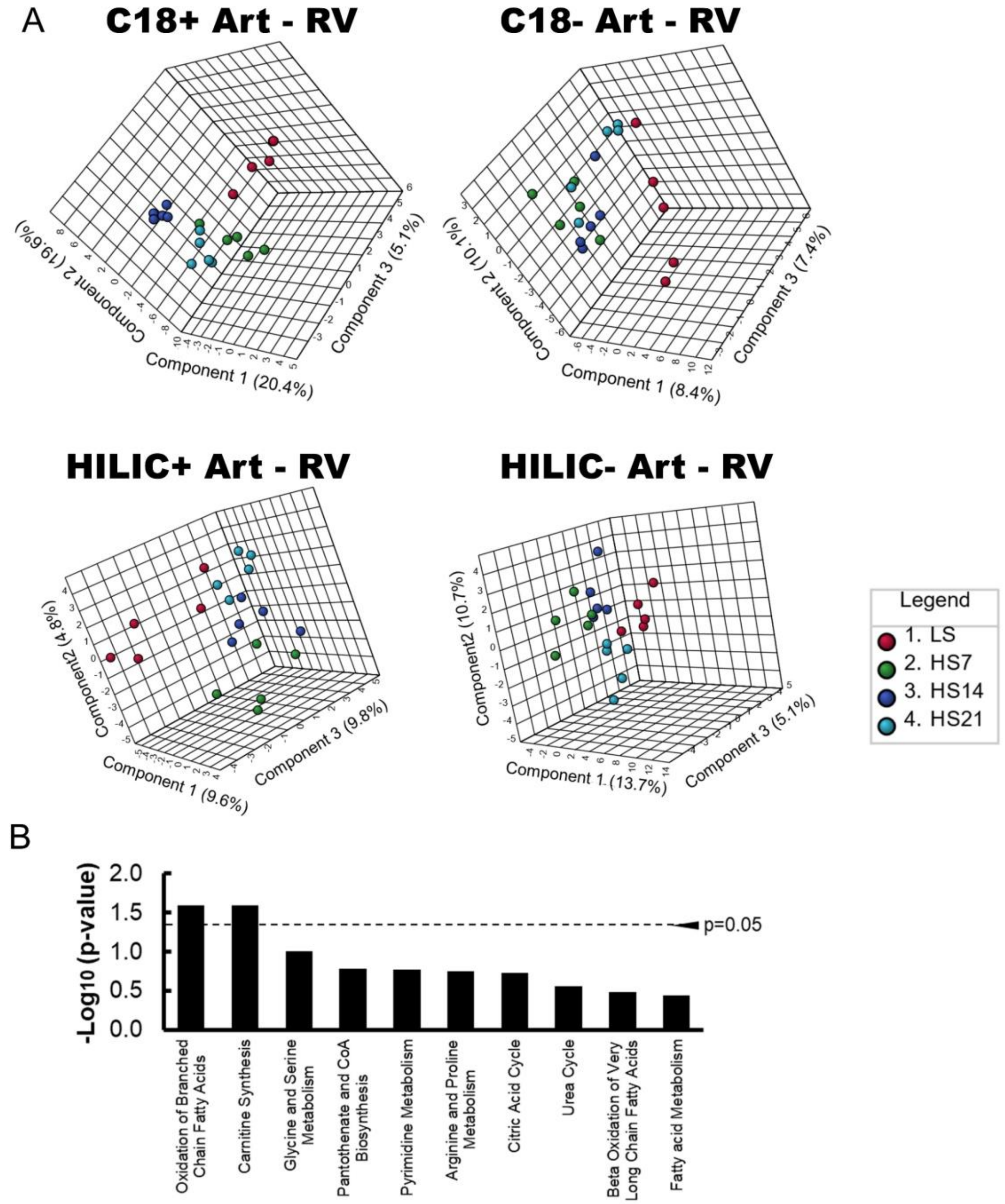

Figure S18. sPLS-DA and pathway analysis of arterial and venous difference

(A) Sparse Partial Least-Squares Discriminant Analysis (sPLS-DA) of arterial and venous plasma differences in each of 4 modes (C18+/-, HILIC+/-). Parameters for sPLS-DA are fixed to number of components: 5, variables per component: 20 and validation method: 5-fold CV. Red: Low salt (LS), green: 7 days of high salt (HS), blue: 14 days of HS and light blue: 21 days of HS. (B) Top 10 pathways in order of $-\log_{10}$ (p-value) by enrichment analysis on Metaboanalyst 5.0 (SMPDB, November 2022). Metabolites which are significantly altered by linear models with covariate adjustment are analyzed.

Figure S19.

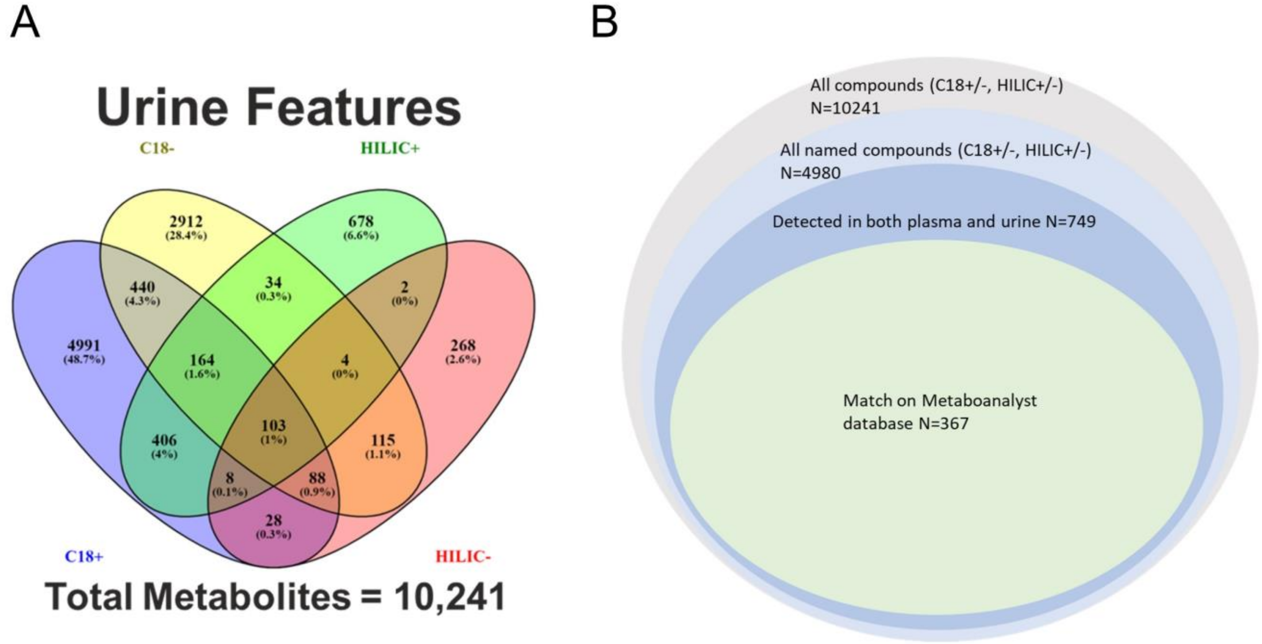

Figure S19. Metabolite features in urine

Venn diagram shows the number of metabolites detected in each of 4 modes (C18+/-, HILIC+/-) in urine (A). The number of metabolites detected, named, detected in both urine and plasma, and listed in the Metaboanalyst 5.0 database (November 2022) are also shown(B).

Figure S20.

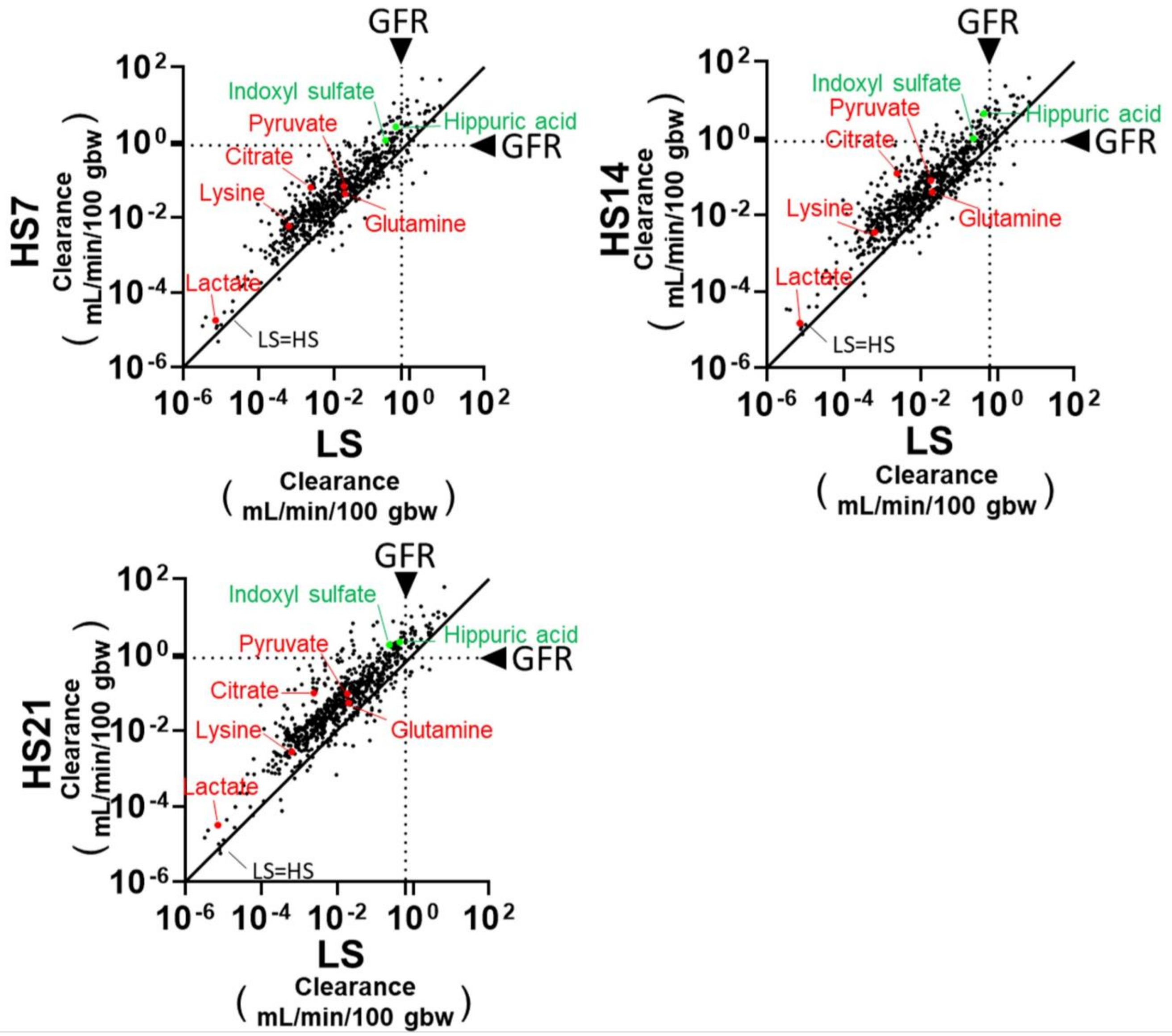

Figure S20. Scatter plot of clearance of metabolites

The scatter plot of clearance of metabolites are shown in log-log graph. The horizontal axis is LS and vertical axis is HS. Black dots indicate each metabolite, red dots indicate several carbohydrates or amino acids and green dots indicate uremic toxins. The dotted line is the line of GFR (Figure 1G). The diagonal solid line represents the line LS=HS.

Figure S21.

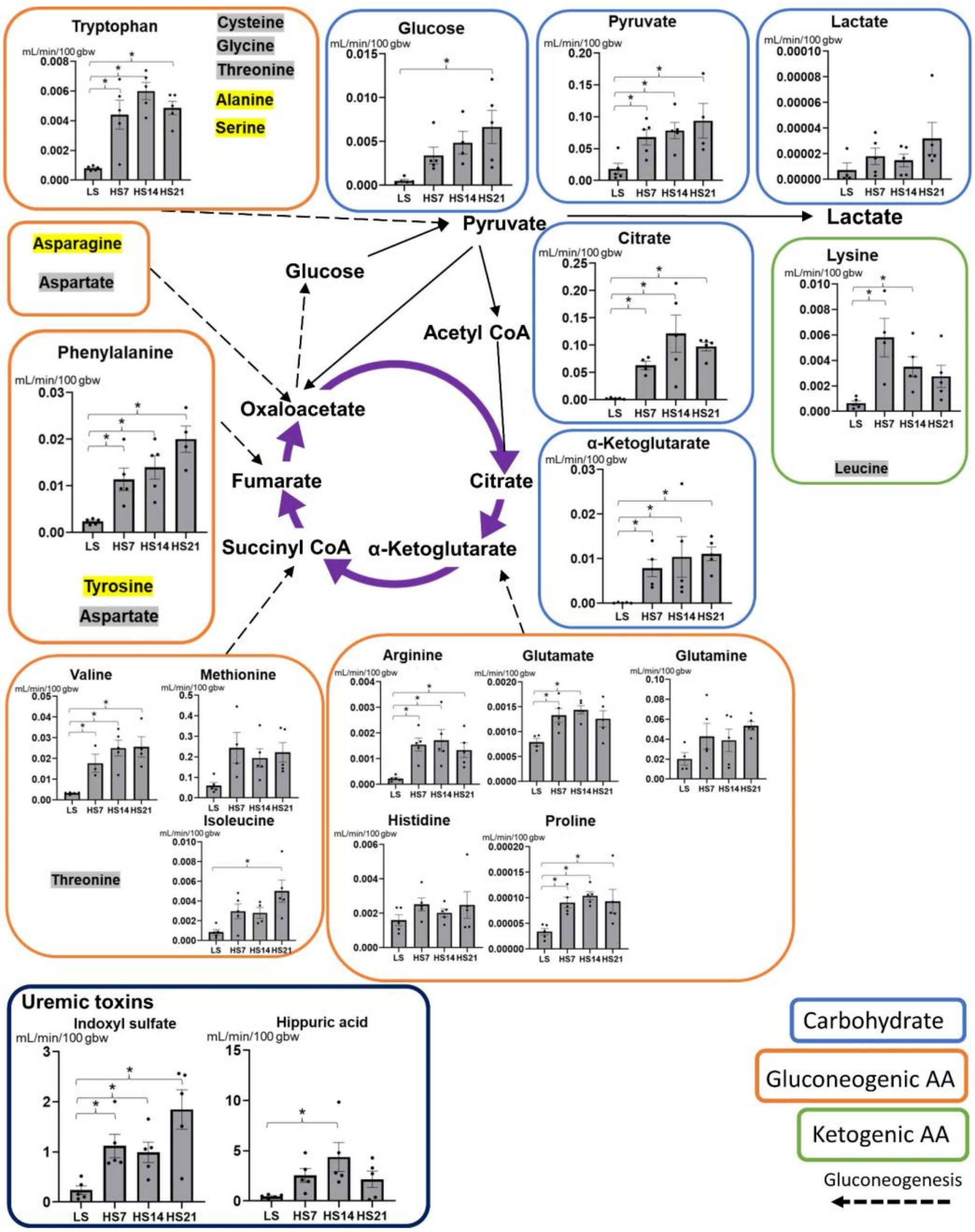

Figure S21. Calculated clearance of metabolites

Mean ± SEM and individual data are shown in each graph. Horizontal line is date, vertical line is clearance. *p<0.05 vs low salt (LS), one-way RM ANOVA, Holm-Sidak. Blue boxes: carbohydrates and their derivative, orange boxes: gluconeogenic amino acid (AA), green boxes: ketogenic AA. Dark blue boxes: uremic toxins. Purple arrows denote TCA cycle and dash arrows denote gluconeogenesis. Highlighted by yellow are metabolites which are not detected in urine. Highlighted by gray are not detected in plasma. (Adapted the figure of Stryer Biochemistry. 7th edition (2012) [108])

Figure S22.

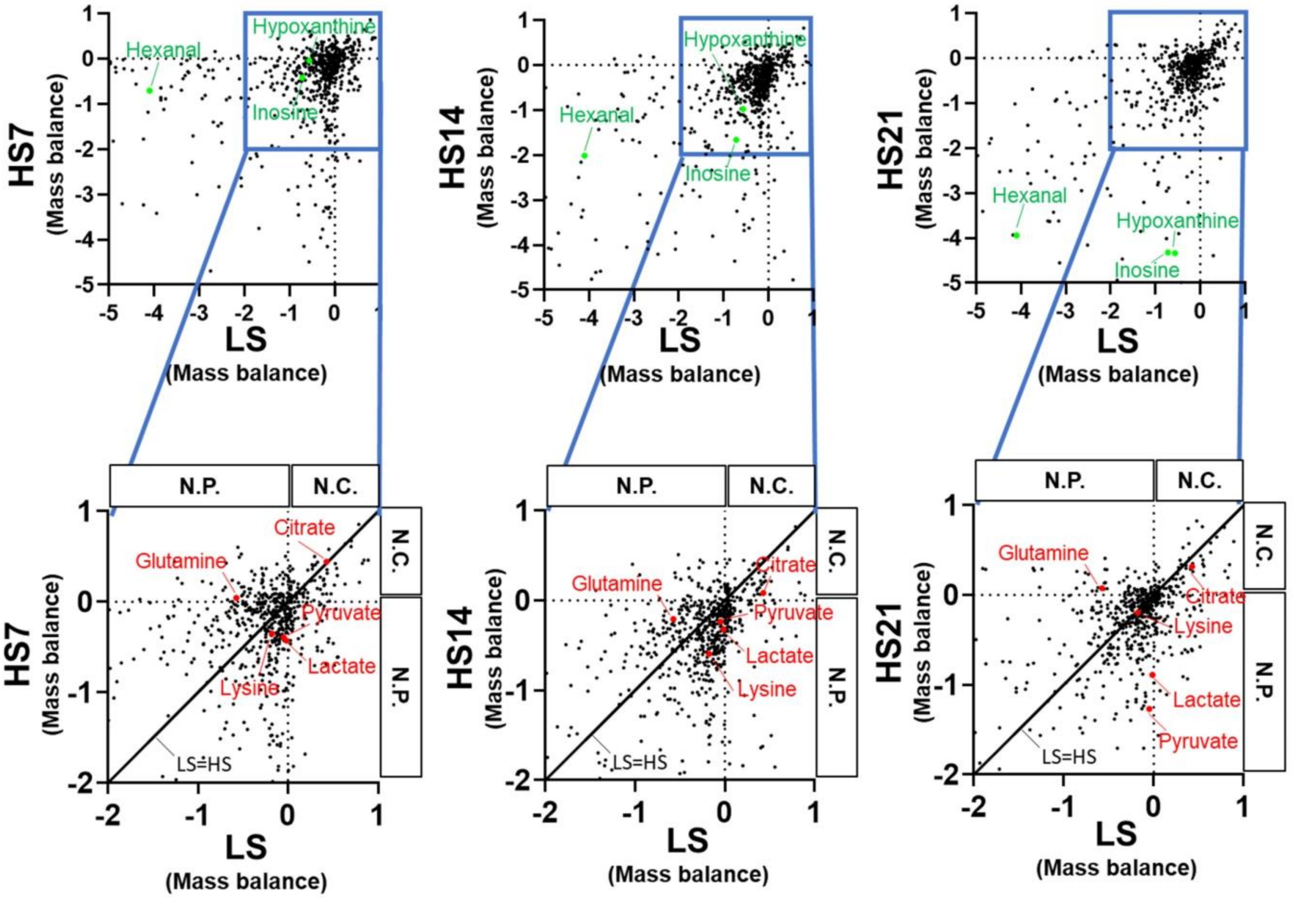

Figure S22. Scatter plot of solute mass balance

Solute mass balance (calculation written in methods. unitless.) is represented in scatter plot. Horizontal axis is LS and vertical axis is HS. Black dots indicate each metabolite, red dots indicate several of carbohydrates or amino acids and green dots indicate uremic toxins. The dotted line is the line of 0 (metabolites inflow = outflow). The diagonal solid line represents the line LS=HS. N.P.: net production, N.C.: net consumption.

Table S1.

	Age (week)	Body weight (BW; g)	Right kidney weight (RKW; g)	Left kidney weight (LKW; g)	RKW/BW (mg/g)	LKW/BW (mg/g)
LS group	15.2 ± 0.5	360 ± 9	1.26 ± 0.02	1.23 ± 0.05	3.50 ± 0.11	3.42 ± 0.12
HS group	15.3 ± 0.5	352 ± 14	1.23 ± 0.05	1.22 ± 0.05	3.50 ± 0.08	3.46 ± 0.09
T-test	N.S.	N.S.	N.S.	N.S.	N.S.	N.S.

Table S1. Body and kidney weight data related to rats studied to determine GFR.
Mean ± SEM of the body weights (BW), right kidney weight (RKW), left kidney weight (LKW) and those ratio to BW of a group fed HS for 21 days (n=6) and of rats of the same age fed only LS (n=5). T-test. $p>0.05$ (N.S.) for all measured parameters.

Table S2.

sample	library	raw_reads	raw_bases	clean_reads	clean_bases	error_rate	Q20	Q30	GC_pct
OM1	CRRA220043341-1a	44849724	6.73G	44407040	6.66G	0.03	97.27	92.89	48.92
OM2	CRRA220043342-1a	40092102	6.01G	39518432	5.93G	0.03	97.16	92.82	49.27
OM3	CRRA220043343-1a	46067838	6.91G	45608770	6.84G	0.03	97.13	92.67	48.96
OM4	CRRA220043344-1a	48007300	7.2G	47461058	7.12G	0.03	97	92.43	48.81
OM5	CRRA220043345-1a	40277514	6.04G	39820890	5.97G	0.03	96.94	92.29	49.34
OM6	CRRA220043346-1a	43049550	6.46G	42544692	6.38G	0.03	97.05	92.53	48.89
OM7	CRRA220043347-1a	48731188	7.31G	48247096	7.24G	0.03	96.78	91.76	48.64
OM8	CRRA220043348-1a	45724866	6.86G	45228734	6.78G	0.03	97.09	92.62	49.32
OM9	CRRA220043349-1a	39416728	5.91G	39015026	5.85G	0.03	96.88	92.04	48.86
OM10	CRRA220043350-1a	23276826	3.49G	22812608	3.42G	0.03	95.97	90.86	47.97
OM11	CRRA220043351-1a	45638432	6.85G	44970914	6.75G	0.03	97.25	92.85	48.91
OM12	CRRA220043352-1a	40053758	6.01G	39550788	5.93G	0.03	96.89	92.07	48.84
OM13	CRRA220043353-1a	45564434	6.83G	44851888	6.73G	0.03	97.17	92.71	48.91
OM14	CRRA220043354-1a	46748222	7.01G	46010598	6.9G	0.03	97.03	92.37	49.15
OM15	CRRA220043355-1a	45962376	6.89G	45213716	6.78G	0.03	97.08	92.45	49.1
C1	CRRA220043356-1a	43902612	6.59G	43362672	6.5G	0.03	96.99	92.32	49.14
C2	CRRA220043357-1a	40996390	6.15G	40467158	6.07G	0.03	96.98	92.26	49.08
C3	CRRA220043358-1a	40879696	6.13G	40338762	6.05G	0.03	97.27	92.91	49.2
C4	CRRA220043359-1a	43601396	6.54G	43084088	6.46G	0.03	97.08	92.51	49.09
C5	CRRA220043360-1a	49130302	7.37G	48729920	7.31G	0.03	97.24	92.86	49.09
C6	CRRA220043361-1a	45374818	6.81G	45007444	6.75G	0.03	97.01	92.34	49.22
C7	CRRA220043362-1a	44445948	6.67G	43846308	6.58G	0.03	97.23	92.63	48.79
C8	CRRA220043363-1a	55083556	8.26G	54434538	8.17G	0.03	97.05	92.48	49.31
C9	CRRA220043364-1a	65551630	9.83G	64841324	9.73G	0.03	97.03	92.46	49.56
C10	CRRA220043365-1a	48192928	7.23G	47760264	7.16G	0.03	97.21	92.81	48.48
C11	CRRA220043366-1a	46733128	7.01G	46257456	6.94G	0.03	97.07	92.53	48.82
C12	CRRA220043367-1a	46349034	6.95G	45724442	6.86G	0.03	97.15	92.58	48.35
C13	CRRA220043368-1a	43751380	6.56G	43231824	6.48G	0.03	97.16	92.73	49.06
C14	CRRA220043369-1a	45532476	6.83G	45035430	6.76G	0.03	96.98	92.22	48.71
C15	CRRA220043370-1a	43588302	6.54G	42911354	6.44G	0.03	97.21	92.74	49.1

Table S2.

Quality control of RNAseq. OM: outer medulla, C: cortex. 1-5: LS, 6-10: HS14, 11-15: HS21.

Table S3.

sample	total_reads	total_map	unique_map	multi_map	read1_map	read2_map	positive_map	negative_map	splice_map	unsplice_map	proper_map
OM1	44407040	41977678(94.53%)	39227938(88.34%)	2749740(6.19%)	19679557(44.32%)	19548381(44.02%)	19594936(44.13%)	19633002(44.21%)	13220586(29.77%)	26007352(58.57%)	37683558(84.86%)
OM2	39518432	37020445(93.68%)	33940418(85.89%)	3080027(7.79%)	17062379(43.18%)	16878039(42.71%)	16958556(42.91%)	16981862(42.97%)	12301024(31.13%)	21639394(54.76%)	32581972(82.45%)
OM3	45608770	43021291(94.33%)	39995044(87.69%)	3026247(6.64%)	20129816(44.14%)	19865228(43.56%)	19981685(43.81%)	20013359(43.88%)	13977274(30.65%)	26017770(57.05%)	38428142(84.26%)
OM4	47461058	44507847(93.78%)	41073366(86.54%)	3434481(7.24%)	20690223(43.59%)	20383143(42.95%)	20520620(43.24%)	20552746(43.3%)	13547402(28.54%)	27525964(58.0%)	39342292(82.89%)
OM5	39820890	37432716(94.0%)	34662145(87.05%)	2770571(6.96%)	17468894(43.87%)	17193251(43.18%)	17317025(43.49%)	17345120(43.56%)	11224806(28.19%)	23437339(58.86%)	33236790(83.47%)
OM6	42544692	40072878(94.19%)	37366414(87.83%)	2706464(6.36%)	18817333(44.23%)	18549081(43.6%)	18667897(43.88%)	18698517(43.95%)	13151004(30.91%)	24215410(56.92%)	35813638(84.18%)
OM7	48247096	45540087(94.39%)	42403657(87.89%)	3136430(6.5%)	21374941(44.3%)	21028716(43.59%)	21180221(43.9%)	21223436(43.99%)	14863389(30.81%)	27540268(57.08%)	40599640(84.15%)
OM8	45228734	42642134(94.28%)	39528056(87.4%)	3114078(6.89%)	19895057(43.99%)	19632999(43.41%)	19748938(43.66%)	19779118(43.73%)	13530807(29.92%)	25997249(57.48%)	37939420(83.88%)
OM9	39015026	36807196(94.34%)	34419650(88.22%)	2387546(6.12%)	17362529(44.5%)	17057121(43.72%)	17194300(44.07%)	17225350(44.15%)	12193026(31.25%)	22226624(56.97%)	32983956(84.54%)
OM10	22812608	20636399(90.46%)	19231853(84.3%)	1404546(6.16%)	9716227(42.59%)	9515626(41.71%)	9616504(42.15%)	9615349(42.15%)	4712741(20.66%)	14519112(63.65%)	18018934(78.99%)
OM11	44970914	42488097(94.48%)	39745719(88.38%)	2742378(6.1%)	19926216(44.31%)	19819503(44.07%)	19851868(44.14%)	19893851(44.24%)	13723222(30.52%)	26022497(57.87%)	38152818(84.84%)
OM12	39550788	37239109(94.16%)	34870257(88.17%)	2368852(5.99%)	17598100(44.49%)	17272157(43.67%)	17418767(44.04%)	17451490(44.12%)	12104639(30.61%)	22765618(57.56%)	33329892(84.27%)
OM13	44851888	42426809(94.59%)	39545463(88.17%)	2881346(6.42%)	19907994(44.39%)	19637469(43.78%)	19756206(44.05%)	19789257(44.12%)	14400049(32.11%)	25145414(56.06%)	37932238(84.57%)
OM14	46010598	43399390(94.32%)	40455869(87.93%)	2943521(6.4%)	20348556(44.23%)	20107313(43.7%)	20211154(43.93%)	20244715(44.0%)	15023464(32.65%)	25432405(55.28%)	38765036(84.25%)
OM15	45213716	42533761(94.07%)	39507262(87.38%)	3026499(6.69%)	19835836(43.87%)	19671426(43.51%)	19736281(43.65%)	19770981(43.73%)	14350255(31.74%)	25157007(55.64%)	37869688(83.76%)
C1	43362672	40828791(94.16%)	37634173(86.79%)	3194618(7.37%)	18972907(43.75%)	18661266(43.04%)	18800197(43.36%)	18833976(43.43%)	13462311(31.05%)	24171862(55.74%)	36067814(83.18%)
C2	40467158	38366106(94.81%)	35771886(88.4%)	2594220(6.41%)	18040058(44.58%)	17731828(43.82%)	17870159(44.16%)	17901727(44.24%)	13159173(32.52%)	22612713(55.88%)	34361070(84.91%)
C3	40338762	38371551(95.12%)	35830841(88.82%)	2540710(6.3%)	18027389(44.69%)	17803452(44.13%)	17899798(44.37%)	17931043(44.45%)	13552046(33.6%)	22278795(55.23%)	34493842(85.51%)
C4	43084088	40915520(94.97%)	38278042(88.84%)	2637478(6.12%)	19301886(44.8%)	18976156(44.04%)	19122126(44.38%)	19155916(44.46%)	14538186(33.74%)	23739856(55.1%)	36740252(85.28%)
C5	48729920	46345180(95.11%)	43321716(88.9%)	3023464(6.2%)	21807729(44.75%)	21513987(44.15%)	21642760(44.41%)	21678956(44.49%)	16384906(33.62%)	26936810(55.28%)	41701490(85.58%)
C6	45007444	42689392(94.85%)	39940155(88.74%)	2749237(6.11%)	20139704(44.75%)	19800451(43.99%)	19953580(44.33%)	19986575(44.41%)	15128306(33.61%)	24811849(55.13%)	38305252(85.11%)
C7	43846308	41811515(95.36%)	38981705(88.91%)	2829810(6.45%)	19535659(44.55%)	19446046(44.35%)	19470385(44.41%)	19511320(44.5%)	14422920(32.89%)	24558785(56.01%)	37582204(85.71%)
C8	54434538	51234608(94.12%)	47155167(86.63%)	4079441(7.49%)	23750456(43.63%)	23404711(43.0%)	23557995(43.28%)	23597172(43.35%)	17297814(31.78%)	29857353(54.85%)	45239810(83.11%)
C9	64841324	61098782(94.23%)	56410821(87.0%)	4687961(7.23%)	28429419(43.84%)	27981402(43.15%)	28183252(43.46%)	28227569(43.53%)	21369969(32.96%)	35040852(54.04%)	54189952(83.57%)
C10	47760264	45314956(94.88%)	42209192(88.38%)	3105764(6.5%)	21242114(44.48%)	20967078(43.9%)	21086517(44.15%)	21122675(44.23%)	14456217(30.27%)	27752975(58.11%)	40560414(84.93%)
C11	46257456	43755409(94.59%)	40782502(88.16%)	2972907(6.43%)	20538215(44.4%)	20244287(43.76%)	20371868(44.04%)	20410634(44.12%)	14145417(30.58%)	26637085(57.58%)	39154530(84.64%)
C12	45724442	43246909(94.58%)	40354467(88.26%)	2892442(6.33%)	20276828(44.35%)	20077639(43.91%)	20161045(44.09%)	20193422(44.16%)	13209943(28.89%)	27144524(59.37%)	38723674(84.69%)
C13	43231824	41028746(94.9%)	38397141(88.82%)	2631605(6.09%)	19323677(44.7%)	19073464(44.12%)	19182731(44.37%)	19214410(44.45%)	13277141(30.71%)	25120000(58.11%)	36871548(85.29%)
C14	45035430	42554388(94.49%)	39834525(88.45%)	2719863(6.04%)	20049791(44.52%)	19784734(43.93%)	19897839(44.18%)	19936686(44.27%)	13568801(30.13%)	26265724(58.32%)	38181004(84.78%)
C15	42911354	40583342(94.57%)	37918513(88.36%)	2664829(6.21%)	19038646(44.37%)	18879867(44.0%)	18941819(44.14%)	18976694(44.22%)	13163870(30.68%)	24754643(57.69%)	36368258(84.75%)

Table S3.

Mapping results of RNAseq. OM: outer medulla, C: cortex. 1-5: LS, 6-10: HS14, 11-15: HS21.

Table S4.

sample	exon	intron	intergenic
OM1	4449279834(70.8462%)	885452918(14.0991%)	945463845(15.0547%)
OM2	4064089108(73.3861%)	582891098(10.5254%)	890974535(16.0885%)
OM3	4676669362(72.6566%)	799524749(12.4214%)	960485464(14.9221%)
OM4	4684167927(70.3488%)	880857417(13.2291%)	1093466550(16.4221%)
OM5	4035249822(72.0564%)	664576394(11.8672%)	900301754(16.0764%)
OM6	4486480142(74.8374%)	643160063(10.7283%)	865334098(14.4343%)
OM7	5299272637(77.7767%)	565778666(8.3039%)	948397614(13.9195%)
OM8	4851322848(76.0437%)	567890157(8.9016%)	960436760(15.0547%)
OM9	4120209616(74.8235%)	630090670(11.4425%)	756270681(13.7340%)
OM10	1688070082(54.7270%)	791016083(25.6446%)	605442380(19.6284%)
OM11	4622304649(72.7201%)	826205273(12.9982%)	907778690(14.2816%)
OM12	4066412161(72.9958%)	694843185(12.4731%)	809489746(14.5311%)
OM13	4885121627(76.9687%)	554720278(8.7400%)	907054119(14.2913%)
OM14	5035724882(77.5608%)	555574042(8.5570%)	901317851(13.8822%)
OM15	4864296903(76.4492%)	566687710(8.9063%)	931801389(14.6446%)
C1	4592641984(75.1852%)	596979783(9.7730%)	918819133(15.0418%)
C2	4446240378(77.4559%)	543478583(9.4677%)	750634579(13.0765%)
C3	4527971119(78.8736%)	493856510(8.6026%)	718967134(12.5238%)
C4	4831461668(78.9277%)	524452997(8.5676%)	765462521(12.5047%)
C5	5427979495(78.2748%)	625859315(9.0253%)	880684161(12.7000%)
C6	4990461737(78.1362%)	586930074(9.1896%)	809481377(12.6741%)
C7	4909233745(78.4753%)	539848586(8.6296%)	806684134(12.8950%)
C8	5853469641(76.3602%)	672671690(8.7752%)	1139463450(14.8646%)
C9	6973289237(76.2805%)	823743548(9.0109%)	1344610213(14.7086%)
C10	5188281108(76.5323%)	701235368(10.3439%)	889679867(13.1237%)
C11	4980576852(76.0877%)	670440114(10.2422%)	894822364(13.6701%)
C12	4901632876(75.7685%)	684146860(10.5754%)	883438344(13.6560%)
C13	4688000447(76.3741%)	634500092(10.3369%)	815708677(13.2890%)
C14	4875022937(76.5759%)	642806789(10.0971%)	848434992(13.3270%)
C15	4657728178(76.7247%)	606107952(9.9842%)	806863697(13.2911%)

Table S4.

Mapping region of RNAseq. OM: outer medulla, C: cortex. 1-5: LS, 6-10: HS14, 11-15: HS21.

Table S5.

Analysis	Software	Version	Parameter	Remarks
Mapping	hisat2	2.0.5	Default	Mapping to a reference
Assembly	Stringtie	1.3.3b	Transcripts of class code type 'u'	
Quantification	featureCounts	1.5.0-p3	Default	
Differential Analysis	DESeq2	1.20.0	|log2(FoldChange)| >= 1 & padj <= 0.05	For sample with bio-replicate
	edgeR	3.22.5	|log2(FoldChange)| >= 1 & padj <= 0.05	For sample without bio-replicate
Enrichment Analysis	clusterProfiler	3.8.1	padj < 0.05	For GO, KEGG enrichment analysis
GSEA Analysis	gsea	v3.0		
Protein-Protein Interaction Analysis	diamond	0.9.14	Using blast, String database.	
Alternative splicing	rMATS	4.1.0	Default	
SNP/InDel Analysis	GATK	4.1.4.1	MQ < 40.0 and QD < 2.0 and FS > 30.0 and DP < 10 and QUAL < 20	SNP/InDel calling
	snpEff	4.3.1q	Default	SNP/InDel Annotation

Table S5.

Software and parameters used for RNAseq analysis.

Table S6

Gene		Sequence or QIAGEN ID
18s	*F*	CGGCTACCACATCCAAGGAA
18s	*R*	CCTGTATTGTTATTTTTCGTCACTACCT
Aqp1		QT00173789
Hk1		PPR45173E-200
Hk2		PPR52902A-200
Hk3		PPR50411A-200
Ldha		PPR56603B-200
Nphs2	*F*	GAGCGAGCGACCAGAGGAA
Nphs2	*R*	AAGGGAAACGTTACGATGATGAAG
Pkm	*F*	TGAAGTACGCCCGAGGATCT
Pkm	*R*	TTCAGCCGAGCCACATTCAT
Scnn1a	*F*	TCCTGACCATGCACCATCAC
Scnn1a	*R*	CCCTGCAACCAGGCGAATTA
Slc12a1 (Nkcc2)	*F*	CCTTGTCTGAGCTTGAGAATTACGT
Slc12a1 (Nkcc2)	*R*	CCCTTGGCTGATTCTGCTATGA

Table S6. Information of primers for qPCR

Table S7.

	Age (week)	GFR (mL/min/100 gbw)	Body weight (g)	Hct (%)	Half life of sinistrin (min)
LS	12.3 ± 0.5	0.64 ± 0.04	305 ± 13	39 ± 1	33.9 ± 2.0
HS7	13.3 ± 0.5	0.83 ± 0.05*	320 ± 12*	41 ± 1 N.S.	26.1 ± 1.5*
HS14	14.3 ± 0.5	0.81 ± 0.06*	339 ± 12*	41 ± 1 N.S.	27.1 ± 2.0*
HS21	15.3 ± 0.5	0.85 ± 0.05*	352 ± 14*	43 ± 1*	25.6 ± 1.4*

Table S7. GFR measurements of unanesthetized SD rats studied at LS and at 7, 14, and 21 days of the HS diet.

N=6. Mean ± SEM of age, GFR, body weight, hematocrit (Hct) and half-life of sinistrin.

*p<0.05 vs LS, One-way RM ANOVA, Holm-Sidak. N.S.: p>0.05

Table S8.

	RBF (12h daytime) (mL/min/100 gbw)	RVR (12h daytime) (mmHg/mL/min/100 gbw)	O_2 Consumption (mL/min/kgbw)	O_2 Delivery (mL/min/kgbw)
LS	3.03 ± 0.18	37.2 ± 1.5	0.495 ± 0.057	4.88 ± 0.22
HS7	3.38 ± 0.18*	35.9 ± 1.8 N.S.	0.638 ± 0.033*	5.40 ± 0.33 N.S.
HS14	3.36 ± 0.17*	36.2 ± 2.0 N.S.	0.630 ± 0.061*	5.31 ± 0.27 N.S.
HS21	3.33 ± 0.18*	36.2 ± 1.0 N.S.	0.741 ± 0.035*	5.31 ± 0.34 N.S.

Table S8. Renal blood flow (RBF), calculated renal vascular resistance (RVR), O_2 consumption, and O_2 delivery normalized by body weight of the GFR group of rats.

N=6. Mean ± SEM, *p<0.05 vs LS, One-way RM ANOVA, Holm-Sidak. N.S.: p>0.05

Table S9.

Renal Vein									
	Hb (g/dL)	pO2 (mmHg)	SHbO₂ (%)	O_2 content (mL/dL)	pCO2 (mmHg)	Na+ (mM)	K+ (mM)	Cl- (mM)	Ca++ (mM)
LS	13.8 (0.5)	77.4 (3.0)	86.4 (1.1)	15.8 (0.4)	35.2 (1.6)	144.8 (0.5)	3.3 (0.1)	115.0 (1.3)	1.3 (0.2)
HS7	13.8 N.S. (0.4)	75.8 N.S. (1.1)	83.8 N.S. (0.9)	15.4 N.S. (0.5)	35.7 N.S. (1.0)	144.7 N.S. (0.7)	3.4 N.S. (0.1)	112.3 N.S. (1.3)	1.1 N.S. (0.0)
HS14	13.5 N.S. (0.4)	72.8 N.S. (1.3)	82.4* (1.1)	14.8* (0.5)	36.1 N.S. (2.2)	144.5 N.S. (1.1)	3.7* (0.1)	116.5 N.S. (3.3)	1.0 N.S. (0.1)
HS21	13.3 N.S. (0.4)	69.1 N.S. (2.7)	81.4* (1.0)	14.4* (0.6)	34.5 N.S. (1.2)	145.5 N.S. (1.1)	3.6 N.S. (0.1)	116.2 N.S. (2.8)	1.0 N.S. (0.0)

Artery									
	Hb (g/dL)	pO2 (mmHg)	SHbO₂ (%)	O_2 content (mL/dL)	pCO2 (mmHg)	Na+ (mM)	K+ (mM)	Cl- (mM)	Ca++ (mM)
LS	13.4 (0.2)	116.8 (2.9)	95.6 (0.6)	17.6 (0.6)	32.0 (2.4)	146.2 (0.9)	3.0 (0.1)	117.8 (2.4)	1.3 (0.1)
HS7	13.6 N.S. (0.5)	110.3* (2.9)	94.8 N.S. (0.3)	17.5 N.S. (0.4)	34.4 N.S. (1.2)	145.5 N.S. (0.6)	3.1 N.S. (0.1)	113.0 N.S. (1.6)	1.1 N.S. (0.0)
HS14	14.1 N.S. (0.5)	104.2* (1.8)	93.4* (0.3)	16.9 N.S. (0.5)	35.4 N.S. (2.0)	144.7 N.S. (1.3)	3.3 N.S. (0.1)	115.2 N.S. (3.9)	1.2 N.S. (0.1)
HS21	13.7 N.S. (0.4)	109.3* (2.9)	94.1* (0.5)	16.7 N.S. (0.5)	33.1 N.S. (1.6)	145.8 N.S. (1.4)	3.3 N.S. (0.2)	114.7 N.S. (3.1)	1.1 N.S. (0.1)

Table S9. Arterial and renal venous blood gas data.

Total Hemoglobin (Hb), partial pressure of oxygen (pO2), oxyhemoglobin saturation (SHbO2), O_2 content and whole blood electrolyte data obtained from the unanesthetized instrumented rats when fed LS, and HS for 7, 14, and 21 days. N=6, Mean and (SEM), *p<0.05 vs LS, One-way RM ANOVA, Holm-Sidak. N.S.: p>0.05 (N.S.), mM: mmol/L

Table S10.

	FISH		FISH		FISH
(2E,4E)-2,4-Dodecadienal	67.4	Dibutyl phthalate	25.5	Sebacic acid	12.3
(3E)-4-(4-hydroxyphenyl)but-3-en-2-one	42.1	Diethylene glycol	16.7	Tartaric acid	10
(3β,5α,9α,22E,24R)-3,5,9-Trihydroxy-23-methylergosta-7,22-dien-6-one	76.9	Dihydrothymine	38.6	Taurochenodesoxycholic acid	27.9
1,11-Undecanedicarboxylic acid	30.5	Dodecanoic acid	20.8	Tetraethylene glycol	11.5
1,2-Benzisothiazol-3(2H)-one	25.0	D-Pipecolic acid	57.1	Thiorphan	81.8
1,2-Dihydro-1,1,6-trimethylnaphthalene	68.2	D-Proline	52.6	trans-Aconitic acid	20.8
2,2-Bis[4-(2,3-epoxypropoxy)phenyl]propane	21.2	Eicosapentaenoic acid	33.3	Traumatic acid	15.4
2,5-Furandicarboxylic acid	13.6	Ethyl lactate	21.3	Tridecanoic acid	67.7
2-Acetyl-4-methylpyridine	15.4	Etodolac	14.8	Triethylamine	23.8
2-Amino-9,10-epoxy-8-oxodecanoic acid	19.1	gamma-Asarone	37.5	Trimethylamine N-oxide	26.7
2-Diethylaminoethanol	50	Glycocholic acid	30.4	Ureidopropionic acid	30
2-Ethylglutaric acid	24.6	Hexylbenzene	44.4	Uric acid	38.6
3,3-Dimethylglutaric acid	17.4	Hydroxyphenyllactic acid	46.7	Valproic acid	12.1
3,4-Dihydroxyhydrocinnamic acid	46.7	Indole-3-methyl acetate	40	Violet-leaf aldehyde	42.3
3,4-Methylenesebacic acid	12.5	Indoleacetic acid	22.2	α-Ketoglutaric acid	25
3-Buten-1-amine	62.5	Isovaleric acid	31	(+)-Gallocatechin	0
3-Hydroxycapric acid	12.5	L-Glutamine	32.2	1-Methylguanine	5.2
3-Hydroxytetradecanedioic acid	72.2	L-Histidinal	39.9	2,4,6-Octatriyn-1-ol	9.5
3-hydroxytridecanoic acid	50	L-Homoserine	39.5	2-acetyl-1-alkyl-sn-glycero-3-phosphocholine	0
3-Oxooctanoic acid	29.2	L-Isoleucine	61.5	2-Methoxyestrone	4.76
4-Acetamidobenzoic acid	22.2	L-Kynurenine	47.5	4-Hydroxy-3-methoxybenzenemethanol	6.85
4-Hydroxybenzaldehyde	19.5	L-Lactic acid	50	4-Trimethylammoniobutanoic acid	7.41
4-Hydroxybutyric acid	22.6	L-Lysine	59.1	5-hydroxy-2-oxo-4-ureido-2,5-dihydro-1H-imidazole-5-carboxylate	3.33
5-Hydroxy-2-furoic acid	21.4	L-Methionine	56.5	5-Methylcytidine	7.14
5-Methylcytosine	21.9	L-Norleucine	50	9-cis-Retinoic acid	10
7-Oxoheptanoic acid	48.2	L-Tryptophan	81.8	Betaine	0
8-Amino-7-oxononanoic acid	55.6	L-Tyrosine	71.4	Betonicine	3.23
Adipic acid	50	L-Valine	50	Butenylcarnitine	4.17
Allantoin	22	N-(5-Methyl-3-oxohexyl)alanine	51.2	Cytidine monophosphate	0
Aspirin	14.6	N-Desmethyltramadol	25	Decarbamoylsaxitoxin	0
Asymmetric dimethylarginine	60	N-methyl-L-glutamic Acid	50.4	Hexanoylcarnitine	0
Benzaldehyde	27.3	Norophthalmic acid	46.7	L-Acetylcarnitine	0
Benzoic acid	28.6	N-Undecanoylglycine	74.4	L-Carnitine	0
Bz-Arg-OEt	50	Ornithine	43.8	L-Dihydroanticapsin	3.13
Capsiamide	42.9	Pantothenic acid	60.9	L-Palmitoylcarnitine	0
Cepanone	64.5	p-Cresol sulfate	42.9	LysoPC(22:5(7Z,10Z,13Z,16Z,19Z))	0
Cholic acid	69.6	Pelargonic acid	33.3	Methylphosphate	0
Citric acid	10.5	Pentylbenzene	21.6	Nalidixic Acid	0
Citrulline	28.4	Phenylacetylglycine	20	o-Xylene	5.88
Creatine	48.1	Prostaglandin B1	10.7	Phthalic acid	8.51
Creatinine	15.6	Pyridoxamine	12.5	Propionylcarnitine	2.7
Cyclohexylamine	19.3	Pyrrolidonecarboxylic acid	40	Thiamine	0
Cytidine	30.8	Pyruvic acid	12.5	Trimethadione	0
Deoxycytidine	46.7	Salicylic acid	22.2		

Table S10. A-V significantly changed metabolites over time by linear models with covariate adjustments.

All of the n=131 metabolites which are matched on Metaboanalyst 5.0 database (November 2022) and whose arterial and venous plasma differences are significantly ($p<0.05$) changed by high salt (HS) over time by linear models with covariate adjustments. Shaded compounds are FISh score less than 10 (low reliability).

Table S11.

Metabolites detected in both plasma and urine N=367							
Metabolites excreted in urine in excess of filtration fraction							
LS (N=19)	FISH (A/U)	HS7 (N=29)	FISH (A/U)	HS14 (N=37)	FISH (A/U)	HS21 (N=39)	FISH (A/U)
(S)-Pinocembrin	38/44	(S)-Pinocembrin	38/44	(S)-Pinocembrin	38/44	(S)-Pinocembrin	38/44
2-Diethylaminoethanol	50/53	2-Diethylaminoethanol	50/53	2-Diethylaminoethanol	50/53	2-Diethylaminoethanol	50/53
2-Hydroxystearic acid	33/40	2-Hydroxystearic acid	33/40	2-Hydroxystearic acid	33/40	2-Hydroxystearic acid	33/40
Biotin	39/43	Biotin	39/43	Biotin	39/43	Biotin	39/43
Capryloylglycine	35/54	Capryloylglycine	35/54	Capryloylglycine	35/54	Capryloylglycine	35/54
Tartaric acid	53/64	Tartaric acid	53/64	Tartaric acid	53/64	Tartaric acid	53/64
L-Methionine	38/54	L-Methionine	38/54	L-Methionine	38/54	L-Methionine	38/54
Phenylacetylglycine	20/33	Phenylacetylglycine	20/33	Phenylacetylglycine	20/33	Phenylacetylglycine	20/33
Tyramine	50/40	Tyramine	50/40	Tyramine	50/40	Tyramine	50/40
Uric acid	39/30	Uric acid	39/30	Uric acid	39/30	Uric acid	39/30
Methylsuccinic acid	27/40	3-Hydroxyanthranilic acid	19/26	3-Hydroxyanthranilic acid	19/26	3-Hydroxyanthranilic acid	19/26
		Alanyl-Isoleucine	40/51	Alanyl-Isoleucine	40/51	Alanyl-Isoleucine	40/51
		Cyclohexylamine	44/50	Cyclohexylamine	44/50	Cyclohexylamine	44/50
		Hippuric acid	25/39	Hippuric acid	25/39	Hippuric acid	25/39
		Suberic acid	31/45	Suberic acid	31/45	Suberic acid	31/45
		Bethanidine	33/19	17a-Ethynylestradiol	14/22	17a-Ethynylestradiol	14/22
		Hexanal	16/24	4-Oxoproline	17/18	4-Oxoproline	17/18
				Dethiobiotin	46/67	Dethiobiotin	46/67
				Methylglutaric acid	12/19	Methylglutaric acid	12/19
				Oxoglutaric acid	25/28	Oxoglutaric acid	25/28
				Uracil	32/27	Uracil	32/27
				Pantothenic acid	41/69	Aromadendrin	68/29
						Hypoxanthine	32/41
						Inosine	18/56
						Methylsuccinic acid	27/40
						Serotonin	15/13
Kynurenic acid	0/20	Kynurenic acid	0/20	Kynurenic acid	0/20	Kynurenic acid	0/20
N-Acetylvaline	0/33	N-Acetylvaline	0/33	N-Acetylvaline	0/33	N-Acetylvaline	0/33
Nalidixic Acid	0/5	Nalidixic Acid	0/5	Nalidixic Acid	0/5	Nalidixic Acid	0/5
Porphobilinogen	0/10	Porphobilinogen	0/10	Porphobilinogen	0/10	Porphobilinogen	0/10
Pseudouridine	3/1	Pseudouridine	3/1	Pseudouridine	3/1	Pseudouridine	3/1
Triacetin	0/2	Triacetin	0/2	Triacetin	0/2	Triacetin	0/2
Propionylcarnitine	3/0	Propionylcarnitine	3/0	Propionylcarnitine	3/0		
Tiglylcarnitine	0/2	Creatinine	16/2	Creatinine	16/2	Creatinine	16/2
		Guaifenesin	5/25	Guaifenesin	5/25	Guaifenesin	5/25
		Indoxyl sulfate	9/13	Indoxyl sulfate	9/13	Indoxyl sulfate	9/13
		L-Dihydroanticapsin	0/2	L-Dihydroanticapsin	0/2	L-Dihydroanticapsin	0/2
		N-Acetylarylamine	8/3	N-Acetylarylamine	8/3	N-Acetylarylamine	8/3
				Indole	0/13	Indole	0/13
				acid	0/2	methoxybenzenemethanol	7/20
				Glutarylcarnitine	0/4		

Table S11. Urinary metabolism whose excretion is higher than filtration fraction

The number of metabolites detected in both plasma and urine, and that excreted in excess of filtration fraction are shown.

All of the metabolites which excreted in excess of filtration fraction are listed. Shaded compounds are FISh score less than 10 (low reliability).

Table S12.

	Artery (Art) (mM)	Renal vein (RV) (mM)	Art-RV (mM)
LS	1.6 ± 0.8	1.3 ± 0.3	0.4 ± 0.6
HS7	0.9 ± 0.2 N.S.	1.0 ± 0.1N.S.	-0.1 ± 0.1 N.S.
HS14	0.7 ± 0.1 N.S.	0.9 ± 0.1 N.S.	-0.1 ± 0.1 N.S.
HS21	0.8 ± 0.1 N.S	1.1 ± 0.1 N.S.	-0.2 ± 0.1 N.S.

Table S12. Validation of lactate concentration in arterial and renal venous plasma by fluorometric assay kit. Mean ± SEM and individual data. One-way ANOVA, No significant difference ($p>0.05$, n.s.) between groups. mM: mmol/L
